# Forecast on Africa’s power production up to 2030 with related water use and CO_2_ emissions

**DOI:** 10.1038/s41467-026-72692-2

**Published:** 2026-05-07

**Authors:** S. D. Vaca-Jiménez, P. W. Gerbens-Leenes, Bunyod Holmatov, Raphael Vanham, Davy Vanham

**Affiliations:** 1 Departamento de Ingeniería Mecánica, Escuela Politécnica Nacional, Quito, Ecuador; 2 Integrated Research on Energy, Environment and Society (IREES), University of Groningen, Groningen, The Netherlands; 3 International Water Management Institute (IWMI), Colombo, Sri Lanka; 4 Department of Mathematics, University of Innsbruck, Innsbruck, Austria

**Keywords:** Hydrology, Water resources

## Abstract

Africa needs to increase electricity production to improve electricity access. For informed decision making, there is a need for reliable, findable, high-quality, open access and spatially distributed power plant data with associated water use and CO_2_ emissions amounts. Here we present a detailed spatial inventory of operational, under construction and planned African power plants from 2020 until 2030, covering 3,139 individual plants, the result of an intensive data mining effort. This inventory forecasts a 57% increase to 1,787,858 Gigawatthours in electricity production from 2023 to 2030. Related water use and CO_2_ emissions increase substantially, showing trade-offs in water and carbon intensity of different fuel types. Africa is stepping up in planning and constructing additional power plants, with renewables’ share growing from 19% to 34%. However, the increase in hydropower puts additional pressure on available water resources. Current power plant construction falls slightly short on commitments in the nationally determined contributions.

## Abstract (web summary)

Africa is stepping up in planning and constructing power plants up to 2030 with renewables’ share growing from 19% to 34%. An inventory of 3139 plants forecasts a 57% increase in electricity production with water use increasing 73% and CO_2_ 19%.

## Introduction

Electricity access in Africa, currently at 57% of the population, is among the lowest in the world, with large variations between countries^1^. Within the Sustainable Development Goals (SDGs), target 7.1 aims at ensuring universal access to affordable, reliable and modern energy services by 2030. Indicator 7.1.1 specifically measures the proportion of the population with access to electricity, showing that Africa is lagging behind other continents.

The African continent is developing fast, though, with a growing production and consumption of electricity^1^. There are, however, large regional differences, with only three countries, South Africa, Egypt and Algeria, producing 60% of Africa’s electricity^1^. Total power production generally is larger in countries with larger gross domestic products (GDPs)^2^. Increasing electricity production and access to electricity is therefore high on the African policy and investment agenda.

African countries need to invest in future power plant expansion in a water-resilient way while accounting for CO_2_ emissions. The electricity system is an important sector within a wider nexus approach, where interlinkages, synergies and trade-offs between different sectors are addressed. Nexus definitions are heterogenous, depending on which systems or sectors—such as energy, biodiversity/ecosystem, water, food, health, climate—are included^3–6^. Here, we address selected components of the energy–water–climate nexus^7–9^, by analysing power plant water use and CO_2_ emissions, as important trade-offs between these two environmental pressures exist^8, 9^.

For informed decision making, there is a need for reliable, findable, high-quality, open-access and spatially distributed power plant data with associated water use and CO_2_ emissions amounts. Currently, there is a persistent lack of such data throughout Africa for the current situation as well as for future projections^10^. Some data are available^11–14^, yet they fall short on providing a continent-wide assessment of existing, under construction and planned power plants, while accounting for their water use and CO_2_ emissions.

Existing power plant databases for Africa include the World Resource Institute (WRI)’s Global Power Plant Database (GPPD)^12^, the Renewable Power Plant database for Africa (RePP Africa)^13^, a database provided by the European Commission for the year 2016^14^, as well as a database by Gerbens-Leenes et al for the year 2020^11^. The first two databases have many fewer entries for Africa than the ones we include (Supplementary Table 1). They also do not include water use or CO_2_ emissions. The GPPD only includes existing power plants, whereas RePP Africa includes some future renewable power plants. The last two databases^11, 14^ do account for water use but not for CO_2_ emissions, and exclude future power plants. Spatially distributed CO_2_ emissions for power plants have been computed, such as the global CO_2_ emissions catalogue from thermal power plants for the year 2018 by Guevara et al. ^15^, yet none in the spatial resolution for Africa and for the year 2030 forecast we assess. Modelling efforts have shown that globally, water use for energy is increasing, indicating the hotspots where this might occur^16^. However, such studies generally analyse results on the national level and do not provide spatial detail within a country or river basin, nor do they account for actual planning or construction of power plants. The same is true for CO_2_ emissions.

In this paper, we analyse the evolution of Africa-wide electricity production in high spatial resolution until 2030, which is the target year of the SDGs. We first make an inventory of all operational power plants from 2021 to 2023. This extends the recent study by Gerbens-Leenes et al. ^11^. Here, we then identify the operational plants up to 2030 based on publicly available information on decommissioned, planned and projected power plants. We add these power plants, including details on location, year of start of operation, fuel type and other details, into the power plant inventory called INVENTORY, the main output of this paper. It includes 3139 individual power plants and is the most detailed and updated database on power plants covering Africa until 2030 available in the literature

It is not the result of a model. It is the result of an intensive, time-consuming data mining effort of open-access data sources. We used only open-access sources, to be able to provide our resulting database open access. Data sources include GEM wiki^17^ and Wikipedia^18^, because they provide recent information on power plants, especially on the large ones. Other data sources used were Power Technology^19^, a website on the global energy industry, which also provides information on installed capacity and year of commission. We also used Open Street maps, reports from national ministries or international organizations (e.g., the World Bank), scientific papers, companies’ websites, reports and newspapers, which give information on the opening or closure of specific plants. We checked and adapted the location coordinates of all power plants using Google Maps. Data sources for each power plant are included in Supplementary Tables 2–7.

INVENTORY provides a realistic analysis of existing power plants and an estimate of power plants under construction, because the planning and construction of power plants takes time^20^ and the forecast window up to 2030 is relatively short. According to Sovacool et al. ^20^, nuclear power plants and larger hydropower dams have the longest mean construction times, i.e., 7 and 10 years, respectively. Small run-of-river power plants take less time to construct. Fossil-fuelled thermal plants have a mean construction time of almost 5 years. Solar projects and wind farms show the lowest average construction times of about two and one year, respectively. There is, however, quite a variation on these averages. While our forecast window up to 2030 is only about five years, we are confident that we include almost all planned and under construction power plants, as we restrict ourselves to plants that are currently under construction or are being planned, all to start operations by the latest 2030.

We additionally analyse five other forecasts up to 2030: four based upon historical electricity generation and one based on the electricity mix of the Nationally Determined Contributions (NDCs), in accordance with Article 4, paragraph 12 of the Paris Agreement^21^. As such, we analyse six forecasts. The first forecast is INVENTORY, the second forecast is called NDC. The four additional forecasts are based on the time trends of national electricity generation from the IEA^1^ and IRENA^22^ from 1990 to 2015, as well as on the INVENTORY database until 2023. These are extrapolations to 2030 by means of a linear regression (called REGR_LIN), a polynomial regression (called REGR_POL), a multivariate regression using population and GDP as variables (called REGR_MVAR) and an Autoregressive Integrated Moving Average (ARIMA) analysis (called ARIMA).

Freshwater is a limited resource and its use by different sectors, including the energy sector, leads to water scarcity in many places around the world^23^. Water use is increasing due to a combination of economic development, population growth, urbanization, and other factors. Considering Africa has the largest population growth between now and 2050^24^, the continent stands out as a key region with projected increases in water demand. Water is an important constraint to producing electricity. Thermal power plants use water for cooling or dust suppression, hydropower needs water to move its turbines, and solar panels need water for cleaning^25, 26^. There is a large difference in water use intensity (the amount of water per unit of electricity produced) between different energy sources. On average, in Africa, reservoir hydropower has the highest water intensity, fossil fuels are in the mid-range, and wind and solar renewables have the lowest water intensities^11^. It is therefore important to have insight into the future water requirements for electricity generation within a country when new power plants are planned and constructed.

In this study, we therefore assess the blue water withdrawal (WW) and water consumption (WC) for the 2030 forecasts. Blue water or freshwater refers to water in rivers, lakes, wetlands and aquifers^27^. WW refers to the volume of water extracted from its source (rivers, lakes, aquifers) for any economic activity or sector. WC refers to the portion of WW that is not returned to the original water source after being withdrawn, or that flows to the atmosphere through evaporation.

The generation of electricity also has an impact on climate change, due to CO_2_ emissions, especially when fossil fuels are used. Between 2010 and 2021, electricity generation in Africa doubled, although its share of global CO_2_ emissions remains small (about 3%)^1^. The Paris agreement, introduced in 2015, aims to keep the temperature increase below two degrees of warming compared to pre-industrial levels. Countries that signed the agreement comply with the goals that are formulated in their NDCs^21^. In Africa, 53 countries have submitted an NDC on climate change, which together aim to mitigate 550 Mt of CO_2_ by 2030—equal to 40% of Africa’s emissions today. To comply with the agreement, countries need to write a national strategy on how to decrease their greenhouse gas emissions. These plans often include goals for the electricity mix and express strategies for renewables in terms of installed capacities (MW), not in electricity generation. Twelve African countries, which together represent over 40% of those emissions, have also announced net-zero emissions goals. Many African NDCs include targets that are conditional on financial support from developed countries^28^, amounting to USD 1200 billion in the period to 2030^22^. This exceeds pledges from developed countries to provide USD 100 billion annually to all developing economies from 2023. We therefore assess the CO_2_ emissions for the 2030 forecasts.

We provide our INVENTORY in unprecedented spatial resolution, with associated water use and CO_2_ emissions for each power plant. In general, sectoral water use data, including for electricity, are very scarce for African countries^29^. For many countries, data are even lacking on the national level. A persistent lack of reliable water data and information is a widespread constraint on water management in the global South^10^. For detailed water management and water scarcity assessments, accounting for different water demand stakeholders such as electricity, spatially detailed data are a necessity^23, 27, 30, 31^.

In addition, we provide all data open access for any stakeholder to use freely. This is of importance for researchers and stakeholders working in Africa, as they often lack the funds to purchase high-quality data and assessments. Some companies manage databases on existing power plants^32^; however, their data usage and replication are restricted and not available under a Creative Commons license^13^. This limits ongoing research efforts on the sustainable development of electricity production and constrains a science-based discussion among stakeholders in the decision process.

Our manuscript is organized in the following way: section “Results” provides results on the electricity production forecasts up to 2030, the water use forecasts up to 2030 and the CO_2_ emissions up to 2030. Section “Discussion” first generally discusses the results, followed by a discussion on policy implications and recommendations, followed by a brief discussion on limitations and uncertainties. Section “Methods” provides an overview of the methodology, presented in different steps and subsections.

In this study, we provide six forecasts for 2030 for the African continent on electricity production, with related water use and CO_2_ emissions. Our main assessment is INVENTORY, a detailed database on existing power plants and an estimate of under-construction and planned power plants. We also compute four forecasts based on historical electricity generation to analyse whether Africa is stepping up in power plant expansion. Additionally, we conduct a forecast based on the NDCs, to analyse whether current power plant construction as grasped by INVENTORY is in line with made commitments.

## Results

### Electricity production forecasts up to 2030

**Fig. 1:**
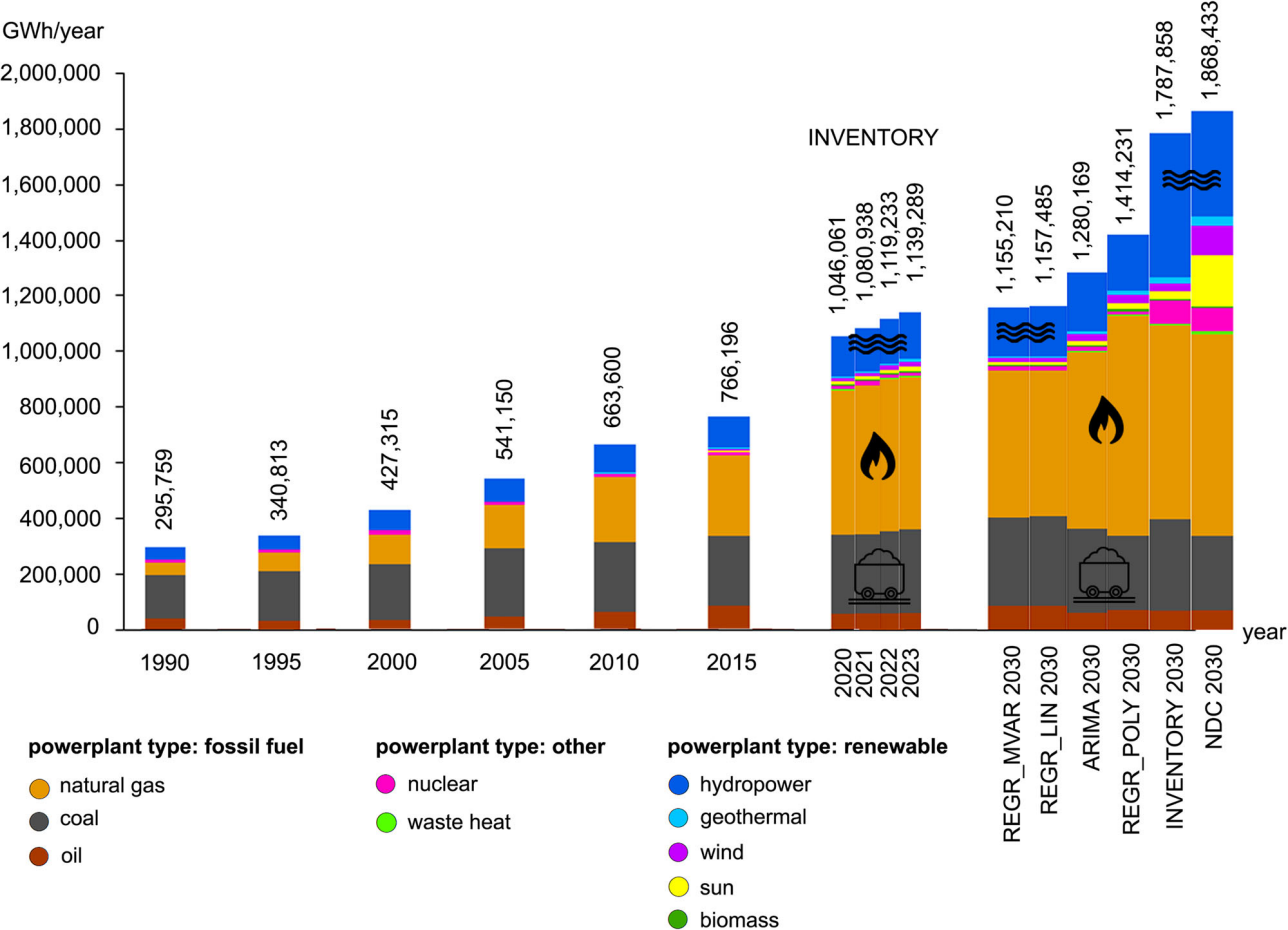
Electricity production (in GWh year^−1^) in Africa from 1990 to 2015 (historical), for INVENTORY (from 2020 to 2023 as well as 2030) and for the five remaining forecasts in 2030 (REGR_LIN, REGR_MVAR, REGR_POLY, ARIMA, NDC). Total amounts are shown as well as amounts per powerplant (fuel) type. Excluded from these amounts are Ascension Island, Comoros, Mayotte, Reunion, St. Helena, Swaziland, Tristan da Cunha, as they do not have individual nationally determined contributions (NDCs).

Africa-wide overall electricity production for INVENTORY has increased from 1,046,061 GigaWatt hours (GWh) in 2020 to 1,080,938 GWh in 2021 (+3%), 1,119,233 GWh in 2022 (+7%) and 1,139,289 GWh in 2023 (+9%) (Fig. 1). We forecast for INVENTORY an increase to 1,787,858 GWh in 2030 (+741,797 GWh or +71% relative to 2020; +648,569 GWh or +57% relative to 2023). Past electricity production evolution from 1990 up to 2023 forecasts lower amounts for 2030: 1,157,485 GWh for REGR_LIN, 1,155,210 GWh for REGR_MVAR, 1,280,169 GWh for ARIMA, and up to 1,414,231 GWh for REGR_POL. In comparison with the past evolution in electricity production, Africa is thus stepping up in planning and constructing additional power plants to increase power production for the period 2024–2030. Current power plant construction, however, still falls short on commitments in the NDCs, which amount to 1,868,433 GWh. Additional power plants should be constructed up to 2030 to meet these commitments.

The electricity production per power (fuel) type also differs between INVENTORY and NDC for 2030 (Fig. 1). For INVENTORY, of a total 1,787,858, 1,098,120 GWh (61%) is produced by fossil fuels, 599,220 GWh (34%) by renewable energy and 90,519 GWh (5%) by other sources. For NDC, of a total 1,868,433, 1,068,020 GWh (57%) is produced by fossil fuels, 709,352 GWh (38%) by renewable energy and 91,061 GWh (5%) by other sources. For NDC, more electricity is thus produced by renewable resources, both proportionally and in absolute amount. For the fossil fuels, considerably more power is produced from coal for INVENTORY (331,593 GWh) compared to NDC (269,647 GWh), whereas for natural gas it is the opposite (693,476 and 725,323 GWh, respectively). For the renewables, considerably more power is produced from hydropower in INVENTORY (517,501 GWh) compared to NDC (378,598 GWh). For solar, it is the opposite (24,570 and 179,645 GWh, respectively), as well as for wind (30,685 and 109,627 GWh, respectively).

**Fig. 2:**
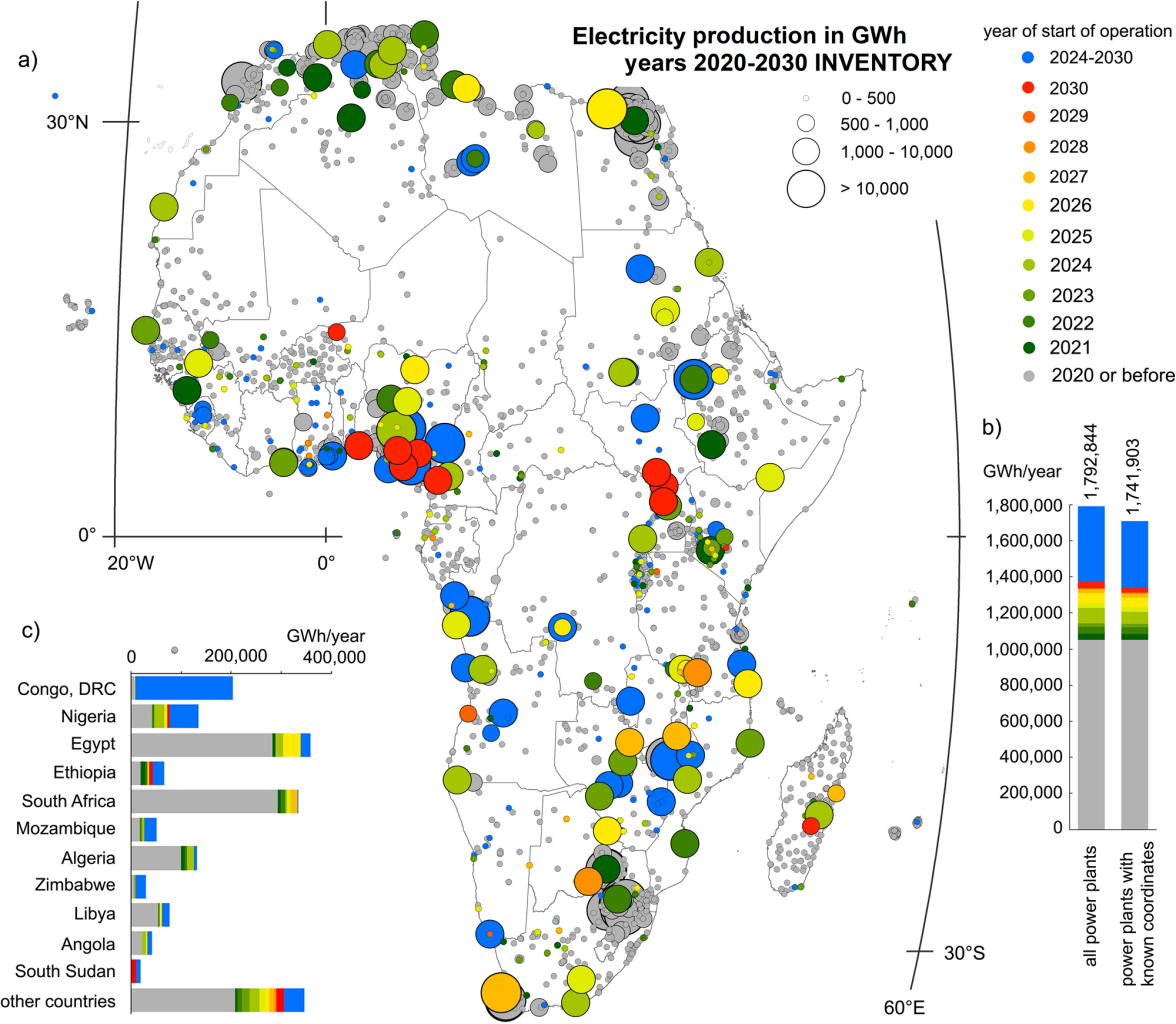
Total electricity production (in GWh year^−1^) for all power plants in INVENTORY, for each year from 2020 up to 2030 (including Ascension Island, Comoros, Mayotte, Reunion, St. Helena, Swaziland, Tristan da Cunha). **a** Map of power plants with known coordinates, with indication of power production amount (size of circle) as well as year of start of operation (colour of circle, blue indicates the period 2024–2030, but uncertain which year exactly). **b** Total electricity production amount according to year of start of operation for all powerplants (1,792,844 GWh, more than 1,787,858 GWh as presented before due to inclusion of islands) and powerplants with known coordinates (1,741,903 GWh). **c** National electricity production according to the year of start of operation, for the eleven countries with the highest amounts for the period 2021–2030. Map in a created with ArcGis using country/continent borders from GADM.

We identify in INVENTORY 605 additional power plants with a start of operation during the period 2021–2030 (with a cumulative power production of 742,170 GWh, adding up to 1,792,844 GWh operational in 2030, Fig. 2b), compared to the year 2020. Of these, 551 can be geolocated by means of *x* and *y* coordinates (with a cumulated energy production of 691,228 GWh, adding up to 1,741,903 GWh operational in 2030, Fig. 2b). Additional power plants have been or are being constructed, spread heterogeneously over Africa (Fig. 2a). For a large proportion of additional power plants, the actual year of start of operation is known. For many, the year of start of operation is known to be from 2024 to 2030, due to missing data on the actual year.

**Fig. 3:**
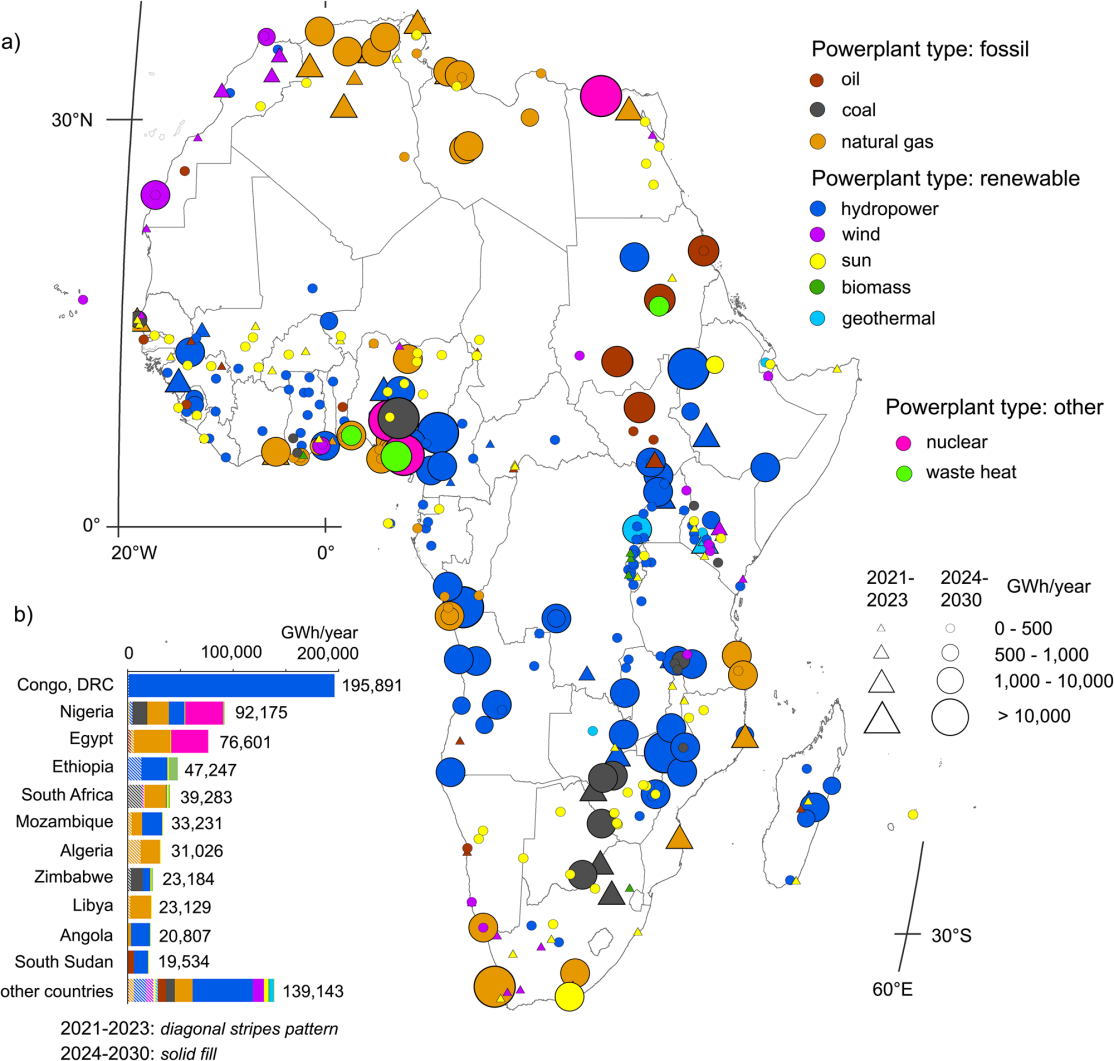
Total electricity production (in GWh year^−1^) for all power plants in INVENTORY, for each year from 2021 up to 2030, according to power plant (fuel) type. **a** Map of existing (triangle, period 2021–2023) and new (circle, period 2024–2030) power plants with known coordinates, with indication of power plant type (colour of triangle/circle) and power production amount (size of triangle/circle). **b** National energy production according to power plant type, for the eleven countries with the highest amounts for the period 2021–2030. Map in a created with ArcGis using country/continent borders from GADM.

In terms of additional power plants during the period 2021–2030 with the highest national cumulative power production, Congo (DRC) adds the most power production with 195,891 GWh, followed by Nigeria (92,175 GWh) and Egypt (76,601 GWh) (Figs. 2c and 3b). For the DRC, primarily hydropower plants are constructed (total 195,823 GWh), with the start of operation between 2024 and 2030, i.e., the Grand Inga Power Station (152,321 GWh) and the Inga III Power Station (40,075 GWh) on the downstream part of the Congo river, the largest dam project in the world (Fig. 3a and b). Nigeria invests in nuclear power by building the Geregu and Itu nuclear power plants (17,636 GWh each). It is building coal-fired (Itobe project 14,155 GWh) and natural-gas-fired (e.g. Sparkle Power Plant, 8848 GWh) power plants, as well as hydropower plants (e.g. Mambilla, 11,777 GWh). Cumulative solar and waste heat projects amount to 1159 and 1939 GWh, respectively. In Egypt, focus is put on the construction of a nuclear power plant (Dabaa power plant, 35,272 GWh) and additional gas-fired power plants (combined 37,410 GWh).

During the period 2021–2030, Ethiopia is increasing its power production by 47,247 GWh, of which 37,482 GWh is hydropower extension, 8434 GWh geothermal, 748 GWh solar and 582 GWh wind (Figs. 2 and 3). The value for South Africa is 39,283 GWh, of which a coal-fired plant extension of 13,810 GWh during 2021–2023 (such as the Kusile plant of 9227 GWh in 2022), as well as wind and solar extensions during the same period of 1656 and 256 GWh, respectively. From 2024 to 2030 it expands gas power plants by 20,884 GWh, such as the Coega power plant in the Eastern Cape (8017 GWh) in 2025. It expands solar power by 2508 GWh, such as by the Coega Solar PV Park (2221 GWh).

Other countries that substantially increase power production in absolute terms during the period 2021–2030, include Mozambique (33,231 GWh, of which 19,171 GWh hydropower and 13,918 GWh natural gas), Algeria (31,026 GWh, of which 30,859 GWh natural gas) and Zimbabwe (23,184 GWh, of which 14,302 GWh in coal-fired power plant expansion and 7318 GWh hydropower)(Fig. 3). For Libya, Angola and South Sudan the amounts are 23,129, 20,807 and 19,534 GWh, respectively, mostly in hydropower and natural gas expansion.

**Fig. 4:**
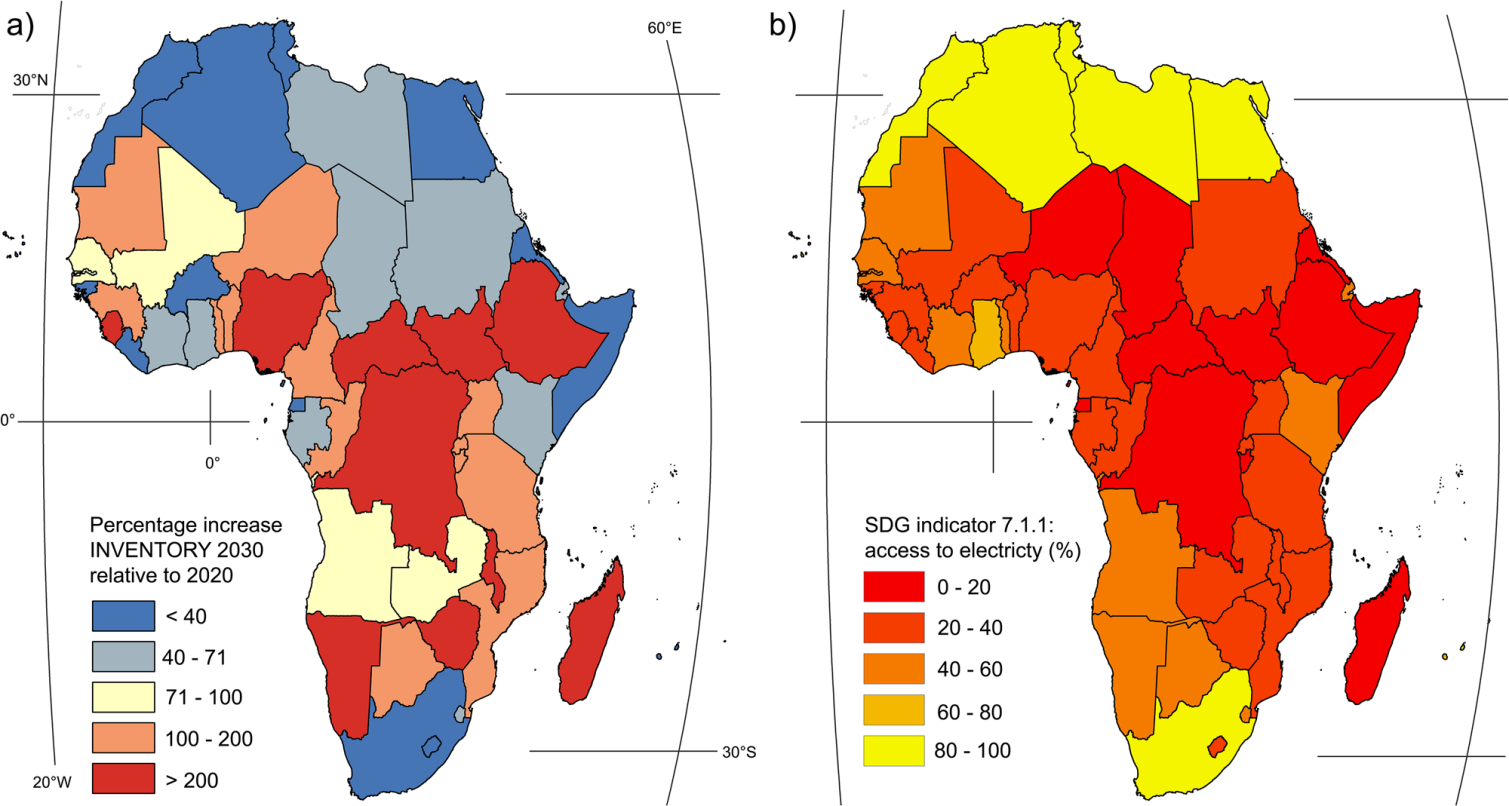
Key national indicators on power production and access to electricty. **a** Percentage (%) increase in national power production for INVENTORY in 2030 relative to 2020. The average amount for the African continent is 71%. **b** National access to electricity in percentage (%) for 2020–2022 (Sustainable Development Goal or SDG Indicator 7.1.1)^33^. Map in **a** and **b** created with ArcGis using country/continent borders from GADM.

While electricity production for INVENTORY is forecasted to increase by 71% for the entire African continent in 2030 compared to 2020, the relative change is for certain countries higher and others lower than this average amount (Fig. 4). Countries with a high relative increase, include South Sudan (+7443%), the DRC (+2941%), Namibia (+1905%), Sierra Leone (+371%), Zimbabwe (+328%), Madagascar (+253%), Ethiopia (+247%), Nigeria (+220%), Central African Republic (+213%), Malawi (+210%), Niger (+198%), Guinea (+196%), Angola (+96%) and many others. Countries with a less than average increase include large countries such as Sudan (+66%), Kenya (+66%), Libya (+43%), Algeria (+31%), Morocco (+27%), Egypt (+27%), South Africa (+13%) and Tunisia (+10%). Some of these countries are identified with a large absolute increase (Figs. 2 and 3, Egypt, South Africa, Algeria, Libya), yet their relative increase is smaller than average. Some countries currently have a larger access to electricity, especially in northern Africa and South Africa (Fig. 4b^33^), which could partly explain the lower relative increase. Yet, not only does the general population require electricity access, but also other sectors such as industries, mining or the transport sector, many of which are growing and would require additional electricity.

In certain countries, almost no additional power plants are planned or constructed, such as Somalia (+6%), although only 6% of its population currently has access to electricity. In others, no additional power plants are in the INVENTORY for 2030 (relative change 0%), such as Eritrea or Guinea-Bissau, countries with 12% and 29% of the population with electricity access, respectively. Due to unstable governments and institutions, there is currently no investment in constructing power plants for increasing electricity access.

### Water use forecasts up to 2030

**Fig. 5:**
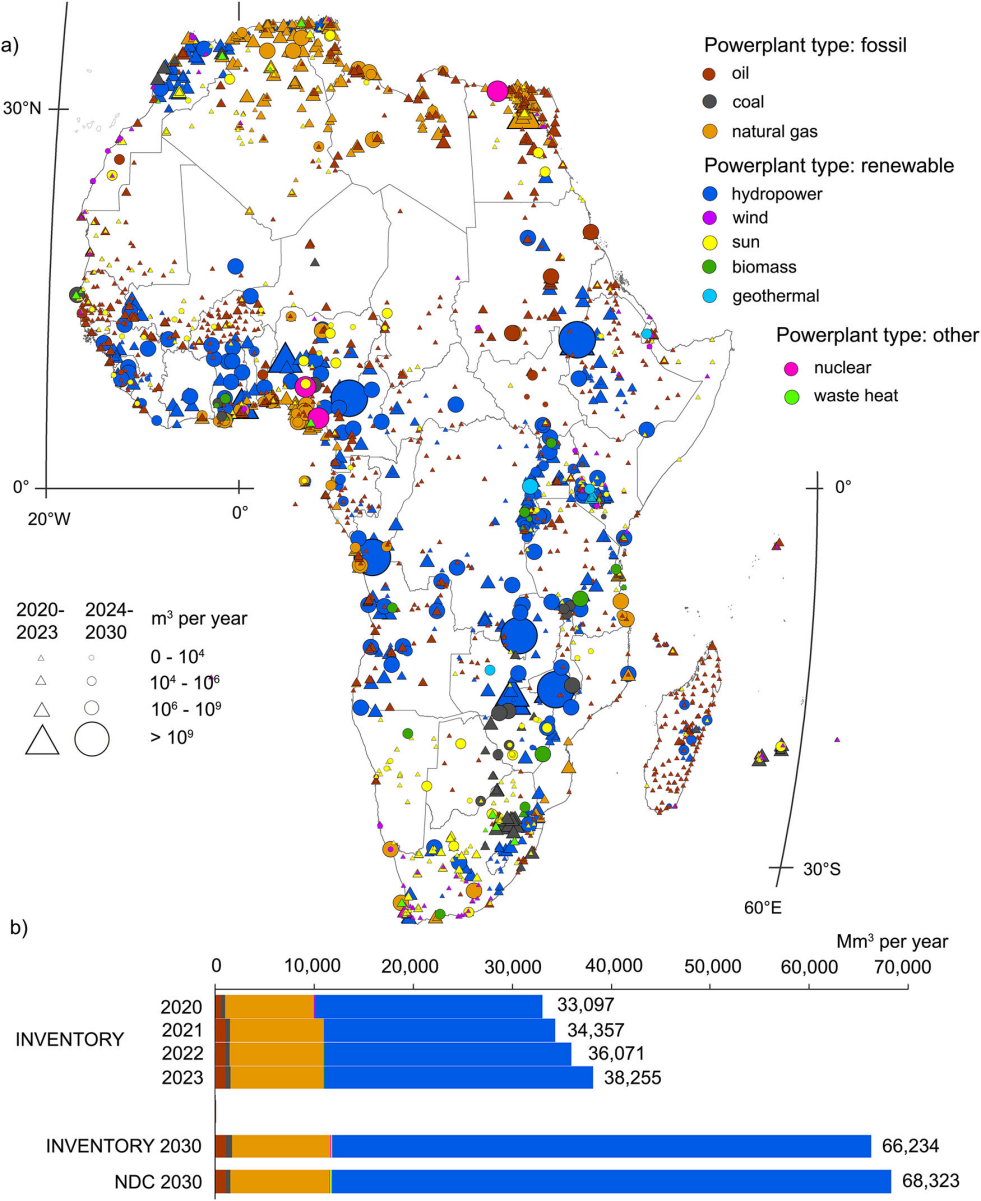
Total Water Withdrawal (WW) for all power plants in INVENTORY and NDC (nationally determined contributions), from 2020 up to 2023, as well as for 2030, according to power plant (fuel) type. **a** Map of existing (triangle, period 2020-2023) and new (circle, period 2024–2030) power plants with known coordinates for INVENTORY, with indication of power plant type (colour of triangle/circle) and WW amount in m^3^ year^−1^ (size of triangle/circle). **b** Total WW (Mm^3^  year^−1^) according to power plant type for INVENTORY for the years 2020–2023 and 2030, as well as for NDC 2030. Map in a created with ArcGis using country/continent borders from GADM.

Africa-wide, for INVENTORY, water withdrawal (WW) increased from 33,097 Mm^3^ in 2020 to 34,357 Mm^3^ in 2021, 36,071 Mm^3^ in 2022 and 38,255 Mm^3^ in 2023 (+16% relative to 2020)(Fig. 5b). In 2023, fossil fuels account for 29% of total WW, with especially natural gas accounting for a high amount (9462 Mm^3^ or 25% of total WW). Renewables account for 71% of total WW. Hydropower is the dominant WW user, as it accounts for 27,165 Mm^3^ or 71% of total WW.

For INVENTORY, we forecast a large WW increase to 66,234 Mm^3^ for 2030 (+100% and +73% relative to 2020 and 2023, respectively). Achieving the NDCs would result in a WW increase to 68,323 Mm^3^ in 2030 (+106% and +79% relative to 2020 and 2023, respectively). These increases are generally the result of natural gas and hydropower expansion up to 2030 (Fig. 5b). Especially, hydropower expansion results in a substantial WW increase. It increases from 27,165 Mm^3^ in 2023 to 54,469 and 56,546 Mm^3^ in 2030, for INVENTORY and NDC, respectively.

For INVENTORY, we forecast large increases in hydropower WW up to 2030 in countries such as the DRC, Ethiopia, Nigeria, Mozambique, Zambia, Cameroon, Ghana, Tanzania and Kenya (Fig. 5a). Large increases in natural gas WW up to 2030 are forecast for countries such as Egypt, South Africa, Nigeria, Algeria, Libya and Tanzania. For NDC, large increases in hydropower WW up to 2030 are forecast in countries such as Ethiopia, Nigeria, Mozambique, Zambia, Cameroon, Ghana, Tanzania and South Africa. Similar to INVENTORY, large increases in natural gas WW up to 2030 are forecast for countries such as Egypt, South Africa, Nigeria, Algeria, Libya and Tanzania.

**Fig. 6:**
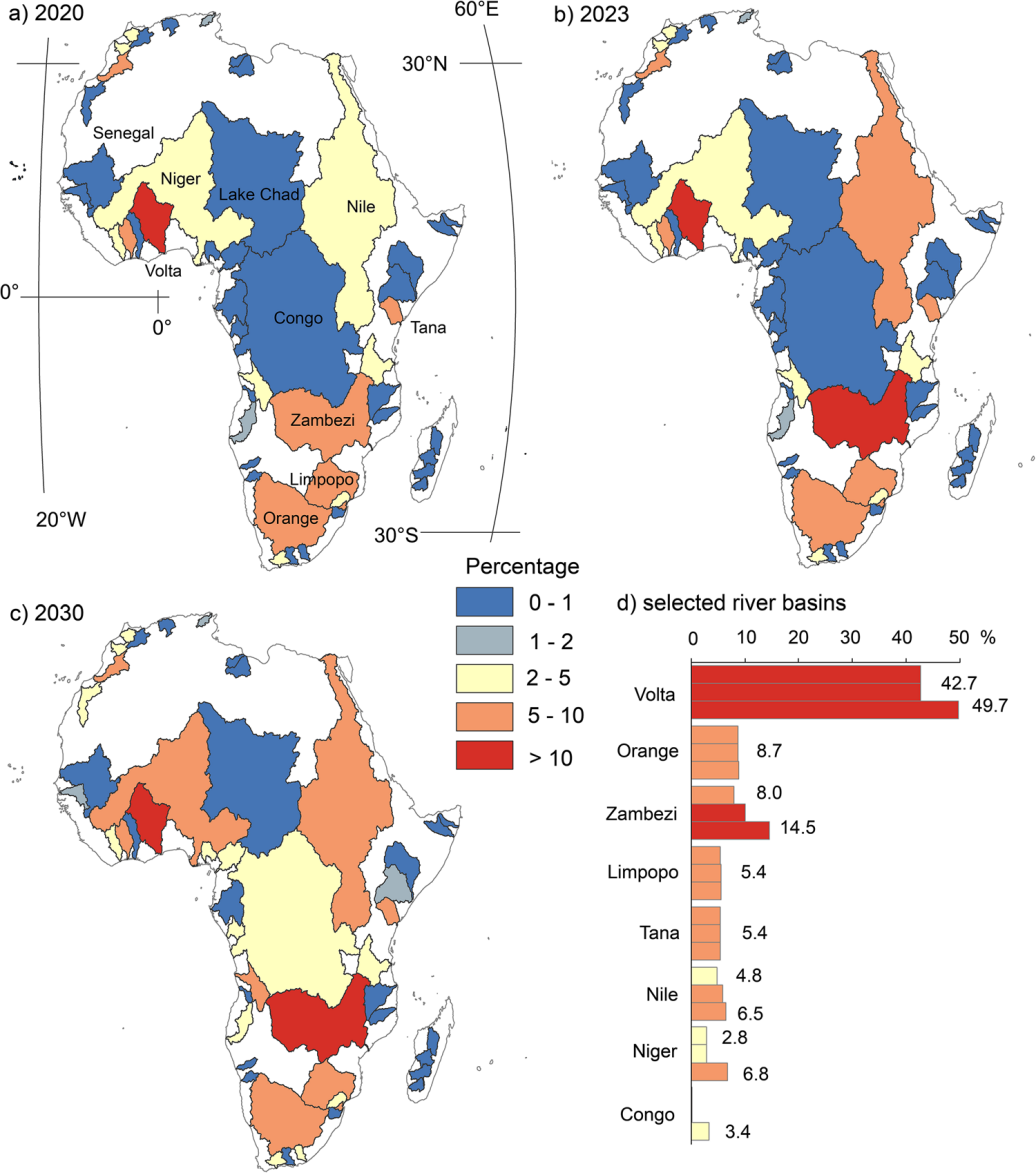
Water Withdrawal (WW) as a percentage of renewable water availability in major African river basins. Highlighting selected basins with the highest amounts, for INVENTORY for the years 2020 (**a**), 2023 (**b**) and 2030 (**c**). For 2030, only new power plants with known coordinates are included. **d** shows amounts for selected river basins (upper bar 2020, middle bar 2023, lower bar 2030). Map in **a** and **b** created with ArcGis using country/continent borders from GADM.

Increased WW for electricity production puts additional pressure on available renewable water resources in the main African river basins (Fig. 6). Available renewable water resources are defined as natural renewable water minus environmental flows (EFs), in this case the high level protection EFs as defined by Richter et al. ^34^. For many river basins, the relation between WW and water availability increases from 2020 to 2023 to the forecast of 2030. These include the Volta (from 42.7% in 2020 and 2023 to 49.7% in 2030), the Zambezi (from 8% in 2020 to 10.1% in 2023 to 14.5% in 2030), the Nile (from 4.8% in 2020 to 5.8% in 2023 to 6.5% in 2030), the Niger (from 2.8% in 2020 to 2.9% in 2023 to 6.8% in 2030) and the Congo (from 0.1% in 2020 and 2023 to 3.4% in 2030) river basins. This will increase competition for available water resources with other sectors, including the environment.

Using a low EF protection level reduces the amount in the relation between WW and water availability, but confirms that the relation between WW and water availability increases from 2020 to 2023, to the forecast of 2030 (Supplementary Fig. 2).

### CO_2_ emissions up to 2030

**Fig. 7:**
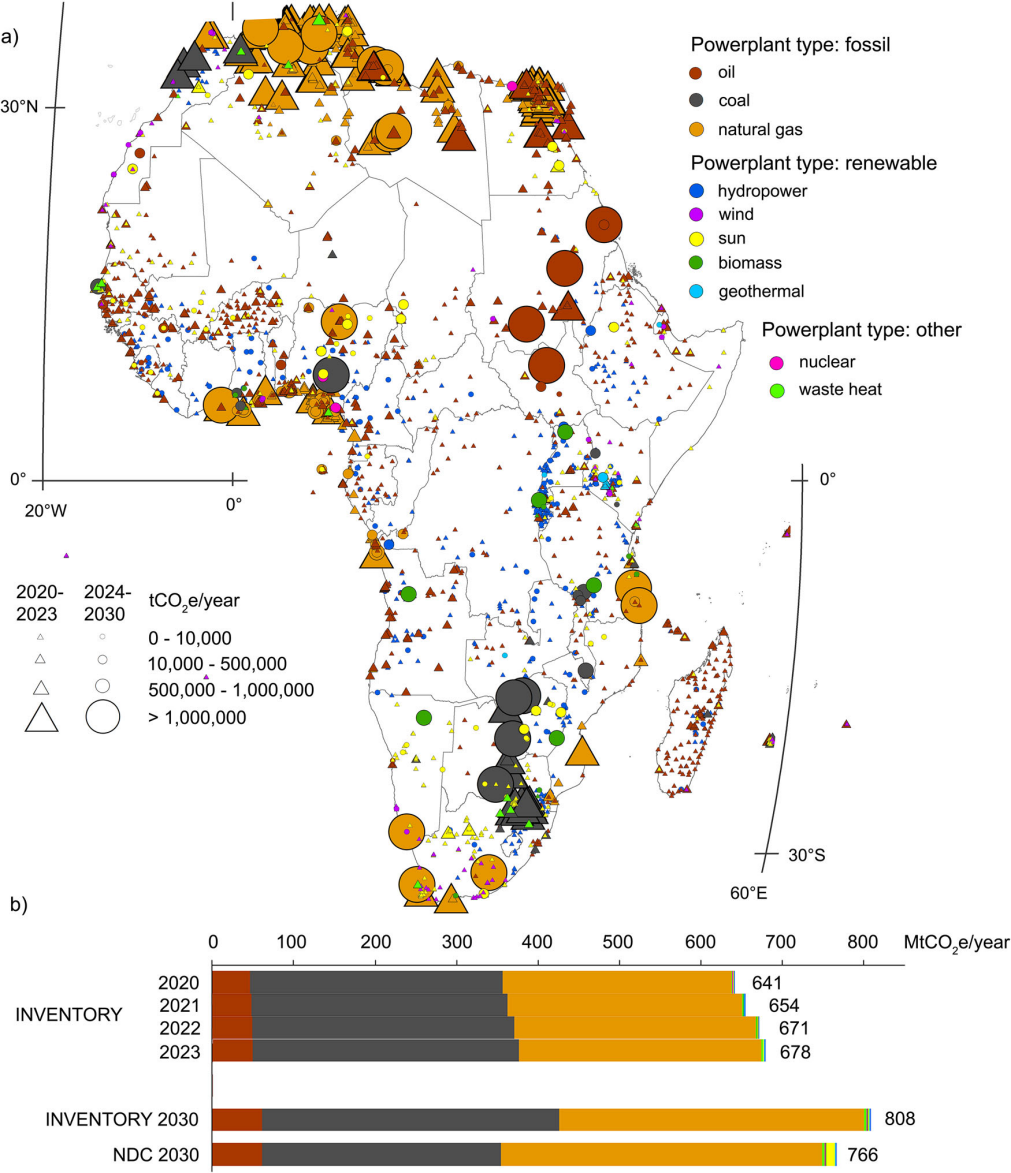
Total CO_2_ emissions (CO_2_e) for all power plants in INVENTORY and Nationally Determined Contributions (NDC), from 2020 up to 2023, as well as for 2030, according to power plant (fuel) type. **a** Map of existing (triangle, period 2020–2023) and new (circle, period 2024–2030) power plants with known coordinates for INVENTORY, with indication of power plant type (colour of triangle/circle) and CO_2_ emissions amount in tCO_2_e year^−1^ (size of triangle/circle). **b** Total CO_2_ emissions (MtCO_2_e year^−1^) according to power plant type for INVENTORY for the years 2020–2023 and 2030, as well as for NDC 2030. A map was created with ArcGIS using country/continent borders from GADM.

Africa-wide, for INVENTORY, CO_2_ emissions increased from 641 Mt CO_2_e in 2020 to 654 Mt CO_2_e in 2021, 671 Mt CO_2_e in 2022 and 678 Mt CO_2_e in 2023 (+6% relative to 2020) (Fig. 7b). In 2023, fossil fuels account for 99.5% of total CO_2_ emissions, i.e. oil for 50 Mt CO_2_e (7.5%), coal for 326 Mt CO_2_e (48%) and natural gas 298 for Mt CO_2_e (44%). Renewables account for the remaining 0.5% of CO_2_ emissions.

For both the INVENTORY, based on actual plans and construction, as well as for the NDCs, based on policy goals, CO_2_e emissions are forecast to increase up to 2030. The overall increase is smaller for NDC as compared to INVENTORY. Achieving the NDCs would result in a CO_2_ emissions increase to 766 CO_2_e in 2030 (+20% and +13% relative to 2020 and 2023, respectively). Emissions increase to 61 Mt CO_2_e for oil (identical to INVENTORY), to 394 Mt CO_2_e for natural gas (+32%), but decrease to 293 Mt CO_2_e for coal (−10%). For INVENTORY, emissions increase to 808 Mt CO_2_e for 2030 (+26% and +19% relative to 2020 and 2023, respectively). These relatively large increases are the result of fossil fuel expansion. Emissions increase to 61 Mt CO_2_e for oil (+23% relative to 2023), to 364 Mt CO_2_e for coal (+12%), and to 375 Mt CO_2_e for natural gas (+26%). For natural gas, there is thus a larger CO_2_e emissions increase for NDC compared to INVENTORY, due to a net expansion in natural gas. There is, however, a decrease in coal-related CO_2_e emissions in NDC compared to INVENTORY, due to a net decrease in coal-fired power production in NDC. In INVENTORY, some additional coal-fired power plants are constructed up to 2030, whereas in NDC, selected coal-fired power plants are shut down.

For INVENTORY, large increases in coal CO_2_e emissions up to 2030 are forecast in countries such as Nigeria, Zimbabwe and Botswana (Fig. 7a). Large increases in natural gas CO_2_e emissions up to 2030 are forecast for countries such as South Africa, Egypt, Mozambique, Nigeria, Algeria and Libya, Namibia, Cote d’Ivoire and Tanzania. For NDC, increases in coal and natural gas occur in the same countries, while in South Africa, selected coal-fired power plants are decommissioned (Grootvlei, Kelvin, Majumba, Matla, Sasol one, Tutuka, combined 71 Mt CO_2_e) and one natural gas power plant is added (19 Mt CO_2_e), compared to INVENTORY.

## Discussion

Here we show that Africa is stepping up in planning and constructing additional power plants up to 2030 to increase electricity access, by producing the most detailed spatial inventory of African power plants from 2020 until 2030 available. Our database covers 3139 individual plants, including information on fuel type (oil, coal, natural gas, hydropower, wind, sun, biomass, geothermal, nuclear and waste heat), location (coordinates), installed capacity (MW), electricity production (MWh), water withdrawal, water consumption, CO_2_ emissions and other information, such as climate, operating conditions and data sources. This level of detail, including future projections, goes well beyond existing spatially distributed databases, such as WRI’s GPPD^12^, RePP Africa^13^, Gonzalez Sanchez et al. ^14^ or Gerbens-Leenes et al. ^11^ (Supplementary Table 1).

We forecast an increase in electricity production to 1,787,858 GWh in 2030 (+57%) for INVENTORY, from 1,139,289 GWh in 2023. This is a higher amount than expected from past evolutions in electricity production from 1990 to 2015 (REGR_LIN, REGR_MVAR, ARIMA and REGR_POL). This confirms Africa is stepping up, with some countries driving the forecast, others lagging behind. Current additional power plant construction (INVENTORY) falls slightly short of the commitments in the NDCs, which amount to 1,868,433 GWh. Also, the electricity produced by power plant types differs between INVENTORY and NDCs for 2030. For NDC, more electricity is produced by renewable resources, both proportionally and in absolute amount. For the fossil fuels, considerably more power is produced from coal for INVENTORY compared to NDC, whereas for natural gas, it is the opposite. For the renewables, more power is produced from hydropower in INVENTORY compared to NDC. For solar and wind, it is the opposite.

**Fig. 8:**
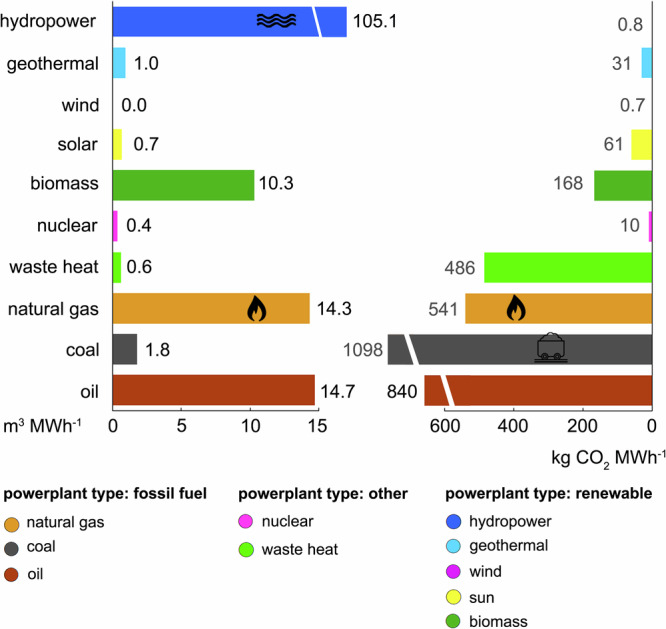
Average water and CO_2_ intensity (Water Withdrawal or WW in m^3^ MWh^−1^ and CO_2_ emissions in kg CO_2_ MWh^−1^) per power plant fuel for Africa. Note that these are average values for all power plants, whereas individual plants show a wide range. The bar charts for WW (left) and CO_2_ emissions (right) for each fuel type are displayed in the same colour.

Compared to the current WW and CO_2_e emissions of power production of 38,255 Mm^3^ and 678 Mt CO_2_e (year 2023), respectively, we forecast a substantial increase up to 2030 for both these environmental pressures^8^. For INVENTORY, we forecast an increase to 66,234 Mm^3^ and 808 Mton CO_2_e, whereas for NDC the amounts are 68,323 Mm^3^ and 766 Mton CO_2_e. The total amounts in current and forecast WW are dominated by hydropower (due to its high average water intensity of 105 m^3^ MWh^−1^, Fig. 8) and to a lesser extent by natural gas (average water intensity of 14.3 m^3^ MWh^−1^). For 2023, gas and hydropower account for 25% and 71% of total WW, respectively. For 2030, the values are 15% for gas and 82–83% (INVENTORY-NDC) for hydropower. There are important trade-offs in water and carbon intensity of different fuel types. The total amounts in current and forecast CO_2_e emissions are dominated by fossil fuels, due to their high average carbon intensities compared to renewables (Fig. 8). Coal has an average carbon intensity of 1098 kg CO_2_ MWh^−1^, whereas for oil and natural gas the amounts are 840 and 541 kg CO_2_ MWh^−1^, respectively. These amounts are very high compared to hydropower, with an average intensity of 0.8 kg CO_2_ MWh^−1^. For 2023, oil, coal, gas and renewables account for 7.5%, 48%, 44% and 0.5% of total CO_2_ emissions, respectively. For 2030, all fossil fuels account for 98–99% and hydropower <0.5% of total CO_2_ emissions.

These trade-offs are visible in the difference in total WW and CO_2_ emissions between INVENTORY—what is actually constructed and likely to be operational by 2030—and NDC—what is committed to. Higher CO_2_e emissions in INVENTORY are due to additional fossil fuel-based power plants (especially coal and gas-fired) being constructed, whereas in NDC, several coal power plants are decommissioned and replaced by less carbon-intensive gas-fired power plants. On the other hand, throughout different parts of Africa, carbon low hydropower^35^ has been developed and is still under development, leading to substantially increased total WW forecasts for 2030. These put additional pressure on available water resources in many major African river basins (Fig. 6).

Hydropower is one of the major causes of the decline in freshwater biodiversity^36, 37^. Almost one in three freshwater species is threatened with extinction, which is consistently higher than for their terrestrial counterparts^38–40^. Hydropower is also vulnerable to droughts and floods, aggravated by climate change^41–44^. The drought throughout 2024 in Southern Africa has plunged Zambia into daily blackouts, as the reservoir of the Kariba hydroelectric dam has not enough water^45^. We forecast that the Zambezi basin, in which Kariba is located, will experience additional WW pressure by 2030 (Fig. 6 and Supplementary Fig. 2). The large increase in hydropower development in Africa up to 2030 is good for low CO_2_ emissions, but has a high vulnerability to drought and might negatively impact freshwater biodiversity. The latter, which includes fish, amphibians, but also large animals such as crocodiles and hippos, is also important for local food security and income from wildlife tourism.

To increase the resilience of electricity production, a mix of different (renewable) energy sources is required, which includes not only hydropower, but also solar and wind energy^44, 46–48^. While our forecast also shows significant development of solar and wind energy by 2030, hydropower is dominant in future development, as it has been in the past^47^. Previously, solar and wind technologies were not at the high standard they are now and were expensive to construct, whereas hydropower was an established technology. Given the low water and carbon intensity of wind and solar, additional expansion of these low-cost energy sources can be considered win-win in many African settings, especially as Africa currently still falls short of achieving NDCs by 2030.

Carlino et al show that declining costs of solar and wind, as well as climate change, curb the need for African hydropower expansion^49^. According to Peters et al. ^47^, declining costs for solar photovoltaics (90% decline since 2009) and wind turbines (55–60% decline since 2010) mean solar and wind have the potential to lead sustainable renewable energy pathways in Africa going forward. Africa-wide, wind and solar potential are far less exploited than hydropower^47^. Due to the different mean construction times of hydropower plants (10 years for larger plants) on the one hand and solar projects and wind farms (1–2 years) on the other hand, we most probably underestimate new solar and wind capacity construction up to 2030 in INVENTORY. Given Africa’s increasing commitment to low-carbon development, increased solar and wind construction up to 2030 might even result in many African countries reaching their NDCs, given investment is guaranteed^47^. When additional capacity is installed, that would only strengthen our statement that, overall, Africa is stepping up in planning and constructing additional power plants. Nevertheless, individual countries still require a serious stepping up in the construction of renewable power plants.

Policies on the electricity system, therefore, need to account for different carbon emissions and water intensities of energy sources (Fig. 8) as well as local water availability (Fig. 6 and Supplementary Fig. 2) and the water demand of other sectors. This requires nexus thinking^7^, coordination and collaboration between different ministries (such as ministries of energy, environment, water, agriculture/irrigation, among others) and stakeholders, thereby aligning policies on energy, water, agriculture, industry and the environment^50^.

The Kenyan “Energy Transition & Investment Plan 2023–2050” by the Kenyan Ministry of Energy and Petroleum^51^, e.g., outlines the country’s path to achieving net-zero emissions by 2050. It aims at decarbonizing the energy system by increasing the construction of renewables (geothermal, hydropower, solar, wind) and ensuring flexibility (batteries, demand response). The plan recognizes that to successfully implement the net-zero ambition in the energy sector, a best practice governance structure, process, and action plan is required, involving transparency and coordination across the ministries and sectoral policies that are consistent with national objectives. In the plan^51^, the water demand of the energy system and its relation to local water resources is not explicitly mentioned, although Kanda et al. ^52^ confirm that Kenyan water policies should have a central position in Water-Energy-Food (WEF) nexus-related policies. We forecast (INVENTORY) an increase in electricity production from 12,006 to 19,880 GWh (+66%) for Kenya by 2030 compared to 2020. This leads to a 51% increase in water demand, from 275 to 416 Mm³, with hydropower contributing the largest share of this rise. Given that Kenya is already facing water stress, projections like ours should be central to a broader, integrated nexus approach and policy development.

Targeted policy recommendations can be summarized as follows. First, policies on the electricity system require nexus thinking. They need to account for different carbon emissions and water intensities of energy sources (Fig. 8) as well as local water availability and the water demand of other sectors. Hydropower is vulnerable to increased and intensified drought events due to climate change, requiring a mix of energy source investments. Additional expansion in the low-cost energy sources, solar and wind, is a win–win in many African settings. Additional stepping up of these sources until 2030 and beyond may reach the NDCs.

Second, coordination and collaboration between different ministries and stakeholders is required, thereby aligning policies on energy, water, agriculture, industry and the environment^50^. Third, it is essential that African countries secure investment/financing for their planned and under-construction power plants, so that they are not delayed or cancelled. Financing can occur through a mix of public and private funding, from the private sector^53^, multilateral institutions such as the World Bank or African Development Bank^54^, bilateral development agencies or others.

Fourth, integrated solutions are required, including the construction of a mix of different (renewable) energy sources^55^, battery storage^55^, the establishment of a reliable national grid including transnational electricity sharing (international power pools)^47^, as well as small-scale localized solutions^47^. Fifth, policies need to address cooperation and river management across borders^56^.

Sixth, policies should be data and science-based. Our INVENTORY, an openly accessible and location-specific dataset for the years 2020–2030, is fundamental for the development of an integrated sustainable renewable energy mix. Countries need to share high-quality data, including data about their power plants and sectoral water uses

Different approaches and frameworks exist in electricity supply and demand modelling and forecasting, with many review papers published on the matter^57–59^. Their inputs range from socio-economic drivers (GDP, population, market trends, among others), the effect of energy and climate policies, to the effect of external shocks and the engagement of stakeholders^59^. Regarding the latter, there is growing interest in, and demand for, energy modellers to integrate more diverse perspectives on possible and preferred futures into the modelling process. McGookin et al. ^59^, e.g., provide a framework that offers multiple entry points for modellers to incorporate participatory elements either throughout the process or in individual stages. Our assessment indirectly involves stakeholders through using the national NDC’s for each country, as these are developed by countries themselves.

We do not use sophisticated modelling approaches in our paper. The main output of this paper is our African power plant inventory (INVENTORY). These represent the real-world situation of main power plants in Africa, as power plants take a long time to construct and our forecast is only for a short time window until 2030. We are confident that no model until now has modelled this situation. Therefore, INVENTORY can function as a calibration tool or anticipated result for existing or future models. By refining input data and/or parameters in existing/future models, INVENTORY provides a real-world forecast up to 2030 for training or finetuning models on the African, national or subnational level.

Moreover, we use three regression models (REGR_LIN, REGR_POL, REGR_MVAR) and ARIMA, which justify the statement that Africa is stepping up in planning and constructing additional power plants. The use of simple regression models to forecast electricity production for the short time frame 2024–2030 is justified by the specific objective of our analysis: to compare electricity production from INVENTORY with historical trends, rather than to develop highly accurate or optimized forecasts through modelling. We thereby place real government plans, including ongoing construction of power plants—already shaped by cost, policy, technical considerations and the inclusion of the private sector—within a context of historical electricity production. Simple regression models, as the ones we use, are well-suited for this purpose. This allows for an effective evaluation of whether planned and under-construction power plants align with, exceed, or fall short of those historical patterns.

Like any future forecasting study, our analysis has certain limitations and uncertainties, as discussed below.

The INVENTORY forecast assumes that all planned and under-construction power plants will be completed by 2030. This assumption is made based on the relatively short time window until 2030 and the average planning and construction time for power plants. On the one hand, this assumption may not account for certain delays, cancellations or changes in energy policies. It is, however, impossible to foresee such events or certain shocks (such as conflict, natural disaster or financial shock) that could lead to such events. On the other hand, extra power plants with relatively short planning and construction time (low-cost options, such as solar power plants and wind farms) could be constructed until 2030, for which currently no information is available.

Assuming in INVENTORY delays of 1 year for solar power plants and wind farms to become operational, for all other power plants 2 years, and accounting for potential delays in nuclear and large hydropower plants, decreases the electricity production forecast from 1,787,858 GWh in 2030 to 1,504,848 GWh. Much of this decrease is due to the assumption that the large Congo river hydropower plants are delayed, i.e., the Grand Inga Power Station (152,321 GWh) and Inga III (40,075 GWh). Other large hydropower plants include the Mambilla power plant in Nigeria (11,777 GWh) and Mphanda Nkuwa in Mozambique (10,710 GWh). We also assume the two new Nigerian nuclear power plants to be delayed (17,636 GWh). This much delayed version of INVENTORY thus only adds up to 1,504,848 GWh (−16% compared to 1,787,858 GWh), an amount still higher than the highest time series regression analysis (REGR_POL 2030, 1,414,231 GWh, Fig. 1). The delay of only these hydropower plants results in a reduction of 14%. The delay of only these nuclear power plants results in a reduction of 4%. Even for all these delayed versions of INVENTORY, it can be confirmed that Africa is stepping up in electricity production. As we most probably underestimate solar power plant and wind farm projects in INVENTORY for the later years up to 2030, our statement that Africa is stepping up remains valid.

INVENTORY is therefore to be regarded as an estimate, based on current information available.

As our paper was first submitted in November 2024, we present our analysis up to the year 2023 as existing data, and all successive years (2024–2030) as forecasted data. In 2026, we were able to identify the additional power plants becoming operational in the years 2024 and 2025 based on real-world data. We compare the results in Supplementary Table 8. In terms of total electricity produced, we compute in INVENTORY 1222 GWh for 2024 and 1251 GWh for 2025, whereas real-world data are 1161 GWh for 2024 and 1220 GWh for 2025 (difference of 5% and 2%, respectively). These are relatively low differences, generally due to delays in power plants becoming operational or new power plants becoming operational, not captured in INVENTORY. The latter is especially the case for power plants with short construction times, such as solar and wind, as discussed before. The same can be observed for the number of power plants, where the differences are 0% and −1% (Supplementary Table 8).

The regression forecasting methods (REGR_LIN, REGR_POL, REGR_MVAR) and ARIMA are based on historical trends, which may not accurately reflect future developments, especially in rapidly changing energy landscapes. We only used these regressions and ARIMA to evaluate the extent to which they forecast the amounts provided by INVENTORY by 2030. All three regressions and ARIMA underestimate total African electricity production as captured in INVENTORY by 2030 (Fig. 1), which justifies our statement that “Africa is stepping up in planning and constructing additional power plants”. This statement is justified as an overall Africa-wide observation, whereas national and fuel-specific observations can differ.

Supplementary Fig. 3 shows that natural gas-fired electricity production in Egypt, as quantified by INVENTORY for 2030 (290,810 GWh), is best forecast with ARIMA (304,279 GWh). REGR_LIN (Eq. (3)) and REGR_MVAR (Eq. (5)), which result in 247,599 and 256,742 GWh, respectively, underestimate the INVENTORY amount. REGR_POL (Eq. (4)) overestimates with 332,388 GWh the INVENTORY amount.

For multiple fuel types in different countries, REGR_POL forecasts a value close to the year 2030 INVENTORY amount, whereas REGR_LIN, REGR_MVAR and ARIMA underestimate this INVENTORY amount (Supplementary Fig. 4). This is the case for gas-fired electricity production in Libya (Supplementary Fig. 4a), where REGR_POL forecasts 64,856 GWh and INVENTORY 67,591 GWh, whereas REGR_MVAR forecasts 39,965 GWh, REGR_LIN 40,796 GWh and ARIMA 47,820 GWh. Other examples include solar PV in the RSA (REGR_POL 7107 GWh and INVENTORY 5673 GWh, Supplementary Fig. 4b), natural gas in Tanzania (REGR_POL 9155 GWh and INVENTORY 8346 GWh, Supplementary Fig. 4c) or solar PV in Namibia (REGR_POL 711 GWh and INVENTORY 608 GWh, Supplementary Fig. 4d). This clearly shows that the use of REGR_POL is justified for our short-term forecasting.

In many cases, even REGR_POL underestimates the INVENTORY electricity production amount for 2030 (Supplementary Fig. 5). This is the case for gas-fired electricity production in Nigeria, where REGR_POL forecasts 49,610 GWh, yet the INVENTORY amount is with 53,618 GWh substantially higher (Supplementary Fig. 5a). Other examples include solar PV in Kenya (REGR_POL 651 GWh and INVENTORY 994 GWh Supplementary Fig. 5b) and hydropower in Ethiopia (REGR_POL 39,936 GWh and INVENTORY 55,759 GWh Supplementary Fig. 5c). For geothermal in Tanzania, regression analysis forecasts 0 GWh for the year 2030, as geothermal has not been installed until 2023. The new investment in geothermal, as captured in INVENTORY with 1349, is thus not captured in regression forecasts nor ARIMA. Such examples show why the different regression analyses, as well as ARIMA, underestimate the combined INVENTORY electricity production amount by 2030 (Fig. 1).

The study does not account for potential improvements in power plant efficiency or new technologies that could emerge by 2030. This could affect both electricity production estimates and associated water use and CO_2_ emissions. When new thermal power plants are built, the efficiency might increase. However, based on the limited timeframe of the projection (2024–2030), we assume there is no significant change in efficiency. Moreover, power plants have a long lifetime, and the fraction of new thermal installations is small, so differences on a national scale will also be small. Electricity production expansion is mostly done using renewables like hydropower, solar and wind.

## Methods

This study makes an analysis of electricity production and related freshwater withdrawal, freshwater consumption and CO_2_ emissions in Africa for the period 2020–2030. Our analysis partly builds upon the study Gerbens-Leenes et al. ^11^, who recently created the spatially most detailed database on electricity production and related water demand for the year 2020 for Africa, including 2534 individual power plants with their specific location.

We conduct our study in five steps. In Step 1, we update the database for the year 2020 until the year 2023 in annual steps. In step 2, we make an estimate or forecast of electricity generation in 2030 for INVENTORY, which is based on information we found on decommissioned, projected and power plants under construction per country. In step 3, we make four estimates or forecasts of electricity generation in 2030 based on the time trends of electricity generation per country from the IEA^1^ and IRENA^22^ from 1990 to 2019, as well as on the database of 2020 and its update for 2023. They are extrapolations to 2030 by means of three different regressions, a linear one (called REGR_LIN), a polynomial one (called REGR_POL) and a multivariate regression using population and GDP as variables (called REGR_MVAR). Additionally, we make an extrapolation based upon an ARIMA (Autoregressive Integrated Moving Average) analysis.

In step 4, we identified the electricity projections per country based on the forecast of national policies as expressed in the Nationally Determined Contributions (NDCs), based on the Paris agreement^21^. This fifth forecast is called NDC.

Finally, in step 5 we estimated water withdrawal (WW), water consumption (WC) and CO_2_ emissions based on electricity generation per technology for INVENTORY and the three different regression based forecasts for 2030. These five steps are described below, and visualized in Supplementary Fig. 1. An overview of the different forecasts is provided in Table 1 and an overview of data sources is provided in Table 2.

**Table 1:** Overview of six different forecasts

Forecast	Description
INVENTORY	Spatial inventory of African power plants from 2020 until 2030. It is the result of an intensive, time-consuming data mining effort of open-access data sources. It is not the result of modelling. It provides a realistic analysis of existing power plants and an estimate of power plants under construction
Regressions and ARIMA	Extrapolations to 2030 by means of three different regressions, based on the time trends of electricity generation per country from the IEA^1^ and IRENA^23^ from 1990 to 2019 as well as on the database of 2020 and its update for 2023
REGR_LIN	Linear regression
REGR_POL	Polynomial regression
REGR_MVAR	Multivariate regression using population and GDP as variables
ARIMA	Autoregressive Integrated Moving Average
NDC	Electricity projections per country based on the forecast of national policies as expressed in the Nationally Determined Contributions (NDC’s), based upon the Paris agreement

**Table 2:** General overview of different data sources

Data	Sources
Electricity production per technology, for the different years in our assessment, as well as for the NDC	Supplementary Table 2
Power plants and characteristics in the year 2020	Gerbens-Leenes et al. ^11^
List of data sources for including the power plants that started operations between 2021 and 2023, per country	Supplementary Table 3
List of data sources to identify the power plants for the period 2024–2030, per country	Supplementary Table 4
Water withdrawal intensities for each power plant technology included in our assessment	Supplementary Table 5
Water consumption intensities for each power plant technology included in our assessment	Supplementary Table 6
Carbon Emission intensities for each power plant technology included in our assessment	Supplementary Table 7
Natural renewable water in high spatial resolution (0.1 degrees or 11.1 km at the equator)	Vanham et al. ^65^.

### Step one: Construction database 2023

For the construction of the database for 2023, we apply the method described in Gerbens-Leenes et al. ^11^. We identify new power plants per fuel type, installed capacity, electricity generation, fresh or saline water use, and location using publicly available open access data sources, to be able to provide our resulting database open access. Data sources include GEM wiki^17^ and Wikipedia^18^, because they provide recent information on power plants, especially on the large ones. Other data sources used were Power Technology^19^, which also provides information on installed capacity and year of commission. We also used Open Street maps, reports from national ministries or international organizations (e.g., the World Bank), scientific papers, companies’ websites, reports and newspapers, which give information on the opening or closure of specific plants.

We studied satellite images to confirm the coordinates of all power plants. We also used these satellite images for all thermal power plants to assess the cooling type, i.e., cooling tower, once-through or dry cooling, and type of water, i.e., freshwater or saline water.

As an example for 2023, we first assess the installed capacities in 2023 for country c, fuel f and technology *t*, IC_2023,c,f,t_ (MW), as Eq. (1):

1 $${{\rm{IC}}}_{2023,{\rm{c}},{\rm{f}},{\rm{t}}}={{\rm{IC}}}_{2020,{\rm{c}},{\rm{f}},{\rm{t}}}+{{\rm{IC}}}_{{\rm{new}},{\rm{c}},{\rm{f}},{\rm{t}}}-{I}_{{\rm{Cold}},{\rm{c}},{\rm{f}},{\rm{t}}}$$

where IC_2020,c,f,t_ are the installed capacities in 2020 for country c, fuel f and technology t, IC_new,c,f,t_ the newly installed capacities and IC_old,c,f,t_ the decommissioned installed capacities from 2020 to 2023 for country c, fuel f and technology t.

Next, we estimate electricity generation for country c, fuel f and technology t in 2023, *E*_2023,c,f,t_ (MWh), as Eq. (2):

2 $${E}_{2023,{\rm{c}},{\rm{f}},{\rm{t}}}={{\rm{IC}}}_{2023,{\rm{c}},{\rm{f}},{\rm{t}}}\ast {{\rm{SE}}}_{2023,{\rm{c}},{\rm{f}},{\rm{t}}}$$

where SE_2023,c,f,t_ is the specific electricity generation per unit of installed capacity for country c, fuel f and technology t (MWh MW^−1^). We derived data on specific electricity generation from Gerbens-Leenes et al. ^11^. As such, new power plants are added to the database of Gerbens-Leenes et al. ^11^ indicating their year of first being operational (2021, 2022, 2023).

### Step 2: Estimation of electricity generation in 2030 for INVENTORY

In step 2, we estimate electricity generation in 2030 per country for INVENTORY. The study first estimates the potential installed capacities of operational power plants in 2030. This is made by assessing publicly available data sources about the expansion in electricity generation for every African country. We included in the INVENTORY all the power plants that are currently under construction, or are being planned to start operations by 2030. Then we use Eq. (1) for the period 2020–2030. Next, we apply Eq. (2) to arrive at electricity generation per country, assuming that the specific electricity generation per unit of installed capacity does not change during this period.

### Step 3: Estimating electricity generation in 2030 by regression analysis

In step 3, we estimate electricity generation in 2030 per country for four forecasts: REGR_LIN, REGR_POL, REGR_MVAR and ARIMA.

We make an estimate of electricity generation in 2030 based on the growth rates between 1990 and 2020, by using regression analysis. We made an inventory of electricity generation per country for the period 1990–2020 in annual time steps. The IEA^1^ provides data for the largest African countries covering the period 1990–2020. However, data for some smaller countries were lacking. For those countries, we adopted data from IRENA^22^, which often provides data for a shorter time range. These countries include Burkina Faso, Gambia, Guinea, Liberia, Mali, Mauretania, Mauritius, Niger, Reunion, Seychelles, Sierra Leone, Somalia, South Sudan and Togo. For Acension Island, Chad, Djibouti, Equatorial Guinea, Guinea Bissau, Lesotho, Malawi, Mayotte, St Helena, Swaziland, Tristan da Cunha, Western Sahara and Zimbabwe no historical data were available. For those countries, we used our own estimations for 2020 and 2023 from the inventory for 2020^11^ and 2023 (step 1).

Using these time trends, we estimate electricity generation for country c, fuel f and technology in 2030 with linear (REGR_LIN), EL_2030,c,f,t_ (MWh), as Eq. (3):

3 $${{\rm{EL}}}_{2030,{\rm{c}},{\rm{f}},{\rm{t}}}=a\ast {\rm{YEAR}}+b$$

where *a* is the slope of the regression line calculated for the relation between the years (independent variable) and the electricity generation (dependent variable), and *b* is the intercept value, i.e. the value of the dependent variable (electricity production) when the independent variable (year) is zero

The same process is made for the estimation of electricity generation using the polynomial regression of the second order (REGR_POL), EP_2030,c,f,t_ (MWh), as Eq. (4):

4 $${{\rm{EP}}}_{2030,{\rm{c}},{\rm{f}},{\rm{t}}}=c*{\rm{YEA}}{{\rm{R}}}^{2}+{d}^{*}{\rm{YEAR}}+e$$

where *c*, *d* and *e* are constants calculated through iteration to minimize the sum of squared errors.

A polynomial regression of the second order and not higher orders is used. For many countries, the polynomial regression of the second order fits data points very well up to the year 2030, which is a short period for forecasting. As in African countries, generally, electricity production does not meet demand by 2030, a flattening of the curve as possible with a polynomial regression of the third order is not necessary.

We also conduct a multivariate regression (REGR_MVAR), EMV_2030,c,f,t_ (MWh), based on the variables population and GDP of the countries during the same years for which we have the electricity generation data, as Eq. (5):

5 $${{\rm{EMV}}}_{2030,{\rm{c}},{\rm{f}},{\rm{t}}}=r*{\rm{GDP}}+{s}^{*}{\rm{Population}}+t$$

where *r* is the constant defined as the contribution of GDP to electricity generation*, s* is the constant defined as the contribution of Population to electricity generation, and *t* is the intercept of the curve, calculated through iteration to minimize the sum of squared errors.

*Method for ARIMA*:

Time-series forecasting from 2023 until 2030 was also performed using the Autoregressive Integrated Moving Average (ARIMA) model, denoted as ARIMA (*p,d,q*), as used in many articles to forecast both, energy production and demand, i.e. Luzia et al. ^60^, Prakash et al. ^61^ and Suo et al. ^62^. The model used is as Eq. (6):

6 $$\left(1-\mathop{\sum }\limits_{i=1}^{p}{{{\varnothing }}}_{i}{B}^{i}\right){\left(1-B\right)}^{d}{y}_{t}=\left(1-\mathop{\sum }\limits_{j=1}^{q}{\theta }_{j}{B}^{j}\right){\eta }_{t}$$

where ${y}_{t}$ is the observed value at time $t$, $B$ is the backshift operator, represents the white noise, and $\phi$, and $\theta$ are model parameters. The latter were estimated using likelihood optimization with *statsmodels* Python library^63^.

The ARIMA model considers the autoregressive (AR($p$)), differencing of order $d$ and the moving average (MA($q$)) components. Thus, the first step is to identify the order of the model in terms of $p,{d}$ and $q$.

For that, and for each time series analysed, we performed the following steps: (1) Define the stationarity of the data to establish $d$, with (a) augmented Dickey–Fuller (ADF) test (H0: non-stationary) and (b) KPSS test (H0: stationary); and (2) to determine $p$ and $q$, we examined: (a) autocorrelation function (ACF) → identification of MA($q$) and (b) partial autocorrelation function (PACF) → identification of AR($p$).

Across countries and technologies, the differenced series consistently showed: (1) ADF and KPSS: Failed ADF while passing KPSS after first differences, validating $d=1$; (2) ACF: strong lag-1 spike and rapid decay, suggesting $q=1$; (3) PACF: strong lag-1 spike and rapid decay, suggesting $p=1$.

Thus, the canonical model ARIMA(1,1,1) was adopted uniformly to ensure comparability across all datasets. For that, we used the *fit ARIMA* function in the *statsmodels* Python library^64^.

To estimate the ARIMA for the technologies, an automated batch-processing script was used to ensure that each dataset—regardless of country, technology, or data format—was processed identically and reproducibly. The script is added to the paper as presented in the section “Code availability”.

Finally, to validate the data and error checking, several quality-control steps were applied before and after the model fitting as: (1) Frequency standardization of the datasets, in which we enforce annual start-of-year (YS) timestamps to avoid irregular spacing; (2) Minimum-sample check of the datasets, in which the ones with fewer than 10 valid observations were excluded, as ARIMA estimation becomes unstable with fewer points; (3) Review of possible ARIMA optimization failures; (4) Analysis of AIC values to define suitability of the results obtained.

### Step 4: Identification of electricity mix NDC’s

In accordance with Article 4, paragraph 12 of the Paris Agreement, NDCs communicated by parties are recorded in a public registry maintained by the secretariat (UNFCCC, 2024). For most countries, we used the indicated values for nominal capacity to be installed by country c, and fuel f. Because NDCs do not always provide information regarding technology, we assumed that each country c, will use the most available technology t for their NDC expansion.

Often, the NDCs do not provide information on electricity supply or electricity mixes, and only indicate goals for installed capacities or the fraction of renewables. This was the case for Burkina Faso, Ethiopia, Kenya, Madagascar, Mauritius, Sierra Leone and Zambia. With the exception of Kenya, in these cases, we considered that the NDC is the same as our INVENTORY. In the case of Kenya, we obtained the prospective electricity mix from a report by the Kenyan Ministry of Energy and Petroleum^51^.

For the estimates of electricity generation in 2030, we assumed that the electricity mix based on our inventory is also valid for the NDCs. Based on our inventory, we identified the electricity mix for country c, fuel f and technology t for 2030 and calculated electricity generation, *E*_2030,c,f,t_ (MW), using Eq. (2).

### Step 5: Water withdrawal, water consumption and CO_2_ emissions electricity 2020–2030

Step 5 calculates water withdrawal, water consumption and CO_2_ emissions related to electricity production per country for the period 2020–2030. For the calculation of freshwater withdrawal and consumption, we only include the operational stage, excluding water in the supply chain. Freshwater consumption per country n per energy source s*,*Water_n,s_ (m^3^ yr^−1^) was calculated as Eq. (7):

7 $${{\rm{Water}}}_{{\rm{n}},{\rm{s}}}=\,{E}_{{\rm{n}},{\rm{s}}}\ast {W}_{{\rm{s}},{\rm{o}},{\rm{c}}}$$

in which *E*_n,s_ is the electricity generation of energy source s (MWh yr^−1^) in country n and *W*_s,o,c_ is the specific freshwater consumption of energy source s, operational characteristic o (operation cycle and infrastructure) in climate c (m^3^/MWh). Freshwater withdrawal and CO_2_ emissions were calculated in the same way using the specific freshwater withdrawal data of energy source s in climate c using the same methodology as the one used in Gerbens-Leenes et al. (2024) and CO_2_ emissions from the National Renewable Energy Laboratory (NREL), 2024. For this calculation, differentiated factors for each power plant, depending on the climate and type, were used.

The water intensities are based on the following climate zones, as obtained from Kottek et al. ^64^: Tropical Rainforest (Af), Tropical Monsoon (Am), Tropical Savannah (Aw), Arid Steppe Hot (BSh), Desert Hot (BWh), Humid subtropical (Cfa), Temperate Without dry Season (Cfb), hot-summer Mediterranean (Csa), warm-summer Mediterranean (Csb), Dry-winter humid subtropical (Cwa), Dry-winter subtropical highland (Cwb).

For hydropower, we calculated water evaporation per plant depending on the reservoir surface areas, climate or other infrastructure.

All relevant information and data are included in Supplementary Tables 4–6 in the SI.

Next, we calculated freshwater consumption per country n (Water_*,*n_, m^3^ yr^−1^) as Eq. (8):

8 $${{Water}}_{{\rm{n}}}=\displaystyle \mathop{\sum }\nolimits_{s=1}^{t}{{Water}}_{{\rm{n}},{\rm{s}}}$$

Freshwater withdrawal and CO_2_ emissions per country n were calculated in the same way.

### Calculation of water demand as a percentage of renewable water availability for major African basins for INVENTORY

We quantified the relation of the water demand for electricity to renewable water availability in major river basins of Africa. We defined renewable water availability as natural renewable water minus environmental flows (EFs), as in Eq. (9):

9 $${\rm{renewable}}\,{\rm{water}}\,{\rm{availability}}={\rm{natural}}\,{\rm{renewable}}\,{\rm{water}}{\textstyle \mathrm{--}}{\rm{EF}}$$

Natural renewable water in high spatial resolution (0.1° or 11.1 km at the equator) was taken from Vanham et al. ^65^, who used the hydrological model LISFLOOD^66^. The model works at a daily time step for the period 1980–2018 and generates natural water availability as the sum of renewable surface and groundwater. We used the geodataset on river (sub)basins of Hydrosheds^67^ to aggregate grid natural renewable water amounts to the basin level.

EFs are the quantity and timing of water flows required to maintain the components, functions, processes and resilience of aquatic ecosystems and the goods and services they provide to people. They are required to maintain ecosystem integrity in streams, rivers, wetlands, riparian zones and estuaries. EFs also provide many additional ecosystem services, with direct links to specific SDGs^27, 68^.

To quantify EFs, we used the presumptive standard for EFs by Richter et al. ^34^, which defines 80% of the natural flow as EF. The remaining 20% is considered as water available for human use, in this paper defined as renewable water availability. The methodology by Richter is widely used in water management studies^23, 30, 69–72^. This presumptive standard is supported by empirical studies showing that flow alterations within 20% support native fish species and flow alteration beyond this level strongly affects biodiversity and ecosystem structure and function^73^.

We did not conduct a full water stress assessment, for which all water demand stakeholders (such as agriculture^31^, municipal water use, mining and industrial water use) are required. Reason is that not for all of these stakeholders, spatially detailed data to the level of detail of our energy assessment, are available.

### Uncertainty analysis

To account for uncertainty for future power plants up to 2030 in INVENTORY, we account for delays in power plants becoming operational. We assume that for solar power plants and wind farms, with their relatively short planning and construction time^14^, a delay of one year occurs^74^. The ACP, e.g., reports solar and wind projects to be delayed by an average of 13 months^74^. As an African example, the 120 MW Kairouan Solar Photovoltaic Project in Tunisia was originally planned to become operational in 2024^75^, but this was delayed to 2025^76^. For all other power plants, which have on average longer planning and construction times^14^, we assume a delay of two years. Fossil-fuelled thermal plants show average construction times of almost 5 years^14^, so adding two years is an increase of 40%. An example is the Egbema gas-fired power station in Nigeria, which in INVENTORY is identified to become operational in 2024^77^, but was delayed to 2026^78^. In addition, we select planned nuclear power plants and large hydropower plants due to their long planning and construction times, and exclude them in case of a substantial possibility for a delay, based on recent information. As such, we quantify electricity production by 2030 for an INVENTORY analysis with substantial delays.

For our assessment on the water demand as a percentage of renewable water availability for major African basins for INVENTORY, we also account for uncertainty: we conducted the same analysis for an EF measure representative for minimum flow recommendations, i.e., the monthly *Q*_95_, the flow exceeded for 95% of each month^30, 60^. As such, we provide a range between a high (EFs by Richter et al. ^34^) and low (*Q*_95_) EF protection level. The results are shown in Supplementary Fig. 2.

In addition, we account for uncertainty in future electricity production forecasting up to 2030 based upon past time series data (from 1990 to 2015, as well as on the database of 2020 and its update for 2023), by using three different regression analyses as well as ARIMA.

### Power plants are becoming operational in 2024 and 2025

Our paper was first submitted in November 2024, while the final publication occurred in 2026. We therefore present our analysis up to the year 2023 as existing data, and all successive years (2024–2030) as forecasted data. At the beginning of 2026, we are, however, able to identify the additional power plants becoming operational in the years 2024 and 2025 based on real-world data. We compare the results in Supplementary Table 8.

## Supplementary information

Supplementary Information

Descriptions of Additional Supplementary Files

Supplementary Data 1-7

Transparent Peer Review file

## Source data

Source Data

## Data Availability

All data are provided open access as Supplementary Data files: Supplementary Data 1: INVENTORY, the inventory of power plants; Supplementary Data 2: Past, current and forecasted electricity generation by source (GWh) on the national level; Supplementary Data 3: REGR_MVAR national data; Supplementary Data 4: Annual and monthly evaporation amounts (in mm) per hydropower plant; Supplementary data 5: National values 1990–2030 Energy (GWh), water withdrawal (m^3^), water consumption (m^3^) and CO_2_ emissions (tCO_2_e); Supplementary data 6: Power plants for the NDC scenario, based on INVENTORY; Supplementary data 7: National amounts (per fuel type) for electricity production per installed capacity Source data are provided with this paper.

## Code availability

The Python code for the ARIMA analysis is available on the repository Zenodo 10.5281/zenodo.19602927^79^. Python is an open-source software.
