# Supplementary material for: Forecast on Africa’s power production up to 2030 with related water use and CO2 emissions
Source: Nat Commun. 2026 May 7;17:6154. doi: 10.1038/s41467-026-72692-2 (PMC13365816; doi:10.1038/s41467-026-72692-2)
Supplement: Supplementary file 1 — Supplementary Information [file 41467_2026_72692_MOESM1_ESM.pdf]

Supplementary Information for Forecast on Africa's power production up to 2030 with related water use and CO_2 emissions

S.D. Vaca-Jiménez[1,2], P.W. Gerbens-Leenes[2], Bunyod Holmatov[3], Raphael Vanham[4], Davy Vanham[3]

[1] Departamento de Ingeniería Mecánica, Escuela Politécnica Nacional, Ladrón de Guevara E11·253, 01-17-2759, Quito, Ecuador

[2] Integrated Research on Energy, Environment and Society (IREES), University of Groningen, Groningen, The Netherlands

[3] International Water Management Institute (IWMI) - Colombo, Sri Lanka

[4] Department of Mathematics, University of Innsbruck, Innsbruck, Austria

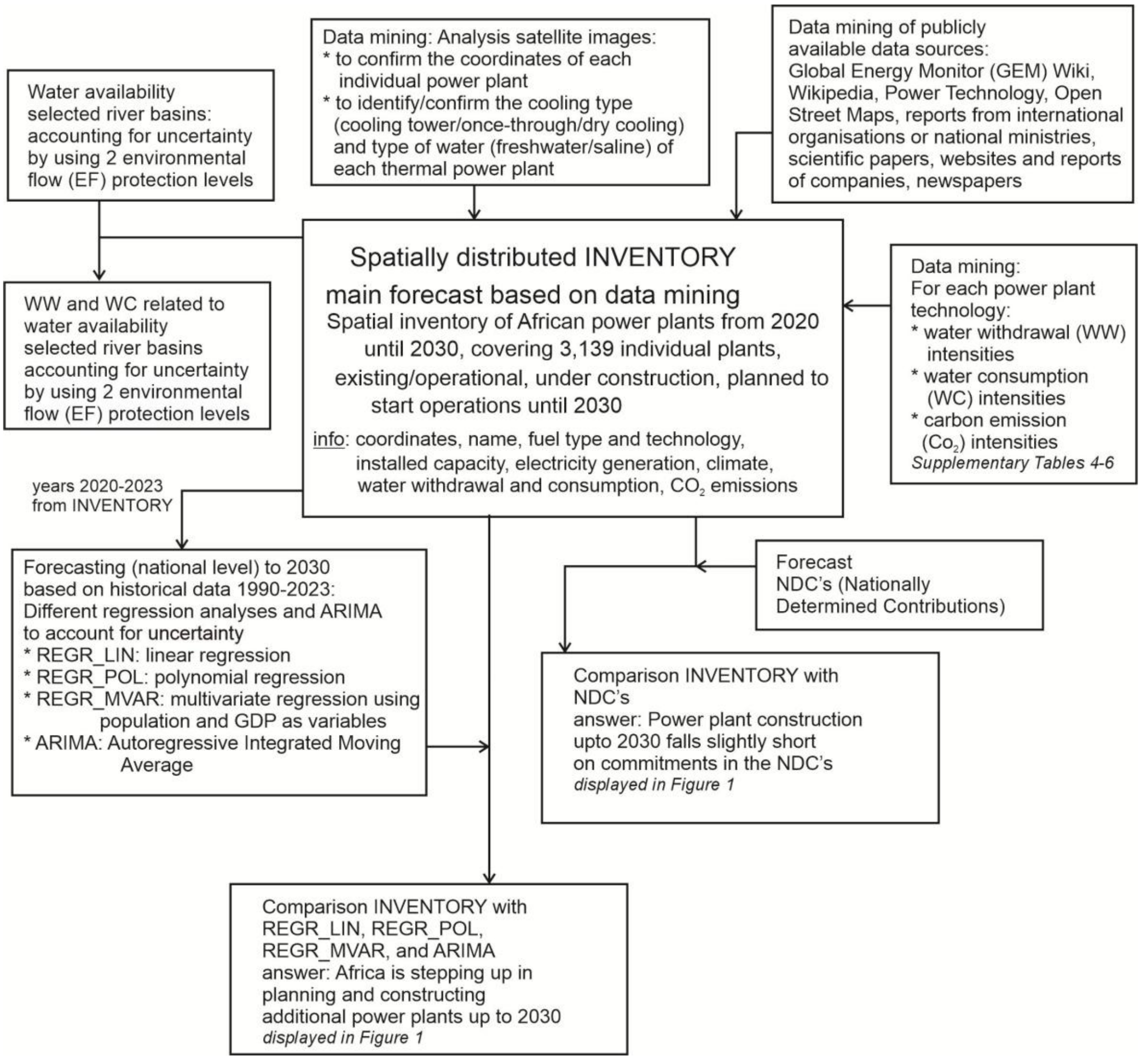

Supplementary Figure 1: Workflow methodology. This figure shows the main steps and components of the methodology used in our paper, to establish INVENTORY, the four forecasts based upon historical data as well as the forecast NDCs. As last step in the workflow, the results of INVENTORY are compared with the five other forecasts.

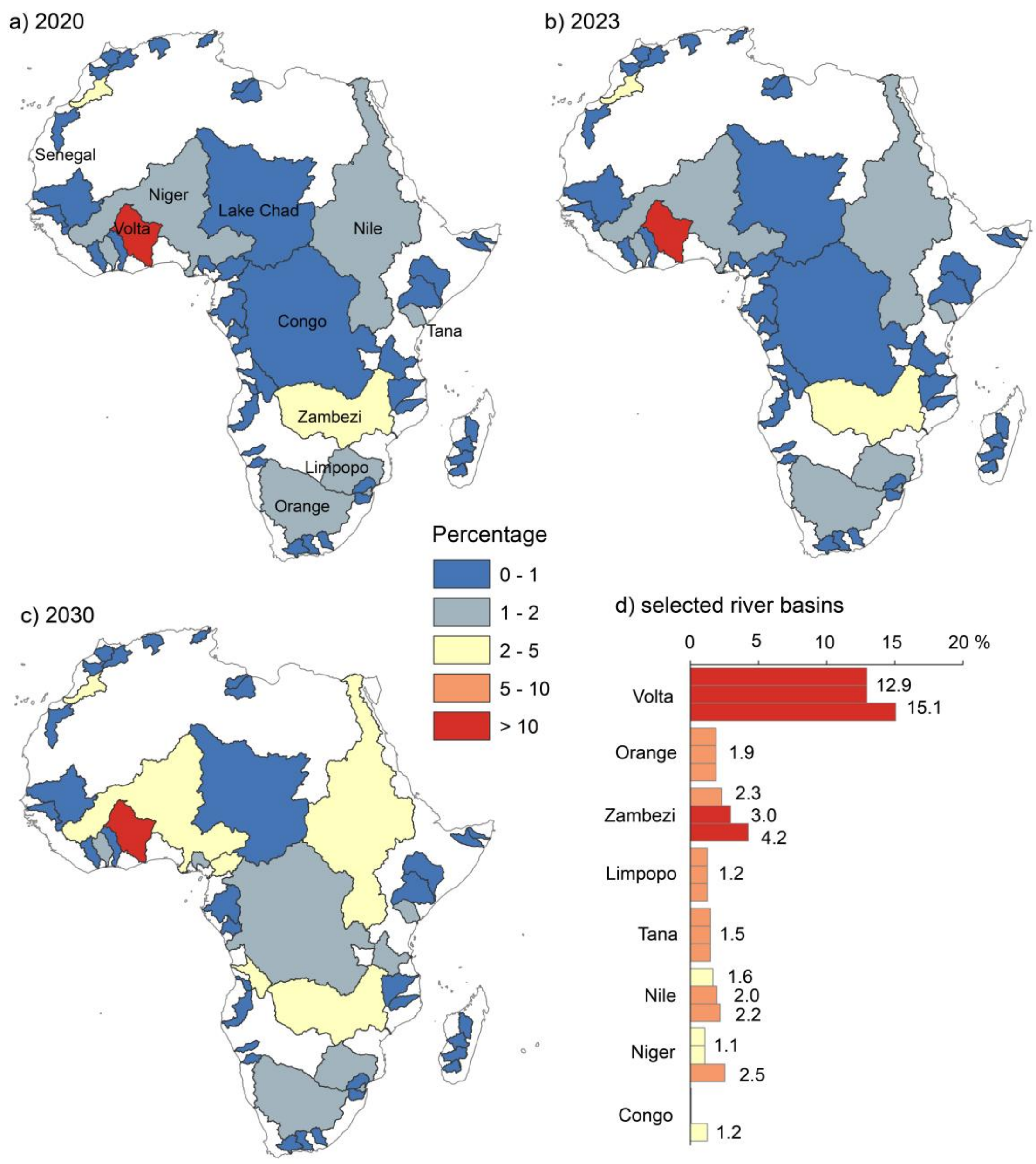

Supplementary Figure 2: Water withdrawal (WW) as percentage to renewable water availability (Environmental Flow EF=Q_{95}) in major African river basins, highlighting selected basins with the highest amounts, for INVENTORY for the years 2020 (a), 2023 (b) and 2030 (c). For 2030, only new power plants with known coordinates are included. d) shows amounts for selected river basins (upper bar 2020, middle bar 2023, lower bar 2030). Map in a, b and c created with ArcGis using continent borders from GADM (https://gadm.org/data.html)

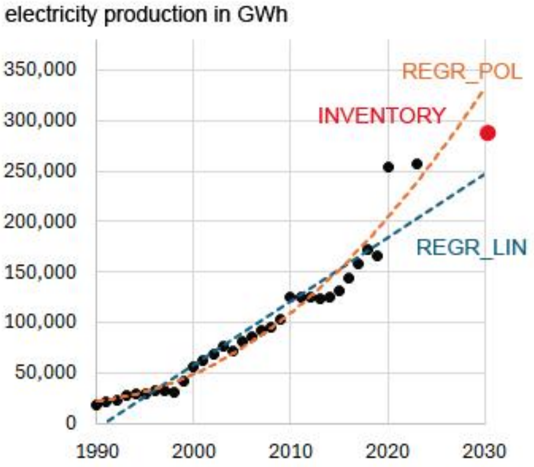

Year	natural gas GWh	Source
**1990**	18,931	IEA
**1995**	30,433	IEA
**2000**	56,743	IEA
**2005**	81,006	IEA
**2010**	125,573	IEA
**2015**	132,126	IEA
**2020**	253,400	INVENTORY
**2023**	256,742	INVENTORY
**2030**	290,810	INVENTORY
**2030**	247,599	REGR_LIN
**2030**	248,648	REGR_MVAR
**2030**	304,279	ARIMA
**2030**	332,388	REGR_POL

REGR_LIN (Eq 3): $EL_{2030,Egypt,natural\ gas} = 6{,}298.5039854 * 2030 - 12{,}538{,}364 = 247{,}599$ with $R^2 = 0.90$

REGR_POL (Eq 4): $EP_{2030,Egypt,natural\ gas} = 169.48971 * 2030^2 - 673{,}613.33021 * 2030 + 669{,}317{,}302 = 289{,}371$ with $R^2 = 0.95$

REGR_MVAR (Eq 5): $EMV_{2030,Egypt,natural\ gas} = r * GDP + s * Population + t$

$= 0.000000093671 * 443{,}017{,}890{,}874 + 0.003102789 * 124{,}740{,}510 - -179{,}893$ with $R^2 = 0.96$

Supplementary Figure 3: INVENTORY value for 2030 (red dot in graph) compared to regression analysis forecasts (REGR_LIN, REGR_POL and REGR_MVAR) and the ARIMA forecast for 2030 for natural gas fired electricity production in Egypt. The national Gross Domestic Product (GDP) and population are projected to 443 billion USD and 125 million people, respectively.

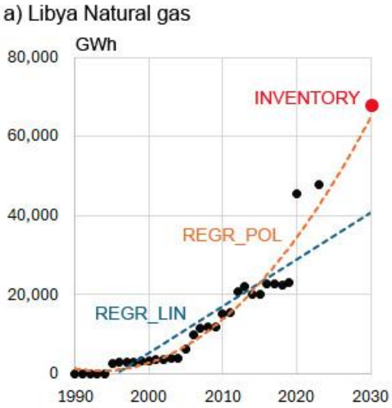

Year	GWh	Source
1990	0	IEA
1995	2,600	IEA
2000	3,400	IEA
2005	6,336	IEA
2010	15,344	IEA
2015	20,262	IEA
2020	45,461	INVENTORY
2023	47,907	INVENTORY
2030	67,591	INVENTORY
2030	39,965	REGR_MVAR
2030	40,796	REGR_LIN
2030	47,820	ARIMA
2030	64,856	REGR_POL

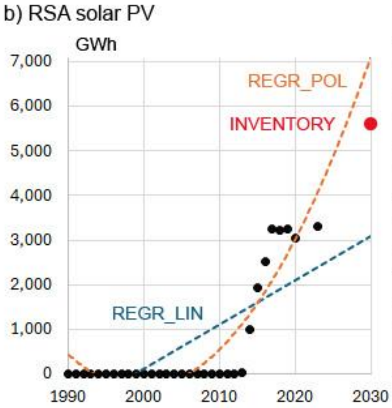

Year	GWh	Source
1990	0	IEA
1995	0	IEA
2000	0	IEA
2005	0	IEA
2010	0	IEA
2015	1,946	IEA
2020	3,062	INVENTORY
2023	3,318	INVENTORY
2030	5,673	INVENTORY
2030	3,165	REGR_MVAR
2030	3,089	REGR_LIN
2030	3,554	ARIMA
2030	7,107	REGR_POL

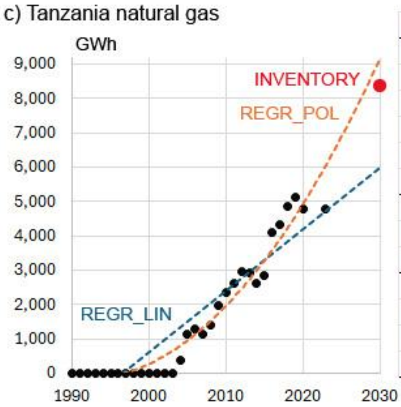

Year	GWh	Source
1990	0	IEA
1995	0	IEA
2000	0	IEA
2005	1,149	IEA
2010	2,351	IEA
2015	2,863	IEA
2020	4,802	INVENTORY
2023	4,802	INVENTORY
2030	8,346	INVENTORY
2030	6,010	REGR_MVAR
2030	5,992	REGR_LIN
2030	4,814	ARIMA
2030	9,155	REGR_POL

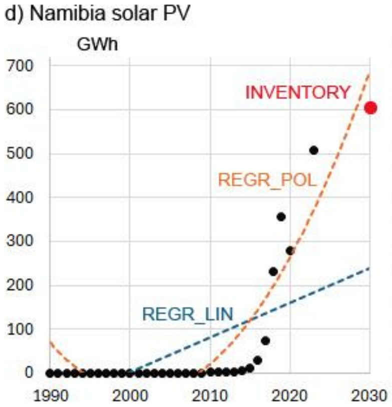

Year	GWh	Source
1990	0	IEA
1995	0	IEA
2000	1	IEA
2005	3	IEA
2010	7	IEA
2015	39	IEA
2020	280	INVENTORY
2023	508	INVENTORY
2030	608	INVENTORY
2030	264	REGR_MVAR
2030	248	REGR_LIN
2030	570	ARIMA
2030	711	REGR_POL

Supplementary Figure 4: INVENTORY value for 2030 (red dot in graph) compared to regression analysis forecasts (REGR_LIN, REGR_POL and REGR_MVAR) and ARIMA for 2030 for electricity production of selected fuel types in selected countries. For all 4 panels a to d, REGR_POL provides the forecast closest to the INVENTORY amount. a) natural gas in Libya; b) solar PV in the RSA; c) natural gas in Tanzania and d) solar PV in Namibia

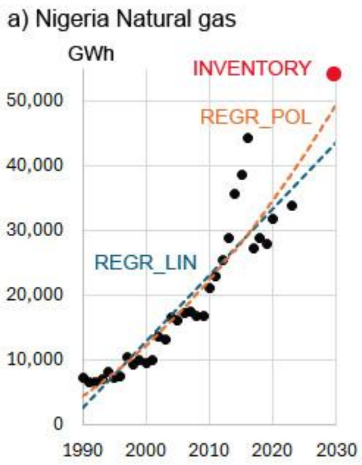

Year	GWh	Source
**1990**	7,223	IEA
**1995**	7,331	IEA
**2000**	9,467	IEA
**2005**	16,169	IEA
**2010**	21,221	IEA
**2015**	38,578	IEA
**2020**	31,759	INVENTORY
**2023**	33,772	INVENTORY
**2030**	53,618	INVENTORY
**2030**	43,890	REGR_MVAR
**2030**	43,729	REGR_LIN
**2030**	35,196	ARIMA
**2030**	49,610	REGR_POL

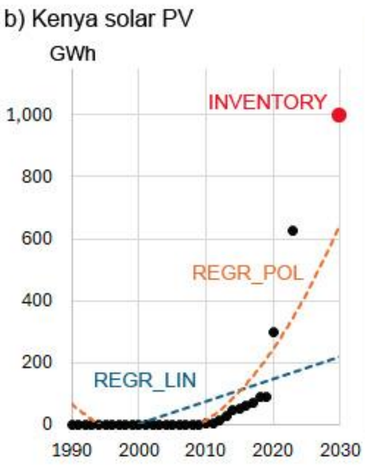

Year	GWh	Source
**1990**	0	IEA
**1995**	0	IEA
**2000**	0	IEA
**2005**	0	IEA
**2010**	3	IEA
**2015**	50	IEA
**2020**	299	INVENTORY
**2023**	627	INVENTORY
**2030**	994	INVENTORY
**2030**	219	REGR_MVAR
**2030**	221	REGR_LIN
**2030**	1,138	ARIMA
**2030**	651	REGR_POL

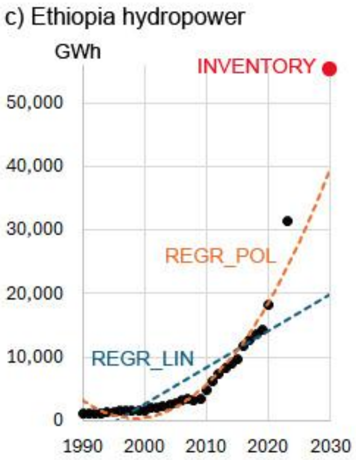

Year	GWh	Source
**1990**	1,062	IEA
**1995**	1,428	IEA
**2000**	1,646	IEA
**2005**	2,833	IEA
**2010**	4,931	IEA
**2015**	9,674	IEA
**2020**	18,277	INVENTORY
**2023**	31,318	INVENTORY
**2030**	55,759	INVENTORY
**2030**	20,020	REGR_MVAR
**2030**	20,037	REGR_LIN
**2030**	63,070	ARIMA
**2030**	39,936	REGR_POL

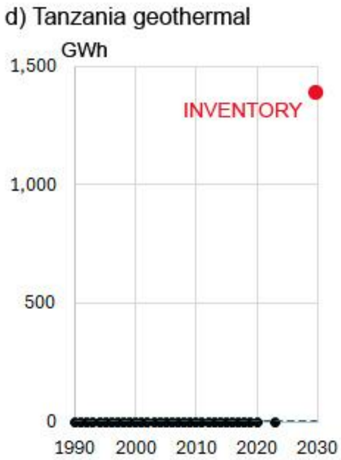

Year	GWh	Source
**1990**	0	IEA
**1995**	0	IEA
**2000**	0	IEA
**2005**	0	IEA
**2010**	0	IEA
**2015**	0	IEA
**2020**	0	INVENTORY
**2023**	0	INVENTORY
**2030**	1,349	INVENTORY
**2030**	0	REGR_MVAR
**2030**	0	REGR_LIN
**2030**	0	ARIMA
**2030**	0	REGR_POL

Supplementary Figure 5: INVENTORY value for 2030 (red dot in graph) compared to regression analysis forecasts (REGR_LIN, REGR_POL and REGR_MVAR) and ARIMA for 2030 for electricity production of selected fuel types in selected countries. a-d present situations where all regressions underestimate the INVENTORY amount. a) Natural gas in Nigeria; b) solar PV in Kenya; c) hydropower in Ethiopia and d) geothermal in Tanzania

Supplementary Table 1: Comparison of data entries (number of power plants and capacity in MW) between our database, WRI's Global Power Plant Database[1] and the Renewable Power Plant database for Africa (RePP Africa)[2]

	This study				WRI's Global Power Plant Database[1]		Renewable Power Plant database for Africa (RePP Africa)[2], study published in 2023			
	year 2022		year 2020		Year 2020 (most recent year)		year 2022 (most recent year)		Year 2020	
	Number	Capacity (MW)	Number	Capacity (MW)	Number	Capacity (MW)	Number	Capacity (MW)	Number	Capacity (MW)
Oil	1,061	23,272	1,054	22,265	102	8,425				
Coal	51	52,202	49	49,807	31	45,097				
Natural gas	358	125,512	343	119,263	134	64,293				
Hydropower (reservoir+ROR)	581 (195 reservoir +386 ROR)	41,864	561 (183 reservoir +378 ROR)	36,892	163	30,399	336 (178 reservoir+121 ROR+37 undefined)	37,849	331 (178 reservoir+118 ROR+35 undefined)	37,070
Wind	100	7,859	83	5,646	42	4,499	117	9,024	102	7,631
Sun	398	7,072	299	6,150	129	4,893	348	8,313	282	7,163
Biomass	127	2,066	121	1,958	15	320				
Geo	9	896	8	813	7	761				
Nuclear	1	1,860	1	1,860	1	1,800				
Waste heat	15	951	15	951	7	46				
total	2,701	263,554	2,534	245,604	631	160,533				

Supplementary Table 2: Data sources used for electricity production per technology, for the different years in our assessment

Country	Electricity production per technology	
	Period	Source
Algeria	1990 - 2019	[3]
	2020	[4]
	2030 - NDC	[5,6]
Angola	1990 - 2019	[3]
	2020	[4]
	2030 - NDC	[7]
Benin	1990 - 2019	[3]
	2020	[4]
	2030 - NDC	[8]
Botswana	1990 - 2019	[3]
	2020	[4]
	2030 - NDC	[9,10]
Burkina Faso	1990 - 2019	[3]
	2020	[4]
	2030 - NDC	[11,12]
Burundi	1995 - 2019	[13]
	2020	[4]
	2030 - NDC	[14]
Cameroon	1990 - 2019	[3]
	2020	[4]
	2030 - NDC	[15]
Cape Verde	2009 - 2016	[16]
	2020	[4]
	2030 - NDC	[17]
Central African Republic	2000 - 2019	[18]
	2020	[4]
	2030 - NDC	[19]
Chad	1990 - 2019	[3]
	2020	[4]
	2030 - NDC	[20]
Republic of Congo	1990 - 2019	[3]
	2020	[4]
	2030 - NDC	[21]
Democratic Republic of Congo	1990 - 2019	[3]
	2020	[4]
	2030 - NDC	[22]
Cote d’Ivoire	1990 - 2019	[3]
	2020	[4]
	2030 – NDC	[23]
Djibouti	2000 - 2019	[18]
	2020	[4]
	2030 – NDC	[24]
Egypt	1990 - 2019	[3]
	2020	[4]
	2030 – NDC	[25,26]
Equatorial Guinea	1990 - 2019	[3]
	2020	[4]
	2030 – NDC	[27]
Eritrea	1992 - 2019	[3]
	2020	[4]
	2030 – NDC	[28]
Ethiopia	1990 - 2019	[3]
	2020	[4]
	2030 – NDC	[29]
Gabon	1990 - 2019	[3]
	2020	[4]
	2030 – NDC	[30]
Gambia	2000 - 2019	[18]
	2020	[4]

Country	Electricity production per technology	
	Period	Source
	2030 – NDC	[31]
Ghana	1990 - 2019	[3]
	2020	[4]
	2030 – NDC	[32]
Guinea	2000 - 2019	[18]
	2020	[4]
	2030 – NDC	[33]
Guinea Bissau	2000 - 2019	[18]
	2020	[4]
	2030 – NDC	[34]
Kenya	1990 - 2019	[3]
	2020	[4]
	2030 – NDC	[35,36]
Lesotho	2000 - 2019	[18]
	2020	[4]
	2030 – NDC	[37]
Liberia	2000 - 2019	[18]
	2020	[4]
	2030 – NDC	[38]
Libya	1990 - 2019	[3]
	2020	[4]
	2030 – NDC	[39]
Madagascar	1990 - 2019	[3]
	2020	[4]
	2030 – NDC	[40]
Malawi	2000 - 2019	[18]
	2020	[4]
	2030 – NDC	[41]
Mali	2010, 2015	[42]
	2020	[4]
	2030 – NDC	[43]
Mauritania	2000 - 2019	[18]
	2020	[4]
	2030 – NDC	[19]
Mauritius	2000 - 2019	[18]
	2020	[4]
	2030 – NDC	[44,45]
Morocco	1990 - 2019	[3]
	2020	[4]
	2030 – NDC	[46]
Mozambique	1990 - 2019	[3]
	2020	[4]
	2030 – NDC	[47]
Namibia	1991 - 2019	[3]
	2020	[4]
	2030 – NDC	[48]
Niger	1990 - 2019	[3]
	2020	[4]
	2030 – NDC	[49]
Nigeria	1990 - 2019	[3]
	2020	[4]
	2030 – NDC	[50]
Rwanda	1990 - 2019	[3]
	2020	[4]
	2030 – NDC	[51]
Sao Tome & Principe	2000 - 2019	[18]
	2020	[4]
	2030 – NDC	[52]
Senegal	1990 - 2019	[3]
	2020	[4]
	2030 – NDC	[53]
Seychelles	2000 - 2019	[18]

Country	Electricity production per technology	
	Period	Source
	2020	4
	2030 – NDC	54
Sierra Leone	2000 - 2019	18
	2020	4
	2030 – NDC	55
Somalia	2000 - 2019	18
	2020	4
	2030 – NDC	56
South Africa	1990 - 2019	3
	2020	4
	2030 – NDC	57,58
South Sudan	2012 - 2019	3
	2020	4
	2030 – NDC	59
Sudan	1990 - 2019	3
	2020	4
	2030 – NDC	60
Tanzania	1990 - 2019	3
	2020	4
	2030 – NDC	61
Togo	1990 - 2019	3
	2020	4
	2030 – NDC	62
Tunisia	1990 - 2019	3
	2020	4
	2030 – NDC	63
Uganda	1990 - 2019	3
	2020	4
	2030 – NDC	64
Zambia	1990 - 2019	3
	2020	4
	2030 – NDC	65
Zimbabwe	1990 - 2019	3
	2020	4
	2030 – NDC	66

Supplementary Table 3: List of data sources for including the power plants that started operations between 2021 and 2023, per country.

Country	Name	Source
Algeria	El Menea (Menia)	[67]
	Ghardaïa	[68]
	Oumache II	[68]
	Naama	[68]
Angola	Rio Luachimo I	[69]
	Lubango Subestacao	[70]
Benin	Maria-Gleta Genesis	[69]
Burkina Faso	Nagreongo Solar Power Station	[69]
	Kodeni Solar Power Station	[69]
	Burkina Prefectural Project	[71]
Burundi	Mubuga Solar Power Station	[13]
Cameroon	Mekin	[69]
Cape Verde	Sal	[72]
Central African Republic	Bangui Enerca expansion	[73]
	Boali-III expansion	[74]
	Danzi Solar Power Station	[69]
	Sakaï Solar Power Station	[69]
Chad	Sarh	[75]
	N'djamena	[76]
	Starsol (in N'djamena)	[67]
Democratic Republic of Congo	Kiliba Mill	[69]
	Busanga	[77]
Cote d'Ivoire	Azito	[67]
Egypt	Cairo West Extension	[67]
	Walidia	[67]
	West Bakr Wind Farm	[67]
	Kima Plant	[78]
	Borg el Arab Plant	[67]
Ethiopia	Gigel Gibe-IV (Koysha)	[79]
	Aysha	[68]
	Grand Renaissance First Stage	[69]
Ghana	Bui Dam Hybrid Solar	[80]
	Komenda Sugar	[81]
	Kaleo Solar Farm	[82]
Guinea	Souapiti	[69]
	Lefa Corrido	[83]
	Gardete Solar Farm	[67]
Kenya	Olkaria-I	[69]
	Radiant Solar	[67]
	Kilifi County	[67]
	Enkasiti Flower Farm	[84]
	Brava Foods Beverages	[84]
	Ken Salt Factory	[84]
	Kikwetu Flowers	[84]
	Sotik Arroket East Tea	[84]
	Rupa Mall	[84]
	Sotik Arroket West Tea	[84]
	Sotik Mettarora Tea	[84]
	Shade Net Plastics	[84]
	Ken Knit	[84]
	Enkasiti Flower Farm	[84]
	Sirikwa Quarry Construction	[84]
	Evergreen Athi River Agriculture	[84]
	Panocal International	[84]
	Spinners & Spinners	[84]
	Maisha Beverages	[84]
	Nyati Feeds	[84]
	Ken Salt Factory	[84]
	Bufalo Millers	[84]
	Tai Enterprises Construction	[84]

Country	Name	Source
	Lakhir Plastics	84
	Heritage Horticulture	84
	Utee flower Farm (expansion)	84
	Sagana Tanneries	84
	Malindi Solar	84
	Milly Glass Factory	84
	Ken Salt Malindi	84
	Doshi Water	84
	Ndarawetta Tea	84
	Greenfields Tea (expansion)	84
	Vipingo Ridge Estate	84
	Mau Tea	84
	Kabianga tea, tea estate	84
	Lemottit flower farm	84
	Interconsumers product factory	84
	Fortuna industries	84
	Khetias new kabernet factory	84
	Eslon plastics 1	84
	Maisha millers	84
	Elgon tea	84
	Khetias hq kitale	84
	Shalem Factory	84
	Eslon Plastics 2	84
	Sintel Factory	84
	Advanced Plastics	84
	Kapi Factory	84
	Ecoline Factory	84
	Solai Nakuru Flower Farm	84
	Metro Poli Plastics	84
	Halar Industries	84
	Addison Industries	84
	Kethias Giga	84
	Magadi Works	85
	Kopere Solar	68
	Rupiongazi KTDA	86
	Mpeketoni Wind	68
	Ol Ndanyat	67
	Akiira	69
	Meru County	69
	Alten Solar	69
Liberia	Congo Town Expansion	87
Libya	Semno	88
	Tripoli West	88
Madagascar	Tanabo	89
	Ambatolampy	69
	Centrale solaire d'andranotakatra	90
	Antalaha	91
	Kimoni	69
	Nosy be	92
	Sambava solar power station	93
	Centrale solaire d'ambavahadinilakaka	94
	Farahantsana	95
	Qit ilmenite	96
	Sava hybride power station	97
	Betsinjaka Toliara	98
	Ambohimanambola hydelec	99
Malawi	Likoma island	100
	Chizumulu Island	100
	Songwe Power Station	69
	Salima Solar	101
	Golomoti	101
	Bwengu Solar Power Station	68
	Zomba	102

Country	Name	Source
Mali	Gouina	67
	Niono (Nampala Mine)	103
	Syama Gold Project	69
Mauritania	Boulenouar Wind	69
Mauritius	CEB PV Farm (Henrietta)	104
	SSDG	104
	MSDG	104
	Sarako PV farm	68
	Solar field ltd	105
	Synnove energy Esperance	105
	Voltas green ltd PV farm	105
	Voltas yellow PV farm	105
	Helios beau champ ltd pv farm	105
	Akuo pv farm	105
	Synnove petite retraite	105
	SPV petite reviere	105
	Eole wind farm	105
	Ceb hydro generation	68
Morocco	Tanger Khalladi	67
	Taza	68
	Midelt	68
	Jbel Lahdidi	67
	Al Koudia	67
	Akhfennir	67
Mozambique	Temane	69
	Rvuma Gas	67
Namibia	Terasun	69
	Diaz (Luderitz)	69
	Anixas II	67
Niger	Gourou Banda’s Solar Project	69
Nigeria	Afam (FIPL)	67
	Northrich Cement Plant	106
	Gurara Dam	69
	Katsina Wind	107
	Aba Power	108
	Oando Project	67
	Okpella Cement Plant	109
	Zungeru	69
	Ikeja Bottling Cogen	110
	Alausa	111
	Tiga Dam	112
Rwanda	Rubavy Biogas	113
	Symbion Kibuye	69
	Rukarara V	114
	Rusumu Falls	69
Sao Tome & Principe	Santo Amaro	115
Senegal	Sococim Cement Plant	116
	Taiba Ndiaye	69
	Diass Power Station	69
	Cap-des-Biches	67
	Sabodala Gold Mine	117
Seychelles	Romainville Solar Park	118
Sierra Leone	Bo-Kenema Solar	119
Somalia	Bosaso	120
South Africa	Medupi	68
	Greefspan	68
	Kruisvallei	121
	Golden Valley Wind	69
	Kangnas	69
	Perdekraal East	69
	Roggeveld	69
	Ngodwana Mill	122

Country	Name	Source
	Felixton Mill	[123]
	Kusile	[67]
	Bellville Health Park	[124]
	Orange Loeriesfontein	[67]
	Garob	[69]
South Sudan	Juba	[67]
Sudan	Dangola Wind Farm	[68]
	Atbara Cement Plant	[125]
Tanzania	Mafia Biogas + expansion	[126]
Togo	Kékéli	[69]
	Sheikh Mohammed Bin Zayed	[127]
Tunisia	Rades C	[67]
	Tozeur Solar (extension)	[67]
Uganda	AfroPlast Factory	[84]
	Kiko Tea Factory	[84]
	Karuma Falls	[69]
	Achwa 1	[69]
	Nyamwamba II	[69]
	Kikagati – Murongo	[69]
	Muyembe	[128]
Zambia	Lusaka South MFEZ	[129]
	Kafue Gorge Lower	[69]
Zimbabwe	Venice Mines	[84]
	Turk Mine	[84]
	Golden Quarry	[84]
	Hwange III	[67]

Supplementary Table 4: List of data sources to identify the power plants for the period 2024 until 2030, per country

Country	Name	Source
Algeria	Oumache III	[68]
	Djelfa	[67]
	Illizi	[68]
	Mostaganem	[67]
	Bechar - Kenadsa	[68]
	Kais	[67]
Angola	Caculo-cabaca	[68]
	Malongo terminal	[67]
	Chicapa-II	[130]
	Cavango	[131]
	Mabudas dam	[132]
	Chiumbe-dala	[133]
	Soyo LNG plant	[134]
	Jamba ia mina	[68]
	Luapasso	[131]
	Malanje biocom	[68]
	Rio cutato	[135]
	Soyo CC	[134]
	Calengue	[68]
	Baynes	[67]
Benin	Maria-Gleta terminal (ii)	[67]
Benin	Port de Cotonou, FPV	[67]
	Yeripao	[136]
	Glo-djigbé thermal	[137]
	Bohicon	[138]
	Djougou innovent	[138]
	Djougou Benin II	[138]
	Kandi ipp greenheart	[138]
	Natitingou	[138]
	Onigbolo	[138]
	Parakou	[138]
	Regions agricole du Nord	[138]
	Dogo (Ketou-Dogo BIS)	[67]
	Vossa	[68]
	Bétérou	[139]
Botswana	Sese block	[68]
	Letlhakane CSP station	[69]
	Maun CSP station	[69]
	Jwaneng solar	[67]
	Gaborone	[140]
	Shumba solar	[68]
Burkina Faso	Noumbiel	[141]
	Bougouriba	[136]
	Zina solar	[69]
Burundi	Jiji River	[141]
	Mulembwe	[69]
	Kabu-16	[67]
	Kagu 006	[67]
	Mpanda Burundi	[67]
Cameroon	Nachtigal	[142]
	Centrale Limbe	[143]
	Menchum KPEP	[68]
	Bini a Warak	[67]
	Songmbengue	[68]
	Colomines (Kadei)	[144]
Cape Verde	Boa Vista island	[72]
Central African Republic	Lobaye	[19]
	Microcentrales	[19]
Chad	Amea Solar Station	[69]
Republic of Congo	Cote Mateve	[67]

Country	Name	Source
	Sounda Gorge	69
Democratic Republic of Congo	Katende	69
	Ruzizi III	68
	Tshopo	145
	Sombwe	68
	Tshibashi	146
	Kinkasi	147
	Grand Inga Power Station	69
	Inga III Power Station	69
	Ruzizi IV	68
	Muanda	67
	Piana Mwanga	69
	Tshibwe	69
Cote d'Ivoire	Karpowership	148
	Songon	67
	Singrobo	149
Djibouti	Lake Assal	150
	Arta Djibouti	151
Egypt	Sinai	68
	Dairut	68
	Al-Arish	68
	Naga Hammadi Smelter	68
	Kom Ombo Capital	68
	Zafarana Scatec	152
	Hurghada Wind II	153
	Gulf of Suez	68
	Dabaa	68
	Borg el Arab	154
	Hurghada North	67
	Hurghada South	67
	Abu Rudeis	67
	El Aft, Kafr el Sheikh	67
	Damanhour New	68
Equatorial Guinea	Sendje	68
Ethiopia	Tendaho	68
	Geba-2	68
	Genale-Dawa-VI	67
	Awash	68
	Corbetti	68
	Aluto Langano	67
	Geba-1	68
	Grand Renaissance	69
	Aysha (2nd phase)	155
Gabon	Owendo	156
	Oyem	69
	Fe-2 (Kinguele Aval)	69
	Ngoulmendjim	69
	Imperatrice (Mouila)	67
Gambia	Soma Solar Station	67
	Tropic Center Retail	84
	Brikama	157
	Farafenni	138
	Basse Region	69
Ghana	Nante Solar Park	68
	Numasi Project	68
	Hemang	68
	Awisam	68
	Juale	68
	Tanoso	68
	Chirano Mine IGCC	158
	Tarkwa Gold Mine	158
	MIM Sawmill	159
	Bridge Power	68

Country	Name	Source
	Esiama	160
	Inchaban	161
	Wassa Mine	162
	Bole Solar PV Park	68
	Ada Foah Tidal	69
	Ankobra River	163
	Kulpawn	164
	Pwalugu	164
	Daboya	164
	Ayitepa	165
	Sege Wind (Koluedor)	67
Guinea	Kiniero Mine	166
	Amaria Hydropower	68
	Fomi (Niandan River)	69
	Conakry Wave	167
	Tipo (Kogon River)	67
	Khoumagueli Solar	69
Kenya	Pokot cement	67
	Limuru wind	67
	Karura	69
	Menengai Geothermal	68
	Munyu	68
	Tarda kiambere	168
	Masinga	168
	Tindinyo	169
	Kajiado cement plant	170
	Cedata	67
	Nithi power	171
	Mt Elgon	172
	Nandi	69
	Embu	172
	Murang'a	172
	Kipeto wind	69
	Chemosit river	173
	Ewaso ngiro	174
	Kapkoros kipsonoi	175
	Kenya wave	176
	Kipsonoi-I	177
Liberia	Kaiha River	178
	Monrovia Solar	179
	Hummingbird Dugbe	180
	Lower Falls (Gbedin)	181
Libya	Al khums	67
	Amal field	67
	Zliten	67
	Darnah	67
	Semno	67
	Kufra	182
	Bani walid	67
	Traghen Solar Park	183
	Sebha South	67
Madagascar	Sahofica	69
	Access solar power	69
	Volobe Hydelec	69
	Volobe	69
	Ambodiroka	68
	Antetezambato	184
	Amboasary Hydelec	184
Malawi	Kammwamba	67
	Mpatamanga Gorge	185
	Kholombidzo	69
	Karonga Eland	186
Mali	Sikasso	69

Country	Name	Source
	Segou Solar	69
	Fana Solar	69
	Gourbassi	187
	Kenie	69
	Taoussa	188
	Sotuba	43
	Boueina/Kayes	43
Mauritania	Thermal Power plants	189
Mauritius	Mauritius Solar Farm	67
Morocco	Dakhla	68
	Tanger melloussa	67
	Zagora solar	67
	Abdelmoumen	190
	Tarfaya sale	191
	El Menzel	68
	Al Koudia	67
	Dakhla Desalination	192
Mozambique	Chemba One	67
	Lichinga	69
	Beluluane Gas	68
	Niassa Solar	68
	Chemba Two	67
	Mphanda Nkuwa	193
	Rio Lurio	67
	Extension Tsate	67
	Moamba Major	194
Namibia	Baynes	69
	Sperrgebiet	195
	Oranjemund	67
	Omburu	69
	Kudu	69
	Langer Heinrich Mine	196
	Kahn Solar	69
	Otjikoto	67
Niger	High Efficiency TPP	49
	Niamey Solar	68
	Kandadji Dam	69
	Malbaza	197
Nigeria	Warri Refinery	198
	Egbema	67
	Alscon Smelter	67
	Geregu NPP	69
	Gurara Falls	69
	Lokoja Solar	67
	Agura	67
	Kano	68
	Ekiti	138
	Alaoji	67
	Agura	67
	Onitsha	68
	Ikom	68
	Itobe Project	68
	Bayelsa Benco	199
	Southern Swamp Aggp	67
	Sparkle Energy	200
	Abiba Solar	68
	Nova Scotia Power	201
	Oriental Jigawa	67
	Panyam Solar	67
	Shiroro Solar	202
	Yobe Solar	68
	Itu NPP	69
	Agura	67

Country	Name	Source
	Farin Ruwa	[203]
	Mambilla	[69]
	Okpai	[67]
Rwanda	Akanyaru	[69]
	Nyabarongo-II	[69]
	Ruhengeri-1	[204]
	Gisagara	[69]
	Kibuye Orascom	[205]
	Kayonza	[206]
	Rukarara VI	[207]
	Gakenke Base I and II	[208]
	Ngororero	[209]
	Rwondo	[209]
	Muhembe	[209]
	Nyirahindwe I and II	[209]
Sao Tome & Principe	Neves	[69]
	Rio Contador	[210]
Senegal	Sambangalou	[69]
	Niakhar	[68]
	Sendou	[67]
Seychelles	Flotaing Solar Park	[211]
Sierra Leone	Freetown Solar Park	[68]
	Marampa Mine New	[212]
	Moyamba Singimi	[213]
	Bumbuna II Yiben	[214]
South Africa	Cape Town Solar Plant	[215]
	Coega	[68]
	Rooikat and Meerkat	[216]
	Eastern Cape (Coega)	[68]
	Riemvasmaak	[217]
	York Timbers	[218]
	Red Stone	[69]
	Saldanha	[68]
	George Biomass	[219]
	Mkuze Biomass Power Plant	[68]
	Rustenburg Mine PV Park	[68]
South Sudan	Rumbek	[220]
	Bor	[221]
	Tharjath	[222]
	Polouge	[221]
	Fula Nimule	[69]
	Bedden	[223]
	Fula Rapids	[224]
	Off grid solar projects	[221]
Sudan	Port Sudan	[67]
	Al-Fula	[67]
	El Gaili	[67]
	Khartoum Refinery	[225]
	El Fasher (Darfur)	[226]
	Nyala Wind Farm	[67]
	Al-Fula Combined	[68]
	Kajbar	[69]
Tanzania	TGDC Projects	[227]
	Nachu Graphite	[228]
	Rumakali	[69]
	Mchuchuma	[67]
	Symbion Mtwara	[68]
	Mbeya (Kiwira)	[67]
	Ruhudji	[69]
	Kakono	[69]
	Mufindi Paper Mill	[67]
	Bagamoyo Sugar	[229]
	Kilwa Masoko	[69]

Country	Name	Source
	Rusomo Falls	[69]
	Igamba Malagarasi	[69]
	Malagarasi River	[69]
	Makambako	[68]
Tunisia	Kairouan Solar Park	[68]
	SFAX STEG	[67]
	Amea Power Solar Park	[230]
Uganda	Atiak Mill	[69]
	Katwe Geothermal	[67]
	Pabbo Solar	[68]
	Achwa 3,4,5	[69]
	Nyamagasani 1	[69]
	Ayago North	[69]
	Ayago South	[69]
	Rukungiri	[231]
	Cresta Buhindagi	[232]
	Elgon Sisi	[233]
	Muzizi	[69]
	Nengo Bridge	[69]
	Nsongezi	[69]
	Karamoja Wind Farm	[67]
	Rupa Windpower	[64]
	Xsabo Nkoge Solar	[64]
	Scoul Bagasse Plant	[64]
Zambia	Kabwelume	[68]
	Lunsemfwa Lower	[69]
	Kapisya	[234]
	Kundabwika	[68]
	Luapula River	[235]
Zimbabwe	Great Zimbabwe	[236]
	Bulayao Solar	[68]
	Gwanda CSP	[68]
	Gwanda Solar I and II	[68]
	Harava Solar Station	[69]
	Chisumbanje	[68]
	Bulawayo	[67]
	Gokwe North	[67]
	Lusulu	[67]
	Blanket Gold Mine	[68]
	De Green	[68]
	Munyati Solar	[67]
	Gairezi	[237]
	Honde Valley	[238]
	Osborne Dam (Ngonyezi)	[69]

Supplementary Table 5: Water Withdrawal intensities for each power plant technology included in our assessment. *The water intensities are defined based on climate zones. The climate zones were obtained from Kottek et al.* [239]*, and describe the following: Af - Tropical Rainforest, Am - Tropical Monsoon, Aw - Tropical Savannah, BSh - Arid Steppe – Hot, BWh - Desert Hot, Cfa -Humid subtropical climate, Cfb - Temperate Without dry Season, Csa - Temperate Dry Summer, Csb - Temperate Dry Summer, Cwa - Temperate Dry Winter, and Cwb - Temperate Dry Winter*

Technology	1st Category	2nd Category	3rd Category	Fuel	Climate	Source	Value [M^3/MWh]	Assumption
Biomass	Rankine	Steam Turbine	No cooling	Various crops	NA	Estimation based on the consumption.	0.162	As there are no estimates directly for this technology, it is considered similar than coal. Considering that there is no freshwater consumption for the cooling system, assumed similar ratio than cooling towers 70% in Dziegielewski & Bik [240].
			Once through (Fresh)	Various crops	Aw<br>BWh	[241]	189.271	There are no specific data points about this type. Used the maximum reported value for coal-fired power plants with both consumption and withdrawal data, considering that these climates require more water. In reality, the real withdrawal could be higher because this value comes from the USA, which averages with more temperate climates
			Wet Tower	Various energy crops	Af<br>Am<br>Aw	[242]	4.542	There are no specific data points about this power plant. Used the estimations of the coal-fired power plants. Used the maximum reported value considering that these climates require more water. In reality, the real withdrawal could be higher because this value comes from EIA Form 767 report from the USA, which averages with more temperate climates
					BSh<br>BWh			There are no specific data points about this power plant. Used the estimations of the coal-fired power plants. Used the maximum reported value considering that these climates require more water. In reality the values must be larger because this value comes from EIA Form 767 report from the USA, which averages with more temperate climates
					Cfa<br>Csb<br>Cwa<br>Cwb	[243]	2.472	There are no specific data points about this power plant. Used the estimations of the coal-fired power plants. Temperate climates are more similar to the data available in the datasets (Mostly USA and European power plants)
	Combined	Gas turbine + Heat Recovery	Wet Tower	Various crops	Aw	[244]	2.877	There are no specific data points about this power plant. Used the estimations of the gas-fired power plants. Maximum value because of climate in Africa is likely hotter and more humid than data available in the databases. This source is chosen because it provides data for both consumption and withdrawal

Technology	1st Category	2nd Category	3rd Category	Fuel	Climate	Source	Value [M³/MWh]	Assumption
	ICE	Gas-Engines	Dry cooling	Biogas	NA	Estimation based on the consumption.	0.324	Assumed similar ratio than cooling towers 70% in Dziegielewski & Bik [240].
Coal	Rankine	Steam Turbine	No cooling	Circulating Fluidized bed	NA	Estimation based on the consumption.	0.162	Considering that there is no freshwater consumption for the cooling system, assumed similar ratio than cooling towers 70% in Dziegielewski & Bik [240].
			Dry Cooling	Pulv - Subcritical	NA			
				Pulv - Supercritical	NA			
			Once through (Saline)	Circulating Fluidized bed	NA			
				Pulv - Subcritical	NA			
				Pulv - Ultrasupercritical	NA			
			Wet Tower	Circulating Fluidized bed	Af	240	3.785	Maximum value because of climate in Africa is likely hotter and more humid than data available in the databases
					Cwa	243	2.385	Temperate climates are more similar to the data available in the datasets (Mostly USA and European power plants)
				Pulverized - Subcritical	Af	242	4.542	Used the maximum reported value considering that these climates require more water. In reality, the real withdrawal could be higher because this value comes from EIA Form 767 report from the USA, which averages with more temperate climates
					BSh			Used the maximum reported value considering that these climates require more water. In reality the values must be larger because this value comes from EIA Form 767 report from the USA, which averages with more temperate climates
					BSk			
					BWh			
					Cfa	243	2.472	Temperate climates are very similar to the data available in the datasets (Mostly USA and European power plants)
					Cwa			
					Cwb			
	Combined	Steam Turbine	Wet Tower	IGCC	Aw	245	1.999	The maximum value due to climate in Africa must indicate a larger usage than in the case of the databases (data from USA and Europe). Used this value and not the maximum, because this source provides both consumption and withdrawal data
					Cwb	243	1.469	Temperate climates are more similar to the data available in the datasets (Mostly USA and European power plants).
	Geothermal		Wet Tower	Flash	Csb	243	0.068	

Technology	1st Category	2nd Category	3rd Category	Fuel	Climate	Source	Value [M^3/MWh]	Assumption
Geothermal		Steam Turbine	Dry Cooling	Binary - Dry cooled	NA	Estimation based on the consumption.	1.568	Considering that there is no freshwater consumption for the cooling system, assumed similar ratio than cooling towers 70% in Dziegielewski & Bik [240].
Natural Gas	Brayton	Gas turbine	No cooling	Natural Gas, Oil derivatives	NA	[246]	1.609	Use this value because it is the only one that reported both Consumption and Withdrawal, and is one of the three values reported in Meldrum
	Combined	Combined Cycle (CC)	Dry Cooling	Natural Gas	NA	[247]	0.038	Dry cooling, using the upper limit and the source that has both consumption and withdrawal
			Once through (Saline)	Natural Gas	NA			Same as dry cooling, considering that the water will be used in another requirement. Using the upper limit and the source that has both consumption and withdrawal
			Once through (Fresh)	Natural Gas	Aw	[247]	75.708	Maximum value because of climate in Africa is likely hotter and more humid than data available in the databases. This source is chosen because it provides data for both consumption and withdrawal.
					BWh			
			Wet Tower	Natural Gas	Am	[244]	2.877	
					Aw			
					BSh			
					BWh			
					Cwb	[243]	0,908	Temperate climates are more similar to the data available in the datasets (Mostly USA and European power plants)
	ICE	Gas-Engines	Dry Cooling	Natural Gas	NA	Estimation based on the consumption.	0.324	Assumed similar ratio than cooling towers 70% in Dziegielewski & Bik [240].
	Rankine	Steam Turbine	Once through (Saline)	Natural Gas	NA	[247]	0.038	There is no data for dry cooling for this type of power plant. Considered that is likely similar than CCGT, and that the water will be used in other requirement as there are no freshwater used in cooling. Using the upper limit and the source that has both consumption and withdrawal
			Once through (Fresh)	Natural Gas	Am	[246]	132.489	Maximum water intensity chosen. This source was used considering that must have both values for withdrawal and consumption.
					BWh			
			Wet Tower	Natural Gas	Am	[246]	4.580	
					BWh			
					Csa	[243]	4.561	Temperate climates are more similar to the data available in the datasets (Mostly USA and European power plants)
Oil	Brayton	Gas turbine	No cooling	Oil derivatives	NA	[246]	1.609	There is no data for these types of power plants, we consider the same as NG fired power plants

Technology	1st Category	2nd Category	3rd Category	Fuel	Climate	Source	Value [M^3/MWh]	Assumption
	Combined	Combined Cycle (CC)	Once through (Fresh)	HFO, NG	Aw	[247]	75.708	There is no data for these types of power plants, we consider the same as NG fired power plants. Maximum value because of climate in Africa is likely hotter and more humid than data available in the databases. This source is chosen because it provides data for both consumption and withdrawal.
			Wet Tower	LPG, Diesel	BWh	[244]	2.877	
	ICE	Diesel-engines	Dry Cooling	Oil derivatives	NA	Estimation based on the consumption.	0.324	Assumed similar ratio than cooling towers 70% in Dziegielewski & Bik [240].
				Syngas	NA			
	Rankine	Steam turbine	Once through (Saline)	Oil derivatives	NA	Estimation based on the consumption.	0.162	There are not data points for this technology. Considered the same as coal. Considering that there is no freshwater consumption for the cooling system, assumed similar ratio than cooling towers 70% in Dziegielewski & Bik [240].
			Once through (Fresh)	Oil derivatives	Csa	[243]	132.489	There is no data for these types of power plants. All the other authors consider them as coal power plants. Temperate climates are more similar to the data available in the datasets (Mostly USA and European power plants)
					BWh	[241]	189.271	There is no data for these types of power plants. All the other authors consider them as coal power plants. Considered the maximum value due to the climate. Source chosen based on availability of values for consumption and withdrawal by the same source
			Wet Tower	Oil derivatives	Aw	[242]	4.542	There is no data for these types of power plants. All the other authors consider them as coal power plants. Used the maximum reported value considering that these climates require more water. In reality, the real withdrawal could be higher because this value comes from EIA Form 767 report from the USA, which averages with more temperate climates
					BSh			
					BWh			
					Csa	[243]	2.472	There is no data for these types of power plants. All the other authors consider them as coal power plants. Temperate climates are more similar to the data available in the datasets (Mostly USA and European power plants). Used the median value
					Cwb			
Uranium	Nuclear	Steam Turbine	Once through (Saline)	Uranium	Na	Estimation based on the consumption.	0.379	There is no freshwater used for cooling, assumed similar ratio than cooling towers 30% in Dziegielewski & Bik [240].
waste heat	Rankine	Steam Turbine	Dry Cooling	Heat recovery	NA	[247]	0.038	There is no data for these types of power plants. The operation is similar to a combined cycle with gas. Freshwater use in saline cooling reported from literature. Dry cooling, using the upper limit and the source that has both consumption and withdrawal.
			Once through (Saline)	Heat recovery	NA			

Technology	1st Category	2nd Category	3rd Category	Fuel	Climate	Source	Value [M^3/MWh]	Assumption
			Wet Tower	Heat recovery	BSh	[244]	2.877	There is no data for these types of power plants. The operation is similar to a combined cycle with gas. Maximum value because of climate in Africa is likely hotter and more humid than data available in the databases. This source is chosen because it provides data for both consumption and withdrawal
					Cwa	[243]	0.908	There is no data for these types of power plants. The operation is similar to a combined cycle with gas. Temperate climates are more similar to the data available in the datasets (Mostly USA and European power plants). Median value used
					Cwb			
	ICE	Diesel engine	Dry cooling	Syngas	NA	Estimation based on the consumption.	0.324	Assumed similar ratio than cooling towers 70% in Dziegielewski & Bik [240].
Solar	PV	Flat	Rooftop	NA	Af	[248]	0.014	Roofs are not easily accessible and usually neglected. However, the withdrawal may be a bit larger than average due to the climate. It may require more water than what it has been reported in the datasets from temperate climates (US and EU).
					Am			
					Aw			
					BSh			
					BWh			
					Cfb	[249]	0.004	Roofs are not easily accessible and usually neglected. In particular, rainy climates do not require much additional cleaning.
					Csa			
					Csb			
					Cwa			
					Cwb			
			Land	NA	Af	[250]	0.098	Land installations are larger and require more maintenance and cleaning. Maximum value due to climate may require more water than what it has been reported in the datasets because the data points come from temperate climates (US and EU)
					Am			
					Aw			
					BSh			
					BSk			
					BWh			
					BWk			
					Cfb	[243]	0.023	Temperate climates are very similar to the data available in the datasets (Mostly USA and European power plants)
					Csb			
					Cwa			
					Cwb			
		Concentrated	Land	NA	BWh	[251]	0.295	Large need of water for cleaning. Used the maximum available
					BWk			
	CSP	Parabolic Trough	Dry Cooling	NA	BWh	Estimation based on the	0.757	Assumed similar ratio than cooling towers 70% in Dziegielewski & Bik [240]. We do not use the values reported, because they are the same as the consumptive use.
					BSh			
			Wet Tower	NA	BWh		10.275	
		Fresnel	Wet Tower	NA	CWb		5.408	

Technology	1st Category	2nd Category	3rd Category	Fuel	Climate	Source	Value [M^3/MWh]	Assumption
		Central Tower	Wet Tower	NA	BWh	consumption.	4.651	
Wind	Wind turbine	Onshore	No cooling	NA	NA	[243]	0.000	The wind power plant does not require water in operation. Only the water that is embedded in spare parts

Supplementary Table 6: Water consumption intensities for each power plant technology included in our assessment.

Technology	1st Category	2nd Category	3rd Category	Fuel	Climate	Source	Value [M^3/MWh]	Assumption
Biomass	Rankine	Steam Turbine	No cooling	Various crops	NA	[247]	0.114	As there are not data points for this particular type of technologies, considered to work similar to coal-fired power plants. Only one data point available in literature this type of technology and fuel.
			Once through (Fresh)	Various crops	Aw	[241]	1.136	There are not specific data points about this power plant. Used the estimations of the coal-fired power plants. Used the maximum reported value of the source with both consumption and withdrawal data, considering that these climates require more water. In reality, the real withdrawal could be higher because this value comes from the USA, which averages with more temperate climates
					BWh			
			Wet Tower	Various energy crops	Af	[242]	4.164	There are not specific data points about this power plant. Used the estimations of the coal-fired power plants. Used the maximum reported value considering that these climates require more water. In reality, the real withdrawal could be higher because this value comes from EIA Form 767 report from the USA, which averages with more temperate climates
					Am			
					Aw			
					BSh			There are not specific data points about this power plant. Used the estimations of the coal-fired power plants. Used the maximum reported value considering that these climates require more water. In reality the values must be larger because this value comes from EIA Form 767 report from the USA, which averages with more temperate climates
					BWh			
					Cfa	[243]	1.931	There are not specific data points about this power plant. Used the estimations of the coal-fired power plants. Temperate climates are very similar to the data available in the datasets (Mostly USA and European power plants)
					Csb			
					Cwa			
					Cwb			
	Combined	Gas turbine + Heat Recovery	Wet Tower	Various crops	Aw	[244]	1.022	There are no specific data points about this power plant. Used the estimations of the gas-fired power plants. Maximum value because of climate in Africa is likely hotter and more humid than data available in the databases. This source is chosen because it provides data for both consumption and withdrawal
	ICE	Gas-Engines	Dry cooling	Biogas	NA	[252]	0.227	There is no other source of these types of power plants, assumed to be similar than oil derivatives-fired power plants
Coal	Rankine	Steam Turbine	No cooling	Circulating Fluidized bed	NA	[247]	0.114	Only one data point available in literature this type of technology and fuel.
			Dry Cooling	Pulv - Subcritical	NA			

Technology	1st Category	2nd Category	3rd Category	Fuel	Climate	Source	Value [M^3/MWh]	Assumption
				Pulv - Supercritical	NA			
			Once through (Saline)	Circulating Fluidized bed	NA			
				Pulv - Subcritical	NA			
				Pulv - Ultrasupercritical	NA			
			Wet Tower	Circulating Fluidized bed	Af	240	2.650	Maximum value because of climate in Africa is likely hotter and more humid than data available in the databases
					Cwa	243	2.025	Temperate climates are more similar to the data available in the datasets (Mostly USA and European power plants)
				Pulverized - Subcritical	Af	242	4.164	Used the maximum reported value considering that these climates require more water. In reality, the real withdrawal could be higher because this value comes from EIA Form 767 report from the USA, which averages with more temperate climates
					BSh<br>BSk<br>BWh			Used the maximum reported value considering that these climates require more water. In reality the values can be higher because this value comes from EIA Form 767 report from the USA, which averages with more temperate climates
					Cfa<br>Cwa<br>Cwb	243	1.931	Temperate climates are very similar to the data available in the datasets (Mostly USA and European power plants). Median value used.
	Combined	Steam Turbine	Wet Tower	IGCC	Aw	253	1.582	The maximum value due to climate in Africa must indicate a larger usage than in the case of the databases (data from USA and Europe). Used this value and not the maximum, because this source provides both consumption and withdrawal data
					Cwb	243	1.211	Temperate climates are more similar to the data available in the datasets (Mostly USA and European power plants)
Geothermal	Geothermal	Steam Turbine	Wet Tower	Flash	Csb	243	0.042	
			Dry Cooling	Binary - Dry cooled	NA		1.098	
Natural Gas	Brayton	Gas turbine	No cooling	Natural Gas, Oil derivatives	NA	246	1.287	Use this value because it is the only one that reported both Consumption and Withdrawal, and is one of the three values reported in Meldrum
	Combined	Combined Cycle (CC)	Dry Cooling	Natural Gas	NA	247	0.026	Dry cooling, using the upper limit and the source that has both consumption and withdrawal

Technology	1st Category	2nd Category	3rd Category	Fuel	Climate	Source	Value [M³/MWh]	Assumption
			Once through (Saline)	Natural Gas	NA			Same as dry cooling, considering that the water will be used in another requirement. Using the upper limit and the source that has both consumption and withdrawal
			Once through (Fresh)	Natural Gas	Aw<br>BWh		0.416	Maximum value because of climate in Africa is likely hotter and more humid than data available in the databases. This source is chosen because it provides data for both consumption and withdrawal
			Wet Tower	Natural Gas	Am<br>Aw<br>BSh<br>BWh	244	1.022	
					Cwb	243	0.791	Temperate climates are more similar to the data available in the datasets (Mostly USA and European power plants)
	ICE	Gas-Engines	Dry Cooling	Natural Gas	NA	252	0.227	There is no other source of these types of power plants, asumed to be similar than oil derivatives-fired power plants
	Rankine	Steam Turbine	Once through (Saline)	Natural Gas	NA	247	0.025	There is no data for dry cooling for this type of power plant. Considered that is likely similar than CCGT, and that the water will be used in other requirement as there are no freshwater used in cooling. Using the upper limit and the source that has both consumption and withdrawal
			Once through (Fresh)	Natural Gas	Am<br>BWh	246	0.719	Maximum water intensity chosen. This source was used considering that must have both values for withdrawal and consumption.
			Wet Tower	Natural Gas	Am<br>BWh	246	3.653	
					Csa	243	2.744	Temperate climates are more similar to the data available in the datasets (Mostly USA and European power plants)
Oil	Brayton	Gas turbine	No cooling	Oil derivatives	NA	246	1.287	There is no data for these types of power plants, we consider the same as NG fired power plants
	Combined	Combined Cycle (CC)	Once through (Fresh)	HFO, NG	Aw	247	0.416	There is no data for these types of power plants, we consider the same as NG fired power plants. Maximum value because of climate in Africa is likely hotter and more humid than data available in the databases. This source is chosen because it provides data for both consumption and withdrawal
			Wet Tower	LPG, Diesel	BWh	244	1.022	
	ICE	Diesel-engines	Dry Cooling	Oil derivatives<br>Syngas	NA<br>NA	252	0.227	There is no other source of these types of power plants, assumed to be similar than oil derivatives-fired power plants
	Rankine	Steam turbine	Once through (Saline)	Oil derivatives	NA	247	0.108	There are no data points for this technology. Considered the same as coal. Only one data point available in literature this type of technology and fuel.
				Oil derivatives	Csa	243	0.757	There is no data for these types of power plants. All the other authors consider them as coal power plants. Temperate climates

Technolog y	1st Category	2nd Category	3rd Categor y	Fuel	Climate	Source	Value [M³/MWh]	Assumption
								are more similar to the data available in the datasets (Mostly USA and European power plants)
			Once through (Fresh)		BWh	241	1.136	There is no data for these types of power plants. All the other authors consider them as coal power plants. Considered the maximum value due to the climate. Source chosen based on availability of values for consumption and withdrawal by the same source
			Wet Tower	Oil derivatives	Aw<br>BSh<br>BWh	242	4.164	There is no data for these types of power plants. All the other authors consider them as coal power plants. Used the maximum reported value considering that these climates require more water. In reality, the real withdrawal could be higher because this value comes from EIA Form 767 report from the USA, which averages with more temperate climates
					Csa<br>Cwb	243	1.931	There is no data for these types of power plants. All the other authors consider them as coal power plants. Temperate climates are more similar to the data available in the datasets (Mostly USA and European power plants)
Uranium	Nuclear	Steam Turbine	Once through (Saline)	Uranium	Na	247	0.114	Same as dry cooling, considering that the water will be used in another requirement. Using the only value available
waste heat	Rankine	Steam Turbine	Dry Cooling	Heat recovery	NA	247	0.026	There is no data for these types of power plants. The operation is similar to a combined cycle with gas. Freshwater use in saline cooling reported from literature. Dry cooling, using the upper limit and the source that has both consumption and withdrawal
			Once through (Saline)	Heat recovery	NA		0.026	
			Wet Tower	Heat recovery	BSh	244	1.022	There is no data for these types of power plants. The operation is similar to a combined cycle with gas. Maximum value because of climate in Africa is likely hotter and more humid than data available in the databases. This source is chosen because it provides data for both consumption and withdrawal
					Cwa<br>Cwb	243	0.791	There is no data for these types of power plants. The operation is similar to a combined cycle with gas. Temperate climates are more similar to the data available in the datasets (Mostly USA and European power plants)
	ICE	Diesel engine	Dry cooling	Syngas	NA	252	0.227	There is no other source of these types of power plants, assumed to be similar than oil derivatives-fired power plants
Solar	PV	Flat	Rooftop	NA	Af<br>Am<br>Aw<br>BSh<br>BWh	Estimation based on withdrawal.	0.010	Assumed similar ratio of 70% of what is withdrawn from Dziegielewski & Bik [240]. We do not use the values reported, because they are the same as the consumptive use.

Technology	1st Category	2nd Category	3rd Category	Fuel	Climate	Source	Value [M^3/MWh]	Assumption
					Cfb		0.003	
					Csa			
					Csb			
					Cwa			
					Cwb			
			Land	NA	Af		0.069	
					Am			
					Aw			
					BSh			
					BSk			
					BWh			
					BWk			
					Cfb		0.016	
					Csb			
					Cwa			
					Cwb			
		Concentrated	Land	NA	BWh		0.207	
					BWk			
	CSP	Parabolic Trough	Dry Cooling	NA	BWh	[254]	0.530	As a desert, they should require to clean more often
					BSh			
			Wet Tower	NA	BWh	[255]	7.192	Maximum requirement as it is a hot desert and water evaporates easily
		Fresnel	Wet Tower	NA	CWb	[256]	3.785	Use the only value available for this type of power plants
		Central Tower	Wet Tower	NA	BWh	[257]	3.255	Maximum requirement as it is a hot desert and water evaporates easily
Wind	Wind turbine	Onshore	No cooling	NA	NA	[243]	0.000001	The wind power plant does not require water in operation. Only the water that is embedded in spare parts

Supplementary Table 7: Carbon Emission intensities for each power plant technology included in our assessment. The sources from this table were obtained from Nicholson & Garvin[258].

Technology	1st Category	2nd Category	3rd Category	Fuel	Climate	Source	Value [$g_{CO_{2e}}$/kWh]	Assumption
Biomass	Rankine	Steam Turbine	No cooling	Various crops	NA	259	42	There is no inventory only for the direct carbon footprint, using the median of the total life cycle for direct combustion. Direct combustion because as they do not have cooling, the power plant uses only biomass
			Once through (Fresh)	Various crops	Aw		170	There is no inventory only for the direct carbon footprint, using the median of the total life cycle for co-firing. In this case, as there is cooling, the power plant usually combines biomass with other fuels to obtain larger outputs
					BWh			
			Wet Tower	Various energy crops	Af			
					Am			
					Aw			
					BSh			
					BWh			
					Cfa			
					Csb			
					Cwa			
					Cwb			
	Combined	Gas turbine + Heat Recovery	Wet Tower	Various crops	Aw		40	There is no inventory only for the direct carbon footprint, using the median of the total life cycle of a Gasification power plant
	ICE	Gas-Engines	Dry cooling	Biogas	NA		95	There is no inventory only for the direct carbon footprint, using the median of the total life cycle
Coal	Rankine	Steam Turbine	No cooling	Circulating Fluidized bed	NA	260	1010	Chosen the median of the fluidized bed as there are no major differences with climate in the combustion
			Dry Cooling	Pulv - Subcritical	NA		1150	Chosen the median of the pulverized subcritical as there are no major differences with climate in the combustion
				Pulv - Supercritical	NA		880	Chosen the median of the pulverized supercritical as there are no major differences with climate in the combustion
			Once through (Saline)	Circulating Fluidized bed	NA		1010	Chosen the median of the fluidized bed as there are no major differences with climate in the combustion
				Pulv - Subcritical	NA		1150	Chosen the median of the pulverized subcritical as there are no major differences with climate in the combustion
				Pulv - Ultrasupercritical	NA		880	Chosen the median of the pulverized supercritical as there are no major differences with climate in the combustion
			Wet Tower	Circulating Fluidized bed	Af		1010	Chosen the median of the fluidized bed as there are no major differences with climate in the combustion
					Cwa			
					Af		1150	

Technology	1st Category	2nd Category	3rd Category	Fuel	Climate	Source	Value [$g_{CO_{2e}}$/kWh]	Assumption
				Pulverized - Subcritical	BSh<br>BSk<br>BWh<br>Cfa<br>Cwa<br>Cwb			Chosen the median of the pulverized subcritical as there are no major differences with climate in the combustion
	Combined	Steam Turbine	Wet Tower	IGCC	Aw<br>Cwb		790	Chosen the median of the IGCC as there are no major differences with climate in the combustion
Geothermal	Geothermal	Steam Turbine	Wet Tower	Flash	Csb	261	73	Chosen the median of the Flash technology as there are no major differences with climate
			Dry Cooling	Binary - Dry cooled	NA		3	Chosen the median of the EGS binary technology as there are no major differences with climate
Natural Gas	Brayton	Gas turbine	No cooling	Natural Gas, Oil derivatives	NA	262	605	Chosen the median of the combustion turbine as there are no major differences in the combustion due to climate (ongoing combustion and non-combustion)
	Combined	Combined Cycle (CC)	Dry Cooling	Natural Gas	NA		435	Chosen the median of the combined cycle technology as there are no major differences in the combustion due to climate (ongoing combustion and non-combustion)
			Once through (Saline)	Natural Gas	NA			
			Once through (Fresh)	Natural Gas	Aw<br>BWh			
			Wet Tower	Natural Gas	Am<br>Aw<br>BSh<br>BWh<br>Cwb			
	ICE	Gas-Engines	Dry Cooling	Natural Gas	NA		977	There is no information about this technology, chosen the maximum value of combustion turbine (ongoing combustion and non-combustion)
	Rankine	Steam Turbine	Once through (Saline)	Natural Gas	NA		650	There is no information about this technology, chosen the Q3 value of combustion turbine due to a lower efficiency (ongoing combustion and non-combustion)
			Once through (Fresh)	Natural Gas	Am<br>BWh			
			Wet Tower	Natural Gas	Am<br>BWh<br>Csa			

Technology	1st Category	2nd Category	3rd Category	Fuel	Climate	Source	Value [$g_{CO_{2e}}$/kWh]	Assumption
Oil	Brayton	Gas turbine	No cooling	Oil derivatives	NA	[263]	840	There is no data differentiation for this technology, and there is no difference between the whole cycle and just the direct component, used the median value
	Combined	Combined Cycle (CC)	Once through (Fresh)	HFO, NG	Aw			
			Wet Tower	LPG, Diesel	BWh			
	ICE	Diesel-engines	Dry Cooling	Oil derivatives	NA			
				Syngas	NA			
	Rankine	Steam turbine	Once through (Saline)	Oil derivatives	NA			
			Once through (Fresh)	Oil derivatives	Csa			
					BWh			
			Wet Tower	Oil derivatives	Aw			
					BSh			
					BWh			
					Csa			
					Cwb			
Uranium	Nuclear	Steam Turbine	Once through (Saline)	Uranium	Na	[264]	9,9	Used the median for the Pressurized Water reactor
waste heat	Rankine	Steam Turbine	Dry Cooling	Heat recovery	NA	[262]	435	Chosen the median of the combined cycle technology as they are practically a separation of the combined technology, and there are no major differences in the combustion due to climate (ongoing combustion and non-combustion)
			Once through (Saline)	Heat recovery	NA			
			Wet Tower	Heat recovery	BSh			
					Cwa			
					Cwb			
	ICE	Diesel engine	Dry cooling	Syngas	NA		977	There is no information about this technology, chosen the maximum value of combustion turbine (ongoing combustion and non-combustion)
Solar	PV	Flat	Rooftop	NA	Af	[265]	56	Used the median value for Roof-mounted because it would be extremely difficult to assess the technology per installation only using Satellite images.
					Am			
					Aw			
					BSh			
					BWh			
					Cfb			
					Csa			
					Csb			

Technology	1st Category	2nd Category	3rd Category	Fuel	Climate	Source	Value [g_{CO2e}/kWh]	Assumption
					Cwa			
					Cwb			
			Land	NA	Af		68	Used the median value for Ground-mounted because it would be extremely difficult to assess the technology per installation only using Satellite imaging
					Am			
					Aw			
					BSh			
					BSk			
					BWh			
					BWk			
					Cfb			
					Csb			
					Cwa			
					Cwb			
		Concentrated	Land	NA	BWh		20	There is no data for concentrated, but considering that there is less pv material used in these power plants, the minimum value for Ground-mounted is used
					BWk			
	CSP	Parabolic Trough	Dry Cooling	NA	BWh	266	10	Median value for the PT technology as there are no differences in the type of cooling.
					BSh			
			Wet Tower	NA	BWh			
		Fresnel	Wet Tower	NA	CWb		10	Median value for the PT technology as there are no values exclusively for Linear Fresnel, and there are no differences in the type of cooling
		Central Tower	Wet Tower	NA	BWh		31	Median value for the CT technology as there are no differences in the type of cooling
Wind	Turbine	Onshore	No cooling	NA	NA	267 Appendix J	0.74	Median value considering not a lot of change in technology due to climate
Hydropower	Reservoir	NA	NA	NA	NA	268 Appendix G	0.55	Median value of the non-combustion of the reservoir
	ROR	NA	NA	NA	NA		1.90	Median value of the non-combustion of the ROR

Supplementary Table 8: Electricity produced (GWh) and Number of power plants for the years 2024 and 2025, for INVENTORY as established at the end of 2024, as well as INVENTORY with 2024 and 2025 real world data as analyzed at the beginning of 2026

	INVENTORY		INVENTORY with 2024 and 2025 real world data		difference (in %)	
Year	2024	2025	2024	2025	2024	2025
Electricity produced (GWh)						
oil	65,815	67,141	59,521	61,729	10	8
coal	298,565	300,203	289,338	300,055	3	0
natural gas	581,317	591,674	575,108	588,933	1	0
waste heat	3,675	4,367	3,675	5,067	0	-16
nuclear	31,304	31,304	13,668	13,668	56	56
biomass	4,406	4,775	4,531	4,855	-3	-2
sun	16,617	17,564	13,398	16,752	19	5
wind	24,087	24,501	20,936	22,080	13	10
geothermal	9,086	11,650	7,736	9,592	15	18
hydro	187,479	197,682	172,625	197,043	8	0
Sum all fuel types	1,222,351	1,250,861	1,160,537	1,219,774	5	2
Number of power plants						
oil	1,050	1,051	1,046	1,050	0	0
coal	51	53	50	55	2	-4
natural gas	376	381	371	382	1	0
waste heat	15	16	15	16	0	0
Nuclear	2	2	1	1	50	50
biomass	122	125	125	128	-2	-2
sun	405	413	406	426	0	-3
wind	110	112	108	113	2	-1
geothermal	11	13	10	13	9	0
hydro	577	595	578	601	0	-1
Sum all fuel types	2,719	2,761	2,710	2,785	0	-1

Supplementary references

1. WRI. *Global Power Plant Database, Https://Datasets.Wri.Org/Dataset/Globalpowerplantdatabase*. (2021).
2. Peters, R., Berlekamp, J., Tockner, K. & Zarfl, C. RePP Africa – a georeferenced and curated database on existing and proposed wind, solar, and hydropower plants. *Sci Data* **10**, 16 (2023).
3. IEA. World Energy Balances. IEA (2025).
4. Gerbens-Leenes, P. W., Vaca-Jiménez, S. D., Holmatov, B. & Vanham, D. Spatially distributed freshwater demand for electricity in Africa. *Environmental Science: Water Research & Technology* **10**, 1795–1808 (2024).
5. IEA. *Renewable Energy and Energy Efficiency Development Plan 2015-2030*. (2016).
6. République Algérienne Démocratique et Populaire. *Contribution Prévue Déterminée Au Niveau National CPDN - Algerie*. (2015).
7. Republic of Angola. *Nationally Determined Contribution of Angola*. (2021).
8. Ministére du Cadre de Vie et du Développement Durable & UNDP. *Contribution Determinee Au Niveau National Actualisee Du Benin Au Titre de L'Accord de Paris*. (2021).
9. IRENA. *Renewables Readiness Assessment: Botswana*. (2021).
10. Republic of Botswana. *Botswana Intended Nationally Determined Contribution*. (2016).
11. IRENA. *Renewables Readiness Assessment: Burkina Faso*. (2023).
12. Repúblique du Burkina Faso. *Contribution Determinée Au Niveai National (CDN) Du Burkina Faso 2021-2025*. (2021).
13. Banque de la République du Burundi. *Production d'Energie Electrique*. https://www.brb.bi/node/204 (2024).
14. Republique du Burundi. *Contribution Determinee Au Niveau National 2020*. (2021).
15. AfDB. *Country Priority Plan and Diagnostic of the Electricity Sector: Cameroon*. (2021).
16. AFC & FINNFUND. *The Link between Power Investments, Incomes and Jobs in Cape Verde*. (2018).
17. Assembleia Nacional da República de Cabo Verde. *Resolução Nº 39/2019 – Plano Diretor Do Setor Elétrico – 2018-2040. 39/2019* (Cabo Verde, 2019).
18. EMBER. Yearly Electricity Data. (2025).
19. Ministere de L'Environnement et du Developpement Durable. *Contribution Determinee Au Niveau National (CDN) Version Revisee*. (2021).
20. Republique du Tchad. *Mise a Jour de La Contribution Determinee Nationale (CDN)*. (2021).
21. Ministere de L'Environnement, du D. D. et du B. du C. *Contribution Determinee Au Niveau National (CDN) de La Republique Du Congo*. (2021).
22. Republique Democratique du Congo. *Contribution Déterminée a l'échelle Nationale Révisée*. (2021).
23. Ministére de l'Environnement et du Développement Durable, UNEP, UNDP, & GIZ. *Contributions Déterminées Au Niveau National (CDN) de La Cote d'Ivoire*. (2022).
24. Ministére de l'Habitat, de l'Urbanisme et de l'Environnement. *Contribution Prévue Déterminée Au Niveau National de La République de Djibouti*. (2015).
25. Arab Republic of Egypt. *Egypt's Second Updated Nationally Determined Contributions*. (2023).
26. IRENA. *Renewable Energy Outlook: Egypt*. (2018).
27. República de Guinea Ecuatorial. *CONTRIBUCIONES DETERMINADAS A NIVEL NACIONAL (CDN)*. (2022).
28. The State of Eritrea. *Nationally Determined Contributions (NDCs) Report to UNFCCC*. (2018).
29. Federal Democratic Republic of Ethiopia. *Updated Nationally Determined Contribution*. (2021).
30. Repúblique Gabonaise. *Seconde Contribution Déterminée Au Niveau National*. (2022).
31. Climate Change Secretariat. *Second Nationally Determined Contribution of The Gambia*. (2021).

32. MESTI. *Updated Nationally Determined Contribution under the Paris Agreement (2020 - 2030)*. (2021).
33. République de Guinée. *Contribution Déterminée Au Niveau National (CDN) de La République de Guinée*. (2021).
34. Republic of Guinea-Bissau. *UPDATED NATIONALLY DETERMINED CONTRIBUTION IN THE FRAMEWORK OF THE PARIS CLIMATE AGREEMENT*. (2021).
35. Ministry of Energy and Petroleum & State department for energy. *Kenya Energy & Investment Plan 2023-2050*. (2023).
36. Ministry of Environment and Forestry. *Kenya's Updated Nationally Determined Contribution (NDC)*. (2020).
37. Ministry of Environment and Forestry. *Second Nationally Determined Contributions of Lesotho*. (2024).
38. Environment Protection Agency of the Republic of Liberia. *Liberia's Revised Nationally Determined Contribution (NDC)*. (2021).
39. Libyan Transitional Government & Ministry of Electricity and Renewable Energy. *National Plan for Developing the Renewable Energy in Libya*. (2012).
40. République de Madagascar. *DEUXIÈME CONTRIBUTION DÉTERMINÉE AU NIVEAU NATIONAL DE LA RÉPUBLIQUE DE MADAGASCAR AU TITRE DE L'ACCORD DE PARIS*. (2022).
41. Republic of Malawi. *Updated Nationally Determined Contributions*. (2021).
42. IRENA. *Renewables Readiness Assessment: Mali*. (2019).
43. Ministére de l'Environnement, de l'Assainissement et du Développement Durable. *Contribution Détertminée Au Niveau National Révisée*. (2021).
44. Economic Development board Mauritius. Renewable Energy. https://edbmauritius.org/renewable-energy (2024).
45. Republic of Mauritius. *UPDATE OF THE NATIONALLY DETERMINED CONTRIBUTION OF THE REPUBLIC OF MAURITIUS*. (2021).
46. Royaume du Maroc. *Contribution Déterminée Au Niveau National - Actualisée (CDN - Maroc)*. (2021).
47. Republic of Mozambique. *Update of the First Nationally Determined Contribution to the United Nations Framework Convention on Climate Change Mozambique. Period 2020 - 2025*. (2021).
48. Ministry of Environment, F. and T. *Namibia's Nationally Determined Contribution: Second Update*. (2023).
49. Republique du Niger. *Contribution Déterminée Au Niveau National*. (2021).
50. The Federal Government of Nigeria. *Nigeria's Nationally Determined Contribution*. (2021).
51. Republic of Rwanda. *Updated Nationally Determined Contribution*. (2020).
52. República Democrática de Sao Tomé e Príncipe. *Sao Tomé e Príncipe Nationally Determined Contributions (NDC-STP) Updated*. (2021).
53. Republique du Senegal. *Contribution Déterminée Au Niveau National Du Senegal*. (2020).
54. Republic of Seychelles. *Seychelles' Updated Nationally Determined Contribution*. (2021).
55. The Republic of Sierra Leone. *Updated Nationally Determined Contribution*. (2021).
56. The Federal Republic of Somalia. *Updated Nationally Determined Contribution (NDC)*. (2021).
57. IRENA. *Renewable Energy Prospects South Africa*. (2020).
58. Republic of South Africa. *First Nationally Determined Contribution Under the Paris Agreement*. (2021).
59. The Republic of South Sudan. *South Sudan's Second Nationally Determined Contribution*. (2021).
60. Republic of the Sudan. *First Nationally Determined Contribution under the Paris Agreement*. (2021).
61. The United Republic of Tanzania. *Nationally Determined Contribution*. (2021).
62. République Togolaise. *Contributions Detérminées Au Niveai National (CDN) Revisées*. (2021).

63. République Tunisienne. *Contribution Déterminée Au Niveau National (CDN) Actualisée Tunisie*. (2021).
64. Ministry of Water and Environment. *Updated Nationally Determined Contribution (NDC)*. (2022).
65. Republic of Zambia. *Nationally Determined Contribution (NDC) of Zambia for the Timeframe 2015-2030*. (2020).
66. Government of Zimbabwe. *Zimbabwe Revised Nationally Determined Contribution*. (2021).
67. GemWiki. Global Energy Monitor Wiki. *Global Energy Monitor* https://www.gem.wiki/Main_Page (2024).
68. Power Technology. Data Insights. https://www.power-technology.com/data-insights/?selected_facets%5B0%5D=categories_str%3AData Insights&search_sort=sort_by_date_desc (2024).
69. Wikipedia. The Free Encyclopedia. https://en.wikipedia.org/wiki/Main_Page (2024).
70. winenergysa. Lubango. https://winenergysa.com/index.php?option=com_k2&view=item&id=226:central-elétrica-do-lubango-huíla-em-angola&Itemid=225&lang=en (2020).
71. KENSCO. Project collaboration with UNDP Burukina Faso 2022 – KENS.CO;LTD. https://www.kensco.jp/undp-project-in-burkina-faso/ (2022).
72. The WindPower. Wind farms - Online access - The Wind Power - Wind energy Market Intelligence. *Wind Energy Market Intelligence* https://www.thewindpower.net/windfarms_list_en.php?country=CV (2024).
73. Africa Energy Portal. Diesel generator sets to power Bangui in Central African Republic. https://africa-energy-portal.org/news/diesel-generator-sets-power-bangui-central-african-republic (2019).
74. Africa Energy Portal. Central African Republic. https://africa-energy-portal.org/aep/country/central-african-republic (2024).
75. afrik21. Sarh une centrale solaire de 30 MW alimentera les populations. https://www.afrik21.africa/tchad-a-sarh-une-centrale-solaire-de-30-mwc-alimentera-les-populations-et-la-nstt/ (2022).
76. LaRoucci Tesla Chad. Electric Power Thermal. https://laroucciinternational.org/electric-power-thermal/ (2022).
77. Zoomeco. RDC : infrastructures, le barrage de Busanga construit par SICOMINES enfin opérationnel | Zoom Eco. *A la Une* https://zoom-eco.net/a-la-une/rdc-infrastructures-le-barrage-de-busanga-construit-par-sicomines-enfin-operationnel/#google_vignette (2022).
78. Ilvief. New Chemical Factory-Kima. https://ilvief.gr/portfolio/kima-aswan/#.
79. African Energy. Koysha (Gilgel Gibe IV) Hydro. https://www.africa-energy.com/database/koysha-gilgel-gibe-iv-hydro (2024).
80. ESI Africa. Ghana’s hybrid power plant - a successful mix of solar and hydro. https://www.esi-africa.com/renewable-energy/tour-ghanas-hybrid-power-plant-a-successful-mix-of-solar-and-hydro/ (2023).
81. ChiniMandi. Komenda sugar mill to generate electricity - ChiniMandi. https://www.chinimandi.com/komenda-sugar-mill-to-generate-electricity/ (2020).
82. afrik21. Kaleo solar pv plant goes into operation. https://www.afrik21.africa/en/ghana-kaleo-solar-pv-plant-goes-into-operation/ (2022).
83. Norgold. Nordgold Launches New Power Plant at its Lefa Gold Mine in Guinea • Nordgold. *News* https://nordgold.com/media/news/nordgold-launches-new-power-plant-at-its-lefa-gold-mine-in-guinea/ (2022).
84. Nvision. Renewable Energy Investments. *Our Investments* https://nvisionenergy.com/ (2024).
85. Capital Business. Tata Chemicals Magadi Eyes Innovative Climate Smart Solutions for Growth - Capital Business. https://www.capitalfm.co.ke/business/2023/02/tata-chemicals-magadi-eyes-innovative-climate-smart-solutions-for-growth/ (2022).

86. KTDA. Tea Farmers to Set Up 1.8 Mw Power Plant in Embu County - KTDA. https://ktdateas.com/tea-farmers-to-set-up-1-8-mw-power-plant-in-embu-county/.
87. Liberia Electricity Corporation. Commissioning & Dedication of the Newly Constructed Congo Town Substation – Liberia Electricity Corporation. https://lecliberia.com/news-releases/commissioning-dedication-of-the-newly-constructed-congo-town-substation/ (2022).
88. Open Infrastructure Map. Libya Power Plants. https://openinframap.org/stats/area/Libya/plants (2024).
89. Presidence. 24.01.21 - Inauguration de la centrale hybride solaire à Tanambao Verrerie à Toamasina, Région Atsinanana. *Accueil* https://www.presidence.gov.mg/velirano-2-l-energie-et-l-eau-pour-tous/24-01-21-inauguration-de-la-centrale-hybride-solaire-a-tanambao-verrerie-a-toamasina-region-atsinanana.html (2021).
90. L'Express de Madagascar. Centrale solaire Andranotakatra - La première phase du projet inaugurée. https://lexpress.mg/08/06/2021/centrale-solaire-andranotakatra-la-premiere-phase-du-projet-inauguree/ (2021).
91. ESI Africa. New photovoltaic plant gives Madagascar energy boost. https://www.esi-africa.com/east-africa/new-solar-pv-plant-gives-madagascar-energy-boost/ (2023).
92. AfDB. Madagascar - Nosy be renewable energy power project - Project Preparation Grant Proposal - SEFA Appraisal Report. https://www.afdb.org/fr/documents/madagascar-nosy-be-renewable-energy-power-project-project-preparation-grant-proposal-sefa-appraisal-report (2020).
93. Africa Energy Portal. MADAGASCAR: a 1.8 MWp solar PV power plant goes into operation in Antalaha. https://africa-energy-portal.org/news/madagascar-18-mwp-solar-pv-power-plant-goes-operation-antalaha (2023).
94. Eco Austral. Cap Sud dote la ville minière d'Ilakaka d'une centrale solaire hybride. https://ecoaustral.com/cap-sud-dote-la-ville-miniere-dilakaka-dune-centrale-solaire-hybride-2/ (2021).
95. Water Power Magazine. Farahantsana hydroelectric plant inaugurated in Madagascar - International Water Power. *News* https://www.waterpowermagazine.com/news/farahantsana-hydroelectric-plant-inaugurated-in-madagascar-10416382/ (2022).
96. Wärtsilä. Wärtsilä signs Decarbonisation Agreement for Madagascan power plant. https://www.wartsila.com/media/news/19-08-2024-wartsila-signs-decarbonisation-agreement-for-madagascan-power-plant-3483653 (2024).
97. Archipels. Green Energy Solutions installs 3 hybrid power plants in the Sava region. *ECONOMIE CIRCULAIRE ET ENVIRONNEMENT DANS L'OCEAN INDIEN* https://www.lejournaldesarchipels.com/2021/11/22/green-energy-solutions-installs-3-hybrid-power-plants-in-the-sava-region/?lang=en (2021).
98. Zutari. Toilara hybrid power. *Zutari projects* https://www.zutari.com/project/toliara-hybrid-power-project/ (2020).
99. EBSCO. Madagascar's energy production capacity | EBSCO Research Starters. https://www.ebsco.com/research-starters/power-and-energy/madagascars-energy-production-capacity (2024).
100. EGENCO. Solar Power for Kiloma and Chizumulu Islands. (2021).
101. CleanTechnica. 21 MW Nkhotakota Solar PV Plant In Malawi Energized - CleanTechnica. https://cleantechnica.com/2023/03/04/21-mw-nkhotakota-solar-pv-plant-in-malawi-energized/ (2023).
102. Leads2Business. Project: Mulunguzi Hydro-Power Plant, Malawi | L2B. https://www.l2b.co.za/Project/Mulunguzi-Hydro-Power-Plant-Malawi/20565 (2024).
103. afrik21. Mali Hybrid Solar Power Plant Goes into operation at the Nampala Mine. https://www.afrik21.africa/en/mali-hybrid-solar-power-plant-goes-into-operation-at-the-nampala-mine/ (2022).
104. CEB. CEB: POWER STATIONS. https://ceb.mu/our-activities/power-stations/2mw-solar-pv-farm-henrietta.

105. Ministry of Energy and Utilities. Energy Sector. https://publicutilities.govmu.org/Pages/Energy%20Sector/EnergySector.aspx (2024).
106. Wärtsilä. Nigeria's industry is rising to the twin challenge of decarbonisation and energy security. https://www.wartsila.com/zaf/media/local-news/11-08-2023-nigeria-s-industry-is-rising-to-the-twin-challenge-of-decarbonisation-and-energy-security (2023).
107. ESI Africa. Katsina wind farm makes headway for Nigeria's abandoned projects - ESI-Africa.com. https://www.esi-africa.com/industry-sectors/generation/katsina-wind-farm-makes-headway-for-nigerias-abandoned-projects/ (2021).
108. The Guardian Nigeria. Geometric Power Plant to add 188MW to national grid | The Guardian Nigeria News - Nigeria and World News — Business — The Guardian Nigeria News – Nigeria and World News. https://guardian.ng/business-services/geometric-power-plant-to-add-188mw-to-national-grid/ (2023).
109. The Cable. Dangote Cement plant to commence operations in Edo | TheCable. https://www.thecable.ng/dangote-cement-plant-to-commence-operations-in-edo/ (2021).
110. Coca-Cola HBC. NBC Unveils Plans to Transition Operations to Renewable Energy. https://ng.coca-colahellenic.com/en/media/news/sustainability/2020/renewable-energy-media-conference (2022).
111. Elektron Energy. Alausa Network Limited. https://www.elektronenergy.com/alausa-power-limited/ (2019).
112. Premium Times. Kano 10 megawatts hydropower plant completed, begins test run. https://www.premiumtimesng.com/news/more-news/574996-kano-10-megawatts-hydropower-plant-completed-begins-test-run.html (2023).
113. REG. Rubavu: Shema Gas Methane power plant nears completion. https://www.reg.rw/media-center/news-details/news/rubavu-shema-gas-methane-power-plant-nears-completion/ (2021).
114. REG. Power Plant. https://www.reg.rw/what-we-do/generation/power-plant/ (2024).
115. AfDB. SPN - São Tomé and Príncipe - EPC, South PV Plant of Santo Amaro - ETISP | African Development Bank Group. https://www.afdb.org/en/documents/spn-sao-tome-and-principe-epc-south-pv-plant-santo-amaro-etisp (2022).
116. Urbasolar. Cococim Industries s'est associe a Urbasolar. https://urbasolar.com/references/sococim-2/ (2019).
117. Wärtsilä. Wärtsilä power plant upgrade and extension will enable production expansion for largest gold mine in Senegal. https://www.wartsila.com/media/news/16-08-2022-wartsila-power-plant-upgrade-and-extension-will-enable-production-expansion-for-largest-gold-mine-in-senegal-3141522 (2022).
118. ESI Africa. Seychelles upping its renewable energy electricity capacity -. https://www.esi-africa.com/industry-sectors/generation/seychelles-upping-its-renewable-energy-electricity-capacity/ (2022).
119. Serengeti Energy. Serengeti Energy's Solar Power Project on course to be the first grid connected commercial power project in Sierra Leone. https://www.serengetienergy.com/serengeti-energys-solar-power-project-on-course-to-be-the-first-grid-connected-independent-power-project-in-sierra-leone/ (2022).
120. FTL. Puntland and UAE Jointly Fund a Bosaso Power Plant - Somali News today Live. https://www.ftlsomalia.com/puntland-and-uae-jointly-fund-a-bosaso-power-plant/ (2020).
121. Kruisvallei Hydro. Kruisvallei Hydro. https://kruisvalleihydro.energy/ (2024).
122. sappi. Ngodwana Energy biomass power plant | Sappi Global. https://www.sappi.com/nl/ngodwana-energy-biomass-power-plant.
123. BusinessLive. Mpact focuses on green packaging, while it gears up to generate power for grid. *BusinessDay* https://www.businesslive.co.za/bd/companies/industrials/2022-08-14-mpact-focuses-on-green-packaging-while-it-gears-up-to-generate-power-for-grid/ (2022).
124. Sowetan. Solar project at CT office park has potential to save health department millions in electricity bills. https://www.sowetanlive.co.za/news/south-africa/2023-04-27-solar-project-at-ct-office-park-has-potential-to-save-health-department-millions-in-electricity-bills/ (2023).

125. Mass group Holding. AlShamal Cement Factory – Atbara/ Sudan. http://www.massgroupholding.com/English/Newsdetail-unit_2.aspx?jimare=13&title=AlShamal%20Cement%20Factory%20%E2%80%93%20Atbara/%20Sudan&cor=3.
126. atmosfair. Tanzania: Power generation from coconut wood residues on Mafia Island - atmosfair. https://www.atmosfair.de/en/climate-protection-projects/biogas-biomass/tanzania-power-generation-from-coconut-wood-residues-on-mafia-island/ (2020).
127. IRENA. Togo Inaugurates 50MW Solar Plant Financed Under IRENA-ADFD Facility. https://www.irena.org/news/pressreleases/2021/Jun/Togo-Inaugurates-50MW-Solar-Plant-Financed-Under-IRENA-ADFD-Facility (2021).
128. African Energy. Uganda: Muyembe hydroelectric power plant starts operating. https://www.africa-energy.com/live-data/article/uganda-muyembe-hydroelectric-power-plant-starts-operating (2023).
129. LSDMFEZ. *Lusaka South Multi Facility Economic Zone Limited's Quaterly Press Statement*. (2021).
130. GlobalData. Explore Global Construction Projects | GlobalData. *Report Store* https://www.globaldata.com/store/industry/construction-projects/?selected_facets%5B0%5D=report_type_str%3AProduct Insights (2024).
131. Angola Energy. HYDRO ENERGY | Angola Energy 2025. *Hydro Energy: Small and Medium Hydro Power Plants* https://angolaenergia2025.gestoenergy.com/en/conteudo/hydro-energy (2024).
132. allAfrica. Angola Re-Inaugurates Mabubas Hydro-Electric Dam. https://allafrica.com/view/group/main/main/id/00016874.html (2012).
133. INRH. Instituto Nacional de Recursos Hídricos. *Portal SNIRHA* http://www.inrh.gv.ao/portal/snirha (2024).
134. OpenStreetMap. Soyo LNG Power Plant - Open Infrastructure Map. https://openinframap.org/stats/area/Angola/plants/923201220.
135. JIICA. Feasibility Study Site. in *The Preparatory Survey on Rural Electrification Development Works in the Republic of Angola* (2011).
136. UNIDO & ICSHP. *World Small Hydropower Development Report 2022. Western Africa*. (2022).
137. African Energy. Benin: Government embarks on 140MW project in development zone | African Energy. *Live Data* https://www.africa-energy.com/live-data/article/benin-government-embarks-140mw-project-development-zone (2022).
138. ECOWREX. ECOWAS Observatory for Renewable Energy and Energy Efficiency. *Energy Generators* www.ecowrex.org/resources/energy_generators (2024).
139. Factor This. Benin seeks studies of three hydro projects on Oueme River. *Project Development* https://www.renewableenergyworld.com/energy-business/new-project-development/benin-seeks-studies-of-three-hydro-projects-on-oueme-river/#gref (2014).
140. Hydrocarbures. Botswana : Gaborone compte se doter d'une centrale à charbon de 300 MW au plus tard 2019. https://www.agenceecofin.com/electricite/1208-12905-botswana-gaborone-compte-se-doter-d-une-centrale-a-charbon-de-300-mw-au-plus-tard-2019 (2013).
141. AfDB. Multinational - Projet amélioration de l'environnement propice à la gestion durable et équitable des ressources en eau - MapAfrica - Groupe de la Banque africaine de développement. https://mapafrica.afdb.org/fr/projects/46002-P-Z1-EA0-012 (2021).
142. NHPC. Nachtigal Hydro Power Company. https://nhpc.cm/home/ (2023).
143. Cameroon tribune. Centrale thermique à gaz de Limbe : ça se précise. *Economie* https://www.cameroon-tribune.cm/article.html/42293/en.html/centrale-thermique-gaz-de-limbe-ca-se (2021).
144. Tchatat, G. *Contribution a La Preparation Du Rapport National Pour La Formulation Du Livre Blanc Regional Sur l'acces Universel Aux Services Energetiques Integrant Le Developpement Des Energies Renouvelables et de l'efficacite Energetique*. (2014).

145. Open Infrastructure Map. Centrale hydroélectrique de la Tshopo. https://openinframap.org/stats/area/Democratic%20Republic%20of%20the%20Congo/plants/176980014.
146. World Bank & Economic Consulting Associates. *Scaling up Electricity Access in the Democratic Republic of the Congo*. https://documents1.worldbank.org/curated/en/251311588058290852/txt/Scaling-up-Electricity-Access-in-the-Democratic-Republic-of-the-Congo.txt (2017).
147. Zoomeco. Perenco plans construction of 250 MW gas plant. http://en.zoom-eco.net/breaking-news/drc-perenco-plans-construction-of-250-megawatt-gas-plant/ (2023).
148. Karpowership. Powership Project Cote D'Ivoire. https://karpowership.com/project-ivory-coast (2023).
149. Factor This. Singrobo hydropower plant wins Power Deal of the Year award. https://www.renewableenergyworld.com/energy-business/energy-finance/singrobo-hydropower-plant-wins-power-deal-of-the-year-award/ (2024).
150. World Bank Group. *Djibouti - Geothermal Power Generation Project*. https://documents.worldbank.org/en/publication/documents-reports/documentdetail/124881468246587008/djibouti-geothermal-power-generation-project (2013).
151. Energy Monitor. Power plant profile: Ghoubet Djibouti Wind Farm, Djibouti. https://www.energymonitor.ai/data-insights/power-plant-profile-ghoubet-djibouti-wind-farm-djibouti/ (2023).
152. EBRD. Scatec Benban V (Zafarana Solar Project). https://www.ebrd.com/work-with-us/projects/psd/scatec-benban-v-zafarana-solar-project.html (2017).
153. Energy Monitor. Energy Monitor - Inside the global transition to net zero. *Renewables* https://www.energymonitor.ai/ (2024).
154. Gas to Power Journal. Wärtsilä to boost capacity of Borg El Arab power plant in Egypt - Gas To Power Journal. https://www.gastopowerjournal.com/projectsafinance/item/12630-waertsilae-to-boost-capacity-of-borg-el-arab-power-plant-in-egypt (2022).
155. Ethiopian Electric Power. AYESHA II WPP PROJECT. https://www.eep.com.et/?project=aysha-wind-project.
156. Wärtsilä. Wärtsilä signs Concession Agreement to develop, supply, construct, operate and maintain major 120 MW power plant project in Gabon. https://www.wartsila.com/media/news/23-09-2021-wartsila-signs-concession-agreement-to-develop-supply-construct-operate-and-maintain-major-120-mw-power-plant-project-in-gabon-2979375 (2021).
157. AEP. Gambia: Barrow commissions $28.4M Brikama power plant | Africa Energy Portal. *News* https://africa-energy-portal.org/news/gambia-barrow-commissions-284m-brikama-power-plant (2021).
158. Newmont Corporation. *Power Plants Mines*. (2015).
159. allAfrica. Ghana: Mim Timber Factory Gets Gh¢6.5 Million 1d1f Bailout - allAfrica.com. https://allafrica.com/stories/202009170453.html (2020).
160. Africa Intelligence. IVORY COAST/GHANA: TDA LOAN FOR ESIAMA STATION. *Africa Energy Intelligence* https://www.africaintelligence.com/west-africa/2000/01/12/tda-loan-for-esiama-station,75010-art (2000).
161. Synergy Real Property. Ghana undertake construction of new power plant next year. https://www.synergyrealproperty.org/ghana-undertake-construction-new-power-plant-next-year/.
162. Genser Energy. Operations | Genser Energy. https://www.genserenergy.com/operations/ghana/wassa-plant/ (2024).
163. Graphic Online. 3 Small hydro dams to be built on Ankobra River - Graphic Online. https://www.graphic.com.gh/news/general-news/3-small-hydro-dams-to-be-built-on-ankobra-river.html (2015).

164. IHA. Ghana: assessing the sustainability of new hydropower sites. https://www.hydropower.org/blog/ghana-assessing-the-sustainability-of-new-hydropower-sites (2015).
165. Upwind Ayitepa. The Project Overview. https://upwindayitepa.com/project/overview (2024).
166. Mining Technology. Projects. https://www.mining-technology.com/projects/kiniero-gold-project-the-republic-of-guinea/ (2023).
167. ESI Africa. Agreement for 100 MW wave power facility in Guinea - ESI-Africa.com. https://www.esi-africa.com/top-stories/agreement-for-100-mw-wave-power-facility-in-guinea/ (2013).
168. TARDA. Solar Power Development In Masinga And Kiambere – Tana and Athi Rivers Development Authority (TARDA). https://tarda.go.ke/portfolio/solar-power-development-in-masinga-and-kiambere/ (2021).
169. Leads2Business. Project Tindinyo Falls HPP/27572. https://www.l2b.co.za/Project/Tindinyo-Falls-HPP/27572 (2024).
170. CEMNET. Kenya: National Cement to build 15MW power plant from International Cement Review. *2014* https://www.cemnet.com/News/story/155634/kenya-national-cement-to-build-15mw-power-plant.html.
171. Frontier Energy. Nithi Hydro Power Project. https://frontier.dk/nithi-mini-hydro-power-project/.
172. EPRA. *Power Undertaking Register*. (2022).
173. Tell Team. Small scale tea farmers to build $27 million hydro power plant. https://tell.co.ke/small-scale-tea-farmers-to-build-27-million-hydro-power-plant/ (2020).
174. eOpportunities Kenya. Lower Ewaso Ng'iro South River Multipurpose Dam Development Project (LENSDEP). https://opportunities.invest.go.ke/opportunities/opportunity/lower-ewaso-ngiro-south-river-multipurpose-dam-development-project-lensdep.
175. Sanima Engineering. Kipsonoi Kapkoros Small Hydropower Project Kenya. https://sanimaengineering.com/project/kipsonoi-kapkoros-small-hydropower-project-kenya/.
176. Offshore Wind. Kenya OKs WERPO's 100 MW Wave Energy Project. https://www.offshorewind.biz/2014/10/06/kenya-oks-werpos-100-mw-wave-energy-project/ (2014).
177. IPPJournal. 3.6 MW Kipsonoi hydropower project in Kenya. *Energy Finance & Investment* https://ippjournal.com/project/3-6-mw-kipsonoi-hydropower-project-in-kenya (2024).
178. Hydro Power International. Kaiha 2 small hydro-power plant. (2021).
179. pv magazine. Liberia plans 15 MW/10 MWh solar-plus-storage project – pv magazine International. https://www.pv-magazine.com/2023/08/22/liberia-plans-15-mw-10-mwh-solar-plus-storage-project/ (2023).
180. Mining Review Africa. Hydro-electric power plant for Hummingbird Dugbe project. https://www.miningreview.com/west-africa/positive-assessment-of-hydro-electric-power-plant-for-hummingbird-dugbe-project/ (2015).
181. Construction Review Online. Liberia signs US $34m deal for Gbedin waterfalls hydropower project. *Hydropower* https://constructionreviewonline.com/news/liberia-signs-us-34m-deal-for-gbedin-waterfalls-hydropower-project/ (2020).
182. afrik21. Libya government launches construction of a solar power plant in kufra. https://www.afrik21.africa/en/libya-government-launches-construction-of-a-solar-power-plant-in-kufra/ (2022).
183. LibyaHerald. Total Energies, GECOL and REAoL launch 500 MW Sadada solar power plant project. *Business* https://libyaherald.com/2022/06/total-energies-gecol-and-reaol-launch-500-mw-sadada-solar-power-plant-project/ (2022).
184. Electricity Alert. Electricity Alert: Madagascar. *Electricity in Africa and the future* https://electricityalert.blogspot.com/p/madagascar.html?m=1.
185. Scatec. The Government of Malawi, IFC, Scatec JV and EDF sign a binding agreement to develop the Mpatamanga hydropower plant - Scatec. https://scatec.com/2022/09/06/the-government-

of-malawi-ifc-scatec-jv-and-edf-sign-a-binding-agreement-to-develop-the-mpatamanga-hydropower-plant/ (2022).
186. Mining in Malawi. Mining in Malawi ». https://mininginmalawi.com/mining-in-malawi-462/ (2018).
187. afrik21. Senegal-Mali CMEC to build gourbassi hydroelectric dam. https://www.afrik21.africa/en/senegal-mali-cmec-to-build-gourbassi-hydroelectric-dam/ (2020).
188. African Energy. Mali: Turkish consortium to build Taoussa dam and hydro plant. https://www.africa-energy.com/news-centre/article/mali-turkish-consortium-build-taoussa-dam-and-hydro-plant (2020).
189. Ministére de l'Environnement et du Développement Durable. *Contribution Determinée Nationale Actualisée CDN 2021 - 2030*. (2021).
190. VINCI Construction. Abdelmoumen pumped storage hydroelectric plant. https://www.vinci-construction-projets.com/en/realisations/abdelmoumen-pumped-storage-.
191. MEED. Morocco plans oil shale power project. *Meed Editorial* https://www.meed.com/morocco-plans-oil-shale-power-project/ (2009).
192. Nareva. Dakhla Desalination Project. https://www.nareva.ma/en/project/dakhla-desalination-project. (2024).
193. NS ENERGY. Mphanda Nkuwa Hydropower Project - NS Energy. *Projects* https://www.nsenergybusiness.com/projects/mphanda-nkuwa-hydropower-project/ (2019).
194. AIM. Chapo pledges construction of Moamba-Major Dam. *Agência de Informação de Moçambique* https://aimnews.org/2024/09/16/chapo-pledges-construction-of-moamba-major-dam/ (2024).
195. ESI Africa. Two wind farms totalling 90MW are set to be built in Namibia. https://www.esi-africa.com/renewable-energy/two-wind-farms-totalling-90mw-are-set-to-be-built-in-namibia/ (2019).
196. NS ENERGY. Paladin Energy to re-start production at Langer Heinrich Mine in Namibia. https://www.nsenergybusiness.com/news/paladin-energy-to-re-start-production-at-lhm/ (2022).
197. African Energy. Niger: Zinder HFO commissioning, Malbaza project cranks up | African Energy. *Live Data* https://www.africa-energy.com/live-data/article/niger-zinder-hfo-commissioning-malbaza-project-cranks (2022).
198. Reuters. Nigeria seeks to restart four state oil refineries by end 2024 | Reuters. https://www.reuters.com/markets/commodities/nigeria-seeks-restart-four-state-oil-refineries-by-end-2024-minister-2023-08-25/ (2023).
199. Vanguard. Brazil build N144b power plant in Bayelsa - Vanguard News. https://www.vanguardngr.com/2014/08/fg-brazil-build-n144b-power-plant-bayelsa/ (2014).
200. Power Online. Groot Property Group (Pty) Ltd And Sparkle Energy Ltd To Build The World's Largest 4000 MW Gas-Fired Power Plant In Nigeria. *News* https://www.poweronline.com/doc/groot-property-group-pty-ltd-and-sparkle-0001 (2011).
201. financial Nigeria. Norwegian solar company secures 100mw project in Nigeria. https://www.financialnigeria.com/login.html (2021).
202. Premium Times. Shiroro to build 300mw solar power plant. https://www.premiumtimesng.com/ (2015).
203. Leadership. Farin Ruwa Power Plant To Inject 40 Megawatts To National Grid – Gov Sule. https://leadership.ng/farin-ruwa-power-plant-to-inject-40-megawatts-to-national-grid-gov-sule/#google_vignette (2023).
204. RwandaYP. Rwaza Hydro Power - Ruhengeri, Rwanda - Contact Number, Email Address. https://www.rwandayp.com/company/3965/Rwaza_Hydro_Power (2016).
205. African Energy. Orascom plans methane plant. https://www.africa-energy.com/news-centre/article/orascom-plans-methane-plant (2011).
206. Gesto Energy. Rwanda Investors Plan 10 Megawatts Solar Plant in Kayonza. https://gestoenergy.com/rwanda-investors-plan-10-megawatts-solar-plant-in-kayonza/.
207. Prime Energy. Rukarara VI. https://primeenergyltd.com/cases/rukarara-vi/.

208. NGALI ENERGY. Base II Small Hydropower Plant - Ngali Energy. https://ngalienergy.com/spip.php?article36.
209. EnDev Rwanda. *Status of the Hydropower Sector in Rwanda*. (2016).
210. African Energy. São Tomé and Príncipe: Bids requested for $10m hydro plant rehabilitation. https://www.africa-energy.com/live-data/article/sao-tome-and-principe-bids-requested-10m-hydro-plant (2022).
211. Seychelles News Agency. Seychelles to host world's largest salt-water floating solar plant - Seychelles News Agency. http://www.seychellesnewsagency.com/articles/18468/Seychelles+to+host+world%27s+largest+salt- (2023).
212. African Energy. Sierra Leone: Marampa mine to commission 22MW plant. https://www.africa-energy.com/news-centre/article/sierra-leone-marampa-mine-commission-22mw-plant (2022).
213. NS ENERGY. Sierra Rutile signs MOU with Smol Pawa for Moyamba hydro power project - NS Energy. *News* https://www.nsenergybusiness.com/news/newssierra-rutile-signs-mou-with-smol-pawa-for-moyamba-hydro-power-project-101213-4143135/ (2013).
214. InfraCo Africa. Bumbuna Hydro II. https://infracoafrica.com/project/bumbuna-hydro-ii/ (2024).
215. pv magazine. Construction begins on South Africa's first city-run solar plant – pv magazine International. https://www.pv-magazine.com/2024/10/09/construction-begins-on-south-africas-first-city-run-solar-plant/ (2024).
216. AfriCoast Investments. Rooikat and Meerkat Hydropower | AfriCoast Investments. https://africoastinvestments.co.za/project/rooikat-and-meerkat-hydropower/.
217. Jonker Klunne, W. Small hydropower in Southern Africa - an overview of five countries in the region. *Journal of Energy in Southern Africa* **21**, (2013).
218. Sawmilling in South Africa. York Timbers' proposed R1.4bn biomass plant to feed national grid - Sawmilling in South Africa. https://www.timber.co.za/news/article/york-timbers-proposed-r14bn-biomass-plant-to-feed-national-grid.
219. George Herald. 300 million power station for George. https://www.georgeherald.com/News/Article/Local-News/tweegesig-op-facebook-kloon-en-kul-201901080837 (2019).
220. eyeRadio. Rumbek power station reopens after a decade in decay - Eye Radio. https://www.eyeradio.org/rumbek-power-station-reopens-after-a-decade-in-decay/#google_vignette (2023).
221. Remis, T. *ENERGY SECTOR STATUS AND POTENTIAL RESOURCES IN SOUTH SUDAN*. https://mofp.gov.ss/?dlp_document=energy-sector-status-and-potential-resources-in-south-sudan. (2023).
222. OBOR. Belt and Road Energy Cooperation. http://obor.nea.gov.cn/tenderDetails.html?id=57&webSiteId=2 (2023).
223. Xinguanet. Chinese expert urges South Sudan to invest in hydropower to spur development - Xinhua | English.news.cn. http://www.xinhuanet.com/english/2019-12/04/c_138605860.htm (2019).
224. Multiconsult-Group. Fula Rapids Hydropower Project. https://www.multiconsultgroup.com/projects/fula-rapids-hydropower-project/.
225. ABiQ. Sudan calls for bidders to build own and operate a thermal power plant. https://www.abiq.io/sudan-calls-for-bidders-to-build-own-and-operate-a-thermal-power-plant.
226. Mekki Center for African Affairs. Despite critical transition, Sudan launches first solar power plant. https://mekkicenter.com/malgre-la-transition-critique-soudan-a-lance-la-premiere-centrale-solaire-despite-critical-transition-sudan-launches-first-solar-power-plant/ (2020).
227. ThinkGeoenergy. Tanzania plans up to 200 MW geothermal development by 2025. https://www.thinkgeoenergy.com/tanzanias-tgdc-plans-up-to-200-mw-geothermal-development-by-2025/ (2021).

228. African Energy. Tanzania: Nachu graphite production expected from early 2025 as Tesla signs offtake deal. https://www.africa-energy.com/news-centre/article/tanzania-nachu-graphite-production-expected-early-2025-tesla (2023).
229. Bagamoyo Sugar. Members of the press visit bagamoyo sugar factory in Makurunge, Bagamoyo | Bagamoyo. https://bagamoyosugar.com/members-of-the-press-visit-bagamoyo-sugar-factory-in-makurunge-bagamoyo/ (2022).
230. AMEA Power. AMEA Power Reaches Financial Close on the 120MW Solar Power Plant in Tunisia – AMEA Power. https://www.ameapower.com/amea-power-reaches-financial-close-on-the-120mw-solar-power-plant-in-tunisia/ (2023).
231. Boona. Pango Uganda Limited to build a 13.6MW mini-hydropower dam in Rukungiri. - 91.8 Boona FM. https://boonafm.com/pango-uganda-limited-to-build-a-13-6mw-mini-hydropower-dam-in-rukungiri/ (2023).
232. Creative Solutions for Infrastructure. Cresta Buhindagi. https://cvlsolutions.ca/projects.
233. open corporates. Elgon Sisi Hydro power Limited. https://opencorporates.com/companies/ug/80010004258046.
234. ThinkGeoenergy. Pushing geothermal power and direct use project in Zambia, Kalahari GeoEnergy | ThinkGeoEnergy - Geothermal News & Insights. https://www.thinkgeoenergy.com/pushing-geothermal-power-and-direct-use-project-in-zambia-kalahari-geoenergy/ (2020).
235. Factor This. African countries sign agreement to develop hydropower on Luapula River. https://www.renewableenergyworld.com/energy-business/new-project-development/african-countries-sign-agreement-to-develop-hydropower-on-luapula-river/#gref (2022).
236. Frontier Energy. Great Zimbabwe Hydro Project. https://frontier.dk/the-great-zimbabwe-hydro-project/.
237. Factor This. Zimbabwe utility awards EPC contract for 30-MW Gairezi hydropower plant. https://www.renewableenergyworld.com/energy-business/energy-finance/zimbabwe-utility-awards-epc-contract-for-30-mw-gairezi-hydropower-plant/#gref (2016).
238. NRE. Hauna Hydroelectric Power Station - Nyangani Renewable Energy. https://www.nrezim.com/projects/hauna-hydroelectric-power-station/.
239. Kottek, M., Grieser, J., Beck, C., Rudolf, B. & Rubel, F. World Map of the Köppen-Geiger climate classification updated. *Meteorologische Zeitschrift* **15**, 259–263 (2006).
240. Dziegielewski, B. & Bik, T. *Water Use Benchmarks for Thermoelectric Power Generation Project Completion Water Use Benchmarks for Thermoelectric Power Generation*. 213 (2006).
241. SENES Consultants Limited. *Methods to Assess the Impacts on the Natural Environment of Generation Options*. (2005).
242. Hoffmann, J., Forbes, S. & Feeley, T. Estimating freshwater needs to meet 2025 electricity generating capacity forecasts. *The United Nations World Water Development Report* 12 pp. (2004).
243. Meldrum, J., Nettles-Anderson, S., Heath, G. & Macknick, J. Life cycle water use for electricity generation: a review and harmonization of literature estimates. *Environmental Research Letters* **8**, 015031 (2013).
244. Berry, J. E., Holland, M. R., Watkiss, P. R., Boyd, R. & Stephenson, W. *Power Generation and the Environment - a UK Perspective. ExternE: Externalities of Energy*. (1998).
245. NETL. *Estimating Freshwater Needs to Meet Future Thermoelectric Generation Requirements (2010 Update)*. (2010).
246. CEC. *Environmental Performance Report of California's Electrical Generation System: Final Staff Report*. (2008).
247. Pate, R., Hightower, M., Cameron, C. & Einfeld, W. *Overview of Energy-Water Interdependencies and the Emerging Energy Demands on Water Resources*. 1–19 (2007).
248. Aspen Environmental Group. *Final Environmental Impact Report: Topaz Solar Farm Conditional Use Permit*. (2011).
249. Pasqualetti, M. & Kelley, S. *The Water Cost of Electricity in Arizona: A White Paper*. *Arizona Water Institute* 1–2 (2007).

250. Gleick, P. H. Water and Energy. *Annual Review Energy Environment* **19**, 267–99 (1994).
251. Sahm, A., Gray, A., Boehm, R. & Stone, K. Cleanliness Maintenance fro an Amonix Lens System. in *International Solar Energy Conference ISEC2005* (Orlando, FL, 2005).
252. Vaca-Jiménez, S., Gerbens-Leenes, P. W. & Nonhebel, S. The water footprint of electricity in Ecuador: Technology and fuel variation indicate pathways towards water-efficient electricity mixes. *Water Resources and Industry* **22**, 100112 (2019).
253. NETL. *Life Cycle Analysis: Integrated Gasification Combined Cycle (IGCC) Power Plant*. (2010).
254. Burkhardt, J. J., Heath, G. A. & Turchi, C. S. Life cycle assessment of a parabolic trough concentrating solar power plant and the impacts of key design alternatives. *Environmental Science and Technology* **45**, 2457–2464 (2011).
255. Turchi, C. S., Wagner, M. J. & Kustcher, C. F. *Water Use in Parabolic Trough Power Plants: Summary Results from WorleyParson's Analyses*. 102 (2010).
256. DOE. *Concentrating Solar Power Commercial Application Study: Reducing Water Consumption of Concentrating Solar Power Electricity Generation. Report to Congress*. (2008).
257. Viebahn, P., Kronshage, S. & Trieb, F. *Final Report on Technical Data, Costs, and Life Cycle Inventories of Solar Thermal Power Plants.* (2008).
258. Nicholson, S. & Garvin, H. Life Cycle Emissions Factors for Electricity Generation Technologies. *NREL Data Catalog* (2025) doi:10.7799/1819907.
259. EPRI. *Literature Review and Sensitivity Analysis of Biopower Life-Cycle Assessments and Greenhouse Gas Emission*. https://www.epri.com/research/products/1026852 (2013).
260. Whitaker, M., Heath, G. A., O'Donoughue, P. & Vorum, M. Life Cycle Greenhouse Gas Emissions of Coal-Fired Electricity Generation. *Journal of Industrial Ecology* **16**, (2012).
261. Eberle, A., Heath, G. A., Carpenter Petri, A. C. & Nicholson, S. R. *Systematic Review of Life Cycle Greenhouse Gas Emissions from Geothermal Electricity*. (2017) doi:10.2172/1398245.
262. O'Donoughue, P. R., Heath, G. A., Dolan, S. L. & Vorum, M. Life Cycle Greenhouse Gas Emissions of Electricity Generated from Conventionally Produced Natural Gas. *Journal of Industrial Ecology* **18**, 125–144 (2014).
263. Sathaye, J. *et al.* Renewable Energy in the Context of Sustainable Development. in *IPCC Special Report on Renewable Energy Sources and Climate Change Mitigation* (IPCC, 2011).
264. Warner, E. S. & Heath, G. A. Life Cycle Greenhouse Gas Emissions of Nuclear Electricity Generation. *Journal of Industrial Ecology* **16**, (2012).
265. Hsu, D. D. *et al.* Life Cycle Greenhouse Gas Emissions of Crystalline Silicon Photovoltaic Electricity Generation. *Journal of Industrial Ecology* **16**, (2012).
266. Burkhardt, J. J., Heath, G. & Cohen, E. Life Cycle Greenhouse Gas Emissions of Trough and Tower Concentrating Solar Power Electricity Generation. *Journal of Industrial Ecology* **16**, (2012).
267. DOE. *Wind Vision: A New Era for Wind Power in the United States*. (2015) doi:10.2172/1220428.
268. DOE. *Hydropower Vision: A New Chapter for America's 1st Renewable Electricity Source (Full Report)*. (2016) doi:10.2172/1330494.
