# Supplementary material for: Forecast on Africa’s power production up to 2030 with related water use and CO2 emissions
Source: Nat Commun. 2026 May 7;17:6154. doi: 10.1038/s41467-026-72692-2 (PMC13365816; doi:10.1038/s41467-026-72692-2)
Supplement: Supplementary file 2 — Descriptions of Additional Supplementary Files [file 41467_2026_72692_MOESM2_ESM.pdf]

Description of Additional Supplementary Files

Supplementary Data 1: INVENTORY, the inventory of power plants.

Supplementary Data 2: Past, current and forecasted electricity generation by source (GWh) on the national level.

Supplementary Data 3: REGR_MVAR national data.

Supplementary Data 4: Annual and monthly evaporation amounts (in mm) per hydropower plant.

Supplementary Data 5: National values 1990- 2030 Energy (GWh), water withdrawal (m3), water consumption (m3) and CO2 emissions (tCO2e).

Supplementary Data 6: Power plants for the NDC scenario, based on INVENTORY.

Supplementary Data 7: National amounts (per fuel type) for electricity production per installed capacity.
