# Supplementary material for: Forecast on Africa’s power production up to 2030 with related water use and CO2 emissions
Source: Nat Commun. 2026 May 7;17:6154. doi: 10.1038/s41467-026-72692-2 (PMC13365816; doi:10.1038/s41467-026-72692-2)
Supplement: Supplementary file 4 — Transparent Peer Review file [file 41467_2026_72692_MOESM4_ESM.pdf]

nature portfolio

Peer Review File

Forecast on Africa’s power production up to 2030 with related water use and CO2 emissions

Corresponding Author: Dr Davy Vanham

This file contains all reviewer reports in order by version, followed by all author rebuttals in order by version.

Version 0:

Reviewer comments:

Reviewer #2

(Remarks to the Author)
General Assessment

This manuscript provides a detailed spatial inventory of power plants in Africa from 2020 to 2030. The topic is highly relevant, given Africa's ongoing challenges with severe power shortages and the additional pressure to align with global Sustainable Development Goals (SDGs) and carbon neutrality targets. Understanding Africa's power plants' development trajectory is critical for formulating sustainable development plans. However, this manuscript falls short in terms of its methodological rigor and the reliability of its conclusions. The lack of methodological transparency, combined with insufficient data support and limited discussions, undermines the overall value of this work. My comments：

Key Results

The manuscript's main contribution lies in constructing a unique dataset on African power plants. This dataset has the potential to significantly advance research on Africa's energy development and efforts to alleviate energy poverty. However, the predictive results, including carbon emissions, are less convincing due to the simplistic modeling approach and insufficient consideration of critical factors affecting energy systems in Africa.

Originality and Significance

The focus on Africa's power plants is undoubtedly significant, particularly in achieving SDGs, alleviating energy poverty, and addressing carbon neutrality goals. However, the manuscript's potential impact is diminished by the questionable reliability of the predictions and the lack of actionable insights derived from the results.

Data and Methodology

1. Data Sources:

The sources of the data used in the manuscript are unclear. Transparent and reliable data are essential for reproducibility and credibility. Without clear documentation, it is difficult to assess the validity of the dataset or replicate the study.

Suggestion: Clearly specify the origin of the data, how it was collected, and any preprocessing steps undertaken. Their credibility should be justified if proprietary or new data sources were utilized.

2. Methodological Transparency:

The description of the modeling approach, particularly the polynomial regression used for electricity production forecasting, lacks sufficient detail.

l What specific factors were included in the regression models?

l Were exogenous variables such as GDP, population growth, or international development aid considered?

l Were future scenarios, such as policy changes or technological advancements, incorporated into the analysis?

Without this information, it is impossible to assess the chosen method's appropriateness or the predictions' robustness.

Suggestion: Provide a step-by-step explanation of the modeling process, including the rationale for selecting specific methods and the assumptions made. Include any validation metrics used to evaluate the accuracy of the predictions.

3. Predictive Reliability:

Using historical growth rates from 1990 to 2020 as the basis for future energy production forecasts is overly simplistic. Many factors influence electricity production, including demand dynamics, policy interventions, and international financing. The lack of consideration for these factors raises doubts about the reliability of the results.

Suggestion: Incorporate a more comprehensive modeling framework, such as integrated assessment models (IAMs) or system dynamics models, to capture the multifaceted drivers of energy production.

Conclusions

The conclusions drawn in the manuscript lack robustness and reliability due to significant methodological shortcomings:

1. Overreliance on Historical Data in Forecasting

The article uses linear and polynomial regression to predict electricity production, relying solely on historical growth rates from 1990 to 2020 without incorporating exogenous variables or scenario assumptions. This approach overlooks the dynamic drivers of electricity demand (e.g., economic growth, population changes, and industrial restructuring) as well as the influence of policies and technologies on production. Additionally, external shocks such as climate change and geopolitical risks, which could significantly impact Africa's power systems, are not considered, further undermining the credibility of the forecasts.

Suggestion: Include economic variables (e.g., GDP and population growth rates), policy factors (e.g., clean energy policies, power market reforms), and external shocks in the model. Combine econometric models with scenario-based analysis to produce more robust forecasts under different conditions.

2. Lack of Feasibility in Energy Structure Predictions

The article predicts energy structures based on countries' stated targets without clarifying the source of these targets or evaluating their feasibility. For instance, the assumed significant increase in renewable energy shares in economically weaker African countries lacks consideration of the infrastructure and resources required for such transitions.

Suggestion: Clarify the data sources and origin of the targets. Use constraints (e.g., economic capacity, water resource limitations) and conduct cost-benefit analyses or technical feasibility studies to ensure the projections align with reality.

3. Economic and Technical Feasibility

Predictions of extreme increases in electricity production for certain countries (e.g., South Sudan, DRC, Namibia) seem detached from their current economic and technical capacities. Such estimates should be critically evaluated for feasibility.

Suggestion: Compare the predicted growth rates with historical trends and consider incorporating metrics of economic and infrastructural readiness.

4. Absence of Sensitivity Analysis for Forecasts

The article lacks sensitivity analysis of the model's key assumptions, such as the constancy of historical growth rates, and does not evaluate how results vary under different scenarios. This static assumption approach fails to capture the dynamic nature of real-world situations and does not reveal the model's scope and limitations.

Suggestion: Introduce sensitivity analysis to test the impact of key variables on predictions. Utilize Monte Carlo simulations or scenario analysis to generate various outcomes under different scenarios. Clearly outline the model's limitations and explore potential sources of errors.

5. Ignoring Water Resource Constraints in Hydropower Expansion Predictions

The article forecasts increased hydropower shares without considering water resource availability or the seasonal variability of water supply. For instance, severe water scarcity in some African regions may limit hydropower expansion potential, but this issue is not addressed in the study.

Suggestions: Incorporate GIS data to assess water resource availability and its constraints on hydropower development. Discuss the potential ecological and social impacts of hydropower expansion, such as migration and environmental degradation.

6. Lack of Justification for Overoptimistic Growth Projections

The article predicts extraordinarily high growth rates in electricity production for specific countries (e.g., South Sudan +7443%, Democratic Republic of Congo +2941%). However, it does not explain the feasibility of these results. Given these regions' poor infrastructure and economic conditions, such massive growth appears unrealistic.

Suggestion: To validate their feasibility, compare the projections with current infrastructure, economic capacity, and technological conditions. Offer phased prediction pathways to clarify the consistency of short- and long-term targets. Analyze the investment and technological requirements for such growth to assess their practicality.

References

The quality and timeliness of the references are insufficient. Many cited works are outdated, which undermines the manuscript's ability to position itself within the current state of research.

Suggestion: Update the references to include recent and high-quality studies. This will also help identify potential methodological improvements and provide context for the manuscript's contributions.

Clarity and Context

1. Main Section:

This section lacks a clear and logical structure, making it difficult for readers to identify key findings and their significance.

Suggestion: Reorganize the main text to ensure clear delineation between methods, results, and discussions.

2. Discussion Section:

The discussion is underdeveloped. It fails to adequately address the implications of the findings, their alignment (or misalignment) with previous studies, and their potential applications. For instance, the manuscript does not discuss how its results could inform Africa's energy policies or international climate frameworks.

Suggestion: Expand the discussion to include comparisons with existing literature, practical implications, and potential policy recommendations. Address critical questions, such as how African countries can balance development goals with sustainability.

3. Visualizations:

The figures in the manuscript are poorly labeled and often confusing. Specific issues include:

l Figure 1: Incomplete legend explanations; ensure all abbreviations are defined.

l Figure 2: Misleading title; revise "Energy production" to "Electricity production."

l Figure 3: Confusing legend with shapes and colors; simplify by using colors alone to represent energy types.

l Figure 8: Inconsistent labeling of bar charts; ensure all bars are clearly annotated.

Suggestion: Improve figure clarity by adhering to best practices for scientific visualization. Include a table summarizing scenario differences to improve readability.

Final Recommendation

The manuscript tackles an important topic with potential for significant impact. However, the current version is undermined by a lack of methodological rigor, insufficient discussion, and unclear presentation. Substantial revisions are required to bring the manuscript up to the standards of Nature Communications.

Reviewer #3

(Remarks to the Author)
The paper titled "Forecast on Africa’s power production up to 2030 with related water use and CO_2 emissions" provides a comprehensive and detailed analysis of the future landscape of electricity production in Africa. It effectively highlights the expected increases in electricity generation, water use, and CO_2 emissions from 2020 to 2030, using a robust methodological framework. The integration of data from various sources, including forecasts based on historical trends, current constructions, and national commitments (NDCs), adds depth and credibility to the study.

Merits:
• Comprehensive Database: The creation of a detailed spatial inventory of 3,139 individual power plants is a significant contribution, providing valuable insights into the future of Africa's electricity production.
• Robust Methodology: The use of multiple forecasting methods (INVENTORY, REGR_LIN, REGR_POL) and the consideration of NDC commitments ensure a thorough and well-rounded analysis.
• Environmental Impact Analysis: The focus on water use and CO_2 emissions highlights the environmental trade-offs associated with different energy sources, making the study relevant to policymakers and stakeholders.
• Regional Insights: The detailed breakdown of electricity production and environmental impacts by country and region offers valuable context and highlights disparities that need to be addressed.

Comments for Improvement:
1. Policy Recommendations: Expanding on specific policy recommendations for achieving electricity production goals while managing environmental impacts would add practical value to the study. Highlighting strategies for integrating renewable energy sources and improving energy efficiency could provide actionable insights for policymakers.
2. Technical Details: Providing more technical details about the power plants, such as the types of technologies used and their efficiencies, could offer deeper insights into the environmental trade-offs and technological advancements.
3. Stakeholder Engagement: Emphasizing the role of stakeholder engagement in achieving forecasted energy production goals and addressing environmental challenges can provide a more holistic view of the pathways forward.
4. Discussion on Uncertainties: Explicitly discussing potential uncertainties and limitations of the forecasts, such as the impact of policy changes, geopolitical factors, and climate variability, would strengthen the credibility and transparency of the study.

Additionally, there are some minor corrections suggested in the manuscript using “Track Changes” that would improve the readability of the paper.

Version 1:

Reviewer comments:

Reviewer #2

(Remarks to the Author)
The author has revised the manuscript in response to the reviewers' comments, supplemented additional content, and refined specific logical structures. This paper predicts electricity production, water consumption, and carbon emissions in Africa up to 2030 based on detailed power plant data. It demonstrates a solid foundation in data collection and preliminary scenario analysis. However, several notable shortcomings remain, including the need to enhance the scientific rigor and precision of the predictions, insufficient articulation of the research significance and practical value, and point-to-point responses that lack specificity and clarity. Particularly, despite extensive efforts in collecting power plant data, the predictive outcomes for the future appear much less than satisfactory. Specific opinions are outlined below.

1. The abstract requires further refinement. As the core summary of the entire paper, it currently exhibits the following main issues: First, its structure is somewhat loose, and key content lacks a clear logical hierarchy; second, it does not adequately elucidate the relationships among the research background, methodology, primary findings, and policy implications. It is recommended that the author rewrite the abstract by organizing the content in the sequence of "research background - research design - main findings - policy implications," emphasizing the study's innovation points and the practical significance of its conclusions. It will enable readers to rapidly grasp the essential elements of the paper.

2. This paper highlights that developing hydropower in Africa before 2030 will exacerbate regional water resource pressure. However, in predicting the future scale of hydropower, the model does not incorporate water resource sustainability as a prerequisite constraint, relying primarily on historical data and socio-economic variables for trend extrapolation. This approach risks reversing cause and effect logically. Water resources should serve as a fundamental constraint for hydropower planning rather than merely being an outcome of post-assessment. It is recommended that the author further clarify whether and how the availability of water resources is integrated into the hydropower development prediction model. If it is not incorporated, the author is advised to supplement the discussion regarding the impact of this assumption on the conclusions or explore the use of coupled modeling and other methods to more scientifically reflect the interrelationship

between water and electricity, thereby enhancing the rationality of the conclusions and the feasibility of policy recommendations.

3. This paper employs statistical methods such as linear extrapolation, polynomial regression, and multiple regression to predict electricity production, water consumption, and carbon emissions in Africa before 2030. While these methods are operationally straightforward and offer strong explanatory power, they exhibit several potential limitations when applied to long-term trend prediction. Previous reviewers have raised similar concerns, and although the authors have addressed these comments, my reservations regarding the results of this paper remain unaddressed. These methodological deficiencies constitute one of the primary factors affecting the overall quality of the paper.

(1) Model assumptions and extrapolation risks. Although the prediction horizon extends only to 2030, a relatively short timeframe, the likelihood of fundamental structural changes in technology or policy remains low. Nevertheless, linear and polynomial extrapolation methods inherently assume that historical trends will persist shortly, potentially obscuring nonlinear fluctuations caused by regional conflicts, extreme climatic events, or delays in commissioning major projects. It is recommended that the authors explicitly articulate the underlying model assumptions and critically evaluate their applicability and limitations for short-term predictions.

(2) Overfitting and extrapolation risks. Polynomial regression may overfit historical data, leading to considerable uncertainty in out-of-sample predictions. The authors are encouraged to provide a detailed justification for selecting polynomial order and the rationality of the extrapolation interval.

(3) Variable selection and omitted variable problem. The multiple regression analysis relies solely on population and GDP as independent variables, limiting its ability to capture the multifaceted driving mechanisms of electricity development comprehensively. In reality, electricity production is influenced by a wide range of factors, including but not limited to energy prices, investment and financing in power infrastructure, policy incentives or subsidies for renewable energy, trade dynamics in electricity and energy imports/exports, electricity demand structure, urbanization processes, technology penetration rates, and the impacts of climate change and frequent extreme weather events. It is recommended that the authors further justify the rationale behind their variable selection, assess the potential impact of omitted variables on the prediction outcomes, and attempt to incorporate additional key driving factors within the scope of available data to enhance the model's explanatory power and the robustness of its conclusions.

(4) Data dependence and scenario assumptions. The aforementioned methods are highly reliant on the quality of input data and historical trends, exhibiting limited sensitivity to uncertain factors such as future extreme events or policy shifts. To improve the robustness of the results, it is suggested that the authors integrate scenario analysis or sensitivity testing into their methodology. In conclusion, the authors are encouraged to provide a more thorough discussion of the model's applicable premises and potential limitations in the methodology section, supplemented by sensitivity analyses under different scenarios to enhance the scientific rigor of the research conclusions and their policy relevance. In its current form, the predictive framework lacks sufficient credibility.

4. This paper presents predictions for data beyond 2023. Has the actual data for 2024 been officially released? If the 2024 data is publicly available, it would be necessary to compare the model's predicted results with the latest empirical data to validate its accuracy and applicability. Such comparative analysis not only aids in evaluating the model's predictive capability but also strengthens the credibility of the research conclusions and their policy relevance. It is recommended that the author incorporate relevant analysis or discussion in the revised manuscript.

5. The discussion on the significance of the research in this paper remains somewhat inadequate. Two key issues warrant further consideration and elaboration: (1) The practical necessity of short-term prediction. Africa has consistently faced structural challenges, including insufficient investment, prolonged project implementation cycles, low electricity access rates, and significant regional disparities in power infrastructure development. Given that this paper focuses its predictions solely on 2030—only five years away—it is essential to clarify the rationale and urgency of such short-term forecasting. (2) Practical policy implications. While this paper provides a spatial inventory of African power plants and conducts relevant predictions, it is crucial to address the question of "so what." Does the research content offer tangible value? It is recommended that the author delve deeper into the specific policy implications of the prediction results. For instance, which countries require accelerated planning efforts? How can the future power structure be optimized under the current constraints of water resources and carbon emissions? Can the findings serve as risk warnings or decision-support tools for governments, regional organizations, and international institutions?

Reviewer #3

(Remarks to the Author)

Reviewer #4

(Remarks to the Author)
This study provides spatially detailed inventory of African power plants covering the period from 2020 to 2030. The study fills this gap by creating a database of 3,139 individual plants, including their location, commissioning year, electricity

production, and fuel type. The detailed spatial inventory and forecasts provided by this study can inform policymakers and investors about the potential environmental impacts and trade-offs associated with different electricity production strategies in Africa. The study compare the actual power plant construction plans (INVENTORY) with the commitments made in Nationally Determined Contributions (NDCs) highlighting potential discrepancies between plans and commitments.

My Comments

General Comments

1.Method used in the paper is absent in the abstract.
2.There are discrepancies between the numbers in the Excel file and those reported. For instance, line 85 mentions 3,246 individual power plants, but the Excel file indicates a different figure. Same as line 152. Please verify these and other reported numbers.
3.Line 248; Kenya, not Kenia.
4.Kindly note that the statements found in lines 285 to 287 contradict the information presented in Figure 7. Please review and revise lines 277 to 289 to enhance clarity.
5.Line 307; please review the conceptual use of energy and electricity.
6.Line 381; The abbreviation WEF has just appeared. Kindly review and clarify its meaning.

Data reliability and completeness:

The research extensively utilizes publicly accessible and secondary data sources to gather information on power plants. However, this data may contain inconsistencies or omissions, particularly concerning smaller nations or recent projects. Such issues could result in inaccurate estimations of electricity generation and its associated effects.
Take Ghana as an example; the authors utilized data from the IEA and other secondary sources. Nonetheless, the power generation figures reported by Ghana's Energy Commission for 2020 and other years show a notable discrepancy when compared to the IEA's 2020 data for Ghana.

Recommendation
I suggest using primary data at the national or country level for power generation across all African nations, except when such data is unavailable, in which case the IEA and IRENA database can be used.

When gathering data on electricity generation by country, did you consider the electricity trade between African nations? This aspect could significantly impact the study's findings, particularly regarding CO2 emissions and water usage and consumption.

Recommendation
I recommend that actual electricity generation are accounted for by considering power trade among neighboring African countries.

The electricity generation per installed capacity data is not available in the paper.

Recommendation
Please include the electricity generation per installed capacity data in the Supplementary Material, as merely citing such key data used in the calculations is insufficient.

Methods
The methodology is generally robust and suitable for underpinning the study's assumptions and conclusions. By employing a variety of forecasting techniques, spatial analysis, and considering environmental effects, the study offers a thorough perspective on Africa's prospective energy scenario. Although the approach has been indirectly assessed by comparing it with historical patterns and policy commitments, it could benefit from further validation measures.

Method weakness

The water withdrawal and consumption calculations use average values for different power plant types. This may not capture the variability in water use due to specific local conditions, cooling technologies, or plant designs.

Recommendations
Please use specific values for water withdrawal and consumption for different power plants.

The study doesn't appear to account for potential improvements in power plant efficiency or new technologies that could emerge by 2030. This could affect both electricity production estimates and associated water use and CO2 emissions.

The INVENTORY forecast assumes that all planned and under-construction power plants will be completed by 2030. This may not account for potential delays, cancellations, or changes in energy policies. The other forecasting methods (REGR_LIN, REGR_POL, REGR_MVAR) are based on historical trends, which may not accurately reflect future

developments, especially in rapidly changing energy landscapes

Recommendation
Please provide a limitation section of the manuscript and detail all the limitation of the study for transparency.

Recommendation for method presentation

The method should include all equations related to the regression analysis. Additionally, please supply a list of the variables used in the regression analysis along with their definitions.

Recommendation for method improvement
The method doesn't delve into the economic feasibility or cost-effectiveness of different power generation options, which could significantly influence actual development paths. Please consider economic parameters as well.

Version 3:

Reviewer comments:

Reviewer #2

(Remarks to the Author)
This study presents a highly detailed spatial inventory of operational, under-construction, and planned African power plants between 2020 and 2030, based on an intensive data mining effort that identifies 3,139 individual facilities. We appreciate the authors' substantial efforts in revising the manuscript and their thoughtful responses to the reviewer comments. However, given the high standards of Nature Communications, there remain several key areas where the manuscript should be further improved to meet publication criteria. Below are my detailed comments:

1. Enhance the Introduction Section

While the introduction provides some background and motivation, it remains underdeveloped in several crucial aspects. The authors are encouraged to revise and expand the introduction along the following lines:

(1) The current manuscript lacks engagement with related academic studies. Have other scholars conducted similar work forecasting energy production, water use, or CO_2 emissions in Africa or other regions? What are the major findings and methodological approaches in those studies? How does this work differ from or improve upon them? This context is critical to help position the contribution of the present study and underscore its originality.

(2) The introduction would benefit from a paragraph summarizing the most important and surprising findings of the study. For example, differences between the INVENTORY and NDC projections on renewable energy sources could be highlighted here.

(3) Compared with existing studies, what contributions does this research make? The authors need to add a paragraph explaining, from multiple perspectives, how this study extends the existing literature and clarify why these extensions are important.

(4) To improve readability and guide the reader through the paper, the authors should add a paragraph at the end of the introduction that outlines the structure of the remaining sections.

2. Reorganize the Overall Structure of the Manuscript

The structure of the paper is not well-organized. Several major revisions are suggested:

(1) The manuscript lacks a dedicated literature review section. Moreover, there is minimal discussion of existing studies throughout the paper. This weakens the justification for the study and makes it difficult to assess its novelty. A stand-alone literature review or a dedicated subsection within the introduction would be highly beneficial.

(2) Currently, the methods section appears later in the manuscript (Section 5), while results are presented earlier (Section 2). This ordering is counterintuitive and should be reversed. A more standard and reader-friendly structure would follow the format: Introduction – Literature Review – Methods – Results – Discussion – Conclusion.

(3) There is no distinct Conclusion and Policy Implications section. Although the authors summarize the findings in Section 3

“Discussion,” discussion and conclusion are not the same. In the discussion section, the authors should connect the key findings of this study to existing literature or real-world contexts, explaining their significance and validity. In the conclusion and implications section, the authors should clearly present the main findings and provide targeted implications or recommendations based on these findings.

3. Deepen the Analysis of Key Results

The results section is currently structured around three forecasts: electricity production, water use, and CO_2 emissions. While this is logical, the treatment of these results is too descriptive and lacks analytical depth. As it stands, the manuscript reads more like a technical report than a scholarly article. For example:

“For the renewables, considerably more power is produced from hydropower in INVENTORY (517,501 GWh) compared to NDC (378,598 GWh). For solar it is the opposite (24,570 GWh and 179,645 GWh, respectively), as well as for wind (30,685 GWh and 109,627 GWh, respectively).”

This is a very interesting finding, but it is not sufficiently explained or contextualized. Why is hydropower overrepresented in INVENTORY compared to NDCs, while solar and wind are underrepresented? Are these patterns a result of historical investment decisions, differences in construction timelines, policy priorities, or financing constraints? The authors should unpack these patterns and discuss the implications for energy policy and climate planning in Africa.

4. As the authors mention in the study’s limitations section: “The INVENTORY forecast assumes that all planned and under-construction power plants will be completed by 2030. This assumption is made based upon the relatively short time window until 2030 and the average planning and construction time for power plants.” The assumption may bias the forecasts in favor of slower-to-build technologies like hydropower and thermal plants, while underestimating the role of fast-deploying renewables such as solar and wind. Construction times for utility-scale solar PV and onshore wind are typically less than 12 months, making them highly scalable over the short term. This could partially explain why solar and wind outputs in INVENTORY fall below those projected in NDCs, and why hydropower is overrepresented. Given Africa’s increasing commitment to low-carbon development, these fast-to-deploy technologies may witness significant growth in the near term—an aspect not fully captured in the INVENTORY-based forecast.

Reviewer #4

(Remarks to the Author)
The authors have modified the article significantly and responded very well to the comments. Hence may be accepted in my opinion if other reviewers/ editorial review agreed.

Version 4:

Reviewer comments:

Reviewer #2

(Remarks to the Author)
This study forecasts Africa’s electricity production, water use, and CO_2 emissions to 2030 using a detailed spatial inventory of power plants, but the revised manuscript remains unsatisfactory. Most of the concerns raised in the previous review have not been adequately addressed. Major deficiencies persist in the literature review, model specification, forecasting methods, the robustness of results, and the paper’s overall originality. The specific comments are as follows:
1. I previously noted that the manuscript lacks engagement with related academic studies. It remains unclear how this work positions itself relative to prior research on forecasting energy production, water use, or CO_2 emissions in Africa or other regions. Although the authors added some discussion of alternative databases, they still fail to review the major findings and methodological approaches of comparable studies or explain how their work differs or improves upon them. This omission leaves the contribution poorly contextualized and weakens the scholarly significance of the paper.
2. I asked the authors to clarify the study’s contribution compared with existing literature, emphasizing methodological, content-based, and theoretical innovations. Instead, the revision merely highlights differences from other databases and provides no substantive explanation of how this research extends existing knowledge or why any extensions would be important. Without a clear statement of novelty, the paper struggles to justify publication.
3. To improve readability, we recommended adding a paragraph at the end of the introduction outlining the structure of the remaining sections. The authors ignored this suggestion.
4. Despite I earlier requested for a thorough literature review, the authors only inserted a short discussion (Lines 137–153) about other databases, which is insufficient. The manuscript still lacks a substantive review of relevant empirical and theoretical studies, leaving readers without the necessary background to understand how this research fits into or advances the field.
5. I previously urged the authors to offer targeted policy recommendations based on their findings. They have not incorporated any such guidance. Without actionable policy implications, the study’s practical relevance is greatly

diminished, limiting its value to policymakers and stakeholders.
6. I raised concerns about a key modeling assumption: the INVENTORY forecast assumes all planned and under-construction plants will be completed by 2030. This biases the results toward slower-to-build technologies like hydropower and thermal plants and underestimates fast-deploying renewables such as solar and wind, which typically require less than 12 months for construction. While the authors acknowledge this issue in passing, they have not adjusted their model to account for it. This uncorrected assumption likely skews the forecast and weakens the credibility of the results.
7. The forecasting approach relies only on three basic regression models—a linear regression (REGR_LIN), a polynomial regression (REGR_POL), and a multivariate regression with population and GDP as predictors (REGR_MVAR). Such simple extrapolations are insufficient for a study of this scale and importance. The authors should benchmark these results against more advanced forecasting techniques (e.g., time-series models like ARIMA or machine-learning methods) to demonstrate the robustness and accuracy of their projections.
8. The projections also lack a sensitivity analysis to test how results respond to key assumptions, such as variations in plant construction timelines. Without examining how uncertainties in these factors affect the outcomes, the reliability of the forecasts is significantly reduced, and readers cannot assess the stability of the conclusions under alternative scenarios.

Version 5:

Reviewer comments:

Reviewer #2

(Remarks to the Author)
The revision is substantially improved and is responsive to most of our previous concerns. At this stage, I believe the manuscript would benefit from further refinement in several areas. Addressing the points below would further improve the overall quality, clarity, and scholarly positioning of the paper.

Introduction: The first two paragraphs mainly emphasize why Africa's power production is an important topic, after which the Introduction quickly moves to describing the present study. I recommend inserting a dedicated paragraph (or two) before introducing the study design to summarize the current state of research in this area and to clarify what remains insufficient or unresolved in the existing literature. This would help readers understand more clearly why the present study is necessary and what specific gap it fills.
Introduction: Please include a short paragraph that explicitly summarizes the main contributions relative to prior studies. A clear contribution statement will help readers quickly grasp the manuscript's novelty and its specific value to the field.
While the Discussion is rich and wide-ranging, it currently reads largely as an interpretation of the authors' own results. I encourage the authors to more systematically relate key findings to prior empirical evidence and established conclusions in the literature. This would improve the scholarly integration of the paper and sharpen the interpretation of the results.
The manuscript should provide a clearer description of how the sample of 2,534 individual power plants was assembled and filtered. In addition, the authors should briefly discuss their representativeness.
Because the projections extend only to 2030 (a relatively near-term horizon), the manuscript should more explicitly justify why 2030 is the central milestone. Moreover, the forecasting dataset appears to end in 2023. If 2024 (and possibly 2025) observed data are now available for at least a subset of countries/technologies, the authors could compare these realized values with the model's short-horizon predictions as a simple out-of-sample validation.
The description of the workflow is internally inconsistent (e.g., the text refers to four steps but later mentions a "fifth forecast" and "step 5"). Please revise the Methods narrative so that the number of steps and the number of forecast scenarios are consistent throughout.
The delay assumptions (e.g., 1-year vs 2-year delays by technology classes, plus specific treatment for large hydro/nuclear) should be supported by either citations or empirical project-level evidence. In addition, please consider decomposing the sensitivity analysis into two or three simpler scenarios (e.g., "large hydro only," "fossil only," "wind/solar only") to help readers identify which technologies drive the uncertainty.

(Remarks on code availability)

Open Access This Peer Review File is licensed under a Creative Commons Attribution 4.0 International License, which permits use, sharing, adaptation, distribution and reproduction in any medium or format, as long as you give appropriate credit to the original author(s) and the source, provide a link to the Creative Commons license, and indicate if changes were made.
In cases where reviewers are anonymous, credit should be given to 'Anonymous Referee' and the source.
The images or other third party material in this Peer Review File are included in the article's Creative Commons license, unless indicated otherwise in a credit line to the material. If material is not included in the article's Creative Commons license and your intended use is not permitted by statutory regulation or exceeds the permitted use, you will need to obtain permission directly from the copyright holder.
To view a copy of this license, visit https://creativecommons.org/licenses/by/4.0/

NCOMMS-24-72853

Forecast on Africa's power production up to 2030 with related water use and CO2 emissions

S.D. Vaca-Jiménez, P.W. Gerbens-Leenes, Bunyod Holmatov, Raphael Vanham, Davy Vanham

First of all the authors would like to thank the editor(s) and the two referees for their time and energy invested. We appreciate the thoughtful comments of the referees.

A. General response to reviewers

Reviewer 3 provides an overall very positive evaluation of our paper. He/she writes that the authors use a robust methodological framework and highlights 4 merits of our paper : 1) Comprehensive Database; 2) robust methodology 3) Environmental Impact Analysis (focus on water use and CO2 emissions) and 4) Regional Insights.

We thank reviewer 3 for this overall evaluation

Reviewer 2 writes that "the manuscript tackles an important topic with potential for significant impact. However, the current version is undermined by a lack of methodological rigor, insufficient discussion, and unclear presentation. Substantial revisions are required". He/she writes:

- "The sources of the data used in the manuscript are unclear"
- He/she raises questions about the modelling (specifically the regression analyses). He/she indicates and overreliance on Historical Data in Forecasting (based on historical growth rates from 1990 to 2020) and a lack of Feasibility in Energy Structure Predictions. He/she recommends to use more sophisticated modelling by including economic variables (e.g., GDP and population growth rates), policy factors (e.g., clean energy policies, power market reforms), and external shocks in the model. He/she recommends to combine econometric models with scenario-based analysis to produce more robust forecasts under different conditions.
- He/she also indicates that there is an absence of Sensitivity Analysis for Forecasts and that the discussion section should be more developed (also recommended by Reviewer 3), especially with policy recommendations

We acknowledge Reviewer 2's comments and recommendations.

- We agree that we should have provided more clarity on the source of our data. We also agree 100% with Reviewer 2' statement "Transparent and reliable data are essential for reproducibility and credibility. Without clear documentation, it is difficult to assess the validity of the dataset or replicate the study"

- We have now **added a text in** a prominent part of the paper, **the introduction section**, **how we assembled the data for** the power plant inventory (**INVENTORY**). This clarity was indeed missing in our original paper, which could have led to the impression that INVENTORY is the result of modelling, which it is not. **We also added very detailed listings of all data sources in the Supplementary information** (Supplementary Tables 1,2,3,4,5,6 – combined covering 279 references). A new general overview table is provided as Table 2 in section 4 "Methods" of the main paper, in its introductory paragraphs. As such, we provide full transparency on all data sources used in this paper.

- It is **essential to emphasize that the main novelty of our paper is not modelling, but INVENTORY, which covers 3,139 individual plants** and is the result of an **intensive, time consuming data mining effort of open access data sources.**

The text we add in the introduction is the following (Lines 57-78):

> *The power plant inventory (INVENTORY) is a main novel output of this paper. It is not the result of a model. It is the result of an intensive, time consuming data mining effort of open access data sources. We used only open access sources, to be able to provide our resulting database open access. Data sources include GEM wiki[4] and Wikipedia[5], because they provide recent information on power plants, especially on the large ones. Other data sources used were Power Technology[6], a website on global energy industry, which also provides information on installed capacity and year of commission. We also used Open Street maps, reports from national ministries or international organisations (e.g. the Worldbank), scientific papers, companies and also newspapers, which give information on the opening or closure of specific plants. We checked and adapted location coordinates of all power plants using Google Maps. Data sources for each power plant are included in Supplementary Tables 1 to 6.*
>
> *INVENTORY provides a realistic analysis on existing power plants and a best estimate on power plants under construction, because the planning and construction of power plants takes time[7] and the forecast window up to 2030 is relatively short. According to Sovacool et al[7], nuclear power plants and larger hydropower dams have the longest mean construction times, i.e. seven and ten years, respectively. Small run-of-river power plants take less time to construct. Fossil-fuelled thermal plants have mean construction times of almost 5 years. Solar projects and wind farms show the lowest average construction times of about two and one year, respectively. There is however quite some variation on these averages. While our forecast window up to 2030 is only about five years, we are confident we include almost all planned and under construction power plants, as we restrict ourselves to plants that are currently under construction or are being planned, all to start operations until latest 2030.*

Reviewer 2 is very correct that there are much more sophisticated modelling frameworks than the regression models we use in the original paper (linear and polynomial regression, i.e. REGR_LIN and REGR_POLY, respectively). There are multiple review papers that discuss different modelling approaches and frameworks, such as Nikas, Gambhir, et al (2021), Fodstad

et al (2022) or McGookin et al (2024). In our revised paper, we refer to these papers in the discussion section.

In line with the recommendations of reviewer 2, **we added a (multivariate) regression analysis that accounts for the socioeconomic parameters population and GDP (called REGR_MVAR)**. We add the overall results with associated text description to Figure 1. The resulting electricity production forecast for 2030 (1,268,358 GWh/year) is only slightly higher than the REGR_LIN result (1,266,600 GWh/year) but quite lower than the REGR_POL result (1,490,336 GWh/year). Yet, all of these forecasts – which are all based on past 1990-2023 actual data – do not reach the total amount forecast by INVENTORY (1,787,858 GWh/year). Our simple regression models are in our paper only used to back up the statement (e.g. in the abstract) "*Past evolution in electricity production since 1990 forecasts lower amounts for 2030. This means Africa is stepping up in planning and constructing additional power plants*." As reviewer 2 points out, many more factors influence actual electricity production increases – such as policy factors (e.g., clean energy policies, power market reforms) and external shocks. A combination of many factors will thus influence individual national electricity production increases (or decreases) in the complex and data-scarce setting of many African countries. Indeed, including factors such as "clean energy policies" and/or "power market reforms" and/or "higher levels of public/private investment" and/or "technological development in energy sources" and/or "decreasing costs of certain renewables" will justify that INVENTORY is much higher than the simple regressions, but this would only confirm the statement that "Africa is stepping up, including due to such factors. Our aim, however, is not to try to model this for 52 different countries, our main aim is to provide an estimate of the real-world situation by means of INVENTORY in these 52 countries, and to also compare this result with the targets of the NDC's (which are quantified targets identified by individual countries). The text and figures throughout the original and revised paper therefore focus on INVENTORY and NDC, to a much lesser extent on the regressions. We think INVENTORY can as such function as a calibration tool or anticipated result for existing or future models.

We add following text in the "discussion" section (Lines 388-409):

> *Different approaches and frameworks exist in energy supply and demand modelling and forecasting, with many review papers published on the matter[39–41]. Their inputs range from socio-economic drivers (GDP, population, …), the effect of energy and climate policies to the effect of external shocks[41] and the engagement of stakeholders. Regarding the latter, there is growing interest in, and demands for, energy modellers to integrate more diverse perspectives on possible and preferred futures into the modelling process. McGookin et al[41] e.g., provide a framework that offers multiple entry points for modellers to incorporate participatory elements either throughout the process or in individual stages. Our assessment indirectly involves stakeholders through using the national NDC's for each country, as these are developed by countries themselves.*
>
> *We do not use sophisticated modelling approaches in our paper. We use three simple regression models (REGR_LIN, REGR_POL, REGR_MVAR), which justify the statement*

that Africa is stepping up in planning and constructing additional power plants. However, the main novel output of this paper is our African power plant inventory (INVENTORY) for the period 2020-2030, which includes 3,139 existing, planned and under construction plants, identifying location, electricity production and fuel type, among others. They represent the real-world situation on main power plants in Africa, as power plants take a long time to construct and our forecast is only for a short time window until 2030. We are confident that no model until now has modelled this situation. Therefore, INVENTORY can function as a calibration tool or anticipated result for existing or future models. By refining input data and/or parameters in existing/future models, INVENTORY provides a real-world forecast up to 2030 for training or finetuning models on the African, national or subnational level.

As INVENTORY is based on data mining and modelling is not the primal focus of our paper, there is less justification for a thorough **uncertainty analysis**. We regard the use of now three different simple regression models (REGR_LIN, REGR_POLY and REGR_MVAR) as a form of uncertainty analysis with respect to using regression based upon past electricity production data (1990-2023) in African countries. We did **add an additional analysis for a different level of environmental flow (EF) protection** to determine water availability, as there are wide variations in the levels of EF protection. We used the methodology of Richter et al in the original paper, resulting in Figure 6 and related text in section 2.2 "*Water use forecasts up to 2030*". This methodology, widely used, can be regarded as a high level of EF protection. We now add an analysis for a methodology considered **low EF protection, i.e. the monthly Q_{95}**, the flow exceeded for 95 per cent of each month. The methodology is described in section "*4.5 Calculation water demand as percentage of renewable water availability for major African basins for INVENTORY*" (Lines 558-561). The results are presented in the new Supplementary Figure 1 and described in section 2.2 (Lines 267-269).

As recommended by Reviewers 2 and 3, we have further developed the discussion section, especially on policy implications. We add following text, with an example from Kenya (Lines 367-387):

Policies on the energy system therefore need to account for different carbon emissions and water intensities of energy sources (Figure 8) as well as local water availability (Figure 6 and Supplementary Figure 1) and the water demand of other sectors. This requires nexus thinking[15], coordination and collaboration between different ministries (such as ministries of energy, environment, water, agriculture/irrigation, …) and stakeholders, thereby aligning policies on energy, water, agriculture, industry and the environment[36].

The Kenyan "Energy Transition & Investment Plan 2023-2050" by the Kenyan Ministry of Energy and Petroleum[37] e.g. outlines the country's path to achieving net-zero emissions by 2050. It aims at decarbonizing the energy system by increasing the construction of renewables (geothermal, hydropower, solar, wind) and ensuring flexibility (batteries, demand response). The plan recognizes that to successfully implement the net-zero ambition in the energy sector, a best practice governance

structure, process, and action plan is required, involving transparency and coordination across the ministries, and sectoral policies that are consistent with national objectives. In the plan[37], the water demand of the energy system and its relation to local water resources is not explicitly mentioned, although Kanda et al[38] confirm that Kenyan water policies should have a central position in WEF related policies. We forecast (INVENTORY) an increase in electricity production from 12,006 to 19,880 GWh (+66%) for Kenya by 2030 compared to 2020. This leads to a 51% increase in water demand, from 275 to 416 Mm³, with hydropower contributing the largest share of this rise. Given that Kenya is already facing water stress, projections like ours should be central to a broader, integrated nexus approach and policy development.

C. Specific comments reviewers

C.1 Specific comments Reviewer 2

This manuscript provides a detailed spatial inventory of power plants in Africa from 2020 to 2030. The topic is highly relevant, given Africa's ongoing challenges with severe power shortages and the additional pressure to align with global Sustainable Development Goals (SDGs) and carbon neutrality targets. Understanding Africa's power plants' development trajectory is critical for formulating sustainable development plans. However, this manuscript falls short in terms of its methodological rigor and the reliability of its conclusions. The lack of methodological transparency, combined with insufficient data support and limited discussions, undermines the overall value of this work. My comments ：

Key Results: The manuscript's main contribution lies in constructing a unique dataset on African power plants. This dataset has the potential to significantly advance research on Africa's energy development and efforts to alleviate energy poverty. However, the predictive results, including carbon emissions, are less convincing due to the simplistic modeling approach and insufficient consideration of critical factors affecting energy systems in Africa.

Originality and Significance: The focus on Africa's power plants is undoubtedly significant, particularly in achieving SDGs, alleviating energy poverty, and addressing carbon neutrality goals. However, the manuscript's potential impact is diminished by the questionable reliability of the predictions and the lack of actionable insights derived from the results.

Answer: Thank you for the appreciation of our work.

1. Data Sources: The sources of the data used in the manuscript are unclear. Transparent and reliable data are essential for reproducibility and credibility. Without clear documentation, it is difficult to assess the validity of the dataset or replicate the study.

Suggestion: Clearly specify the origin of the data, how it was collected, and any preprocessing steps undertaken. Their credibility should be justified if proprietary or new data sources were utilized.

Answer: We agree that we should have provided more clarity on the source of our data. Please see our answer in the part “A. General response to reviewers” of this rebuttal letter

2. Methodological Transparency:

The description of the modeling approach, particularly the polynomial regression used for electricity production forecasting, lacks sufficient detail.

l What specific factors were included in the regression models?

l Were exogenous variables such as GDP, population growth, or international development aid considered?

l Were future scenarios, such as policy changes or technological advancements, incorporated into the analysis?

Without this information, it is impossible to assess the chosen method's appropriateness or the predictions' robustness.

Suggestion: Provide a step-by-step explanation of the modeling process, including the rationale for selecting specific methods and the assumptions made. Include any validation metrics used to evaluate the accuracy of the predictions.

Answer: Please see our answer in the part “A. General response to reviewers” of this rebuttal letter

3. Predictive Reliability:

Using historical growth rates from 1990 to 2020 as the basis for future energy production forecasts is overly simplistic. Many factors influence electricity production, including demand dynamics, policy interventions, and international financing. The lack of consideration for these factors raises doubts about the reliability of the results.

Suggestion: Incorporate a more comprehensive modeling framework, such as integrated assessment models (IAMs) or system dynamics models, to capture the multifaceted drivers of energy production.

Answer: Please see our answer in the part “A. General response to reviewers” of this rebuttal letter

The conclusions drawn in the manuscript lack robustness and reliability due to significant methodological shortcomings:

1. Overreliance on Historical Data in Forecasting

The article uses linear and polynomial regression to predict electricity production, relying solely on historical growth rates from 1990 to 2020 without incorporating exogenous variables or scenario assumptions. This approach overlooks the dynamic drivers of electricity demand (e.g., economic growth, population changes, and industrial restructuring) as well as the influence of policies and technologies on production. Additionally, external shocks such as climate change and geopolitical risks, which could significantly impact Africa’s power systems, are not considered, further undermining the credibility of the forecasts.

Suggestion: Include economic variables (e.g., GDP and population growth rates), policy factors (e.g., clean energy policies, power market reforms), and external shocks in the model. Combine

econometric models with scenario-based analysis to produce more robust forecasts under different conditions.

Answer: Please see our answer in the part "A. General response to reviewers" of this rebuttal letter

2. Lack of Feasibility in Energy Structure Predictions

The article predicts energy structures based on countries' stated targets without clarifying the source of these targets or evaluating their feasibility. For instance, the assumed significant increase in renewable energy shares in economically weaker African countries lacks consideration of the infrastructure and resources required for such transitions.

Suggestion: Clarify the data sources and origin of the targets. Use constraints (e.g., economic capacity, water resource limitations) and conduct cost-benefit analyses or technical feasibility studies to ensure the projections align with reality.

Answer: INVENTORY is the best approximation of reality regarding electricity production in Africa up to 2030. Please see our answer in the part "A. General response to reviewers" of this rebuttal letter

3. Economic and Technical Feasibility

Predictions of extreme increases in electricity production for certain countries (e.g., South Sudan, DRC, Namibia) seem detached from their current economic and technical capacities. Such estimates should be critically evaluated for feasibility.

Suggestion: Compare the predicted growth rates with historical trends and consider incorporating metrics of economic and infrastructural readiness.

Answer: Please see our answer in the part "A. General response to reviewers" of this rebuttal letter

4. Absence of Sensitivity Analysis for Forecasts

The article lacks sensitivity analysis of the model's key assumptions, such as the constancy of historical growth rates, and does not evaluate how results vary under different scenarios. This static assumption approach fails to capture the dynamic nature of real-world situations and does not reveal the model's scope and limitations.

Suggestion: Introduce sensitivity analysis to test the impact of key variables on predictions. Utilize Monte Carlo simulations or scenario analysis to generate various outcomes under different scenarios. Clearly outline the model's limitations and explore potential sources of errors.

Answer: Please see our answer in the part "A. General response to reviewers" of this rebuttal letter

5. Ignoring Water Resource Constraints in Hydropower Expansion Predictions

The article forecasts increased hydropower shares without considering water resource availability or the seasonal variability of water supply. For instance, severe water scarcity in

some African regions may limit hydropower expansion potential, but this issue is not addressed in the study.

Suggestions: Incorporate GIS data to assess water resource availability and its constraints on hydropower development. Discuss the potential ecological and social impacts of hydropower expansion, such as migration and environmental degradation.

Answer: We cover the relationship electricity water demand with available water resources in section “*2.2 Water use forecasts up to 2030*”, by using a state of the art grid level global water availability assessment (spatial resolution of 0.1 degrees or 11.1 km at equator)(Vanham et al). We show this analysis in Figure 6. The methodology is described in detail in section 4.5.

6. Lack of Justification for Overoptimistic Growth Projections

The article predicts extraordinarily high growth rates in electricity production for specific countries (e.g., South Sudan +7443%, Democratic Republic of Congo +2941%). However, it does not explain the feasibility of these results. Given these regions' poor infrastructure and economic conditions, such massive growth appears unrealistic.

Suggestion: To validate their feasibility, compare the projections with current infrastructure, economic capacity, and technological conditions. Offer phased prediction pathways to clarify the consistency of short- and long-term targets. Analyze the investment and technological requirements for such growth to assess their practicality.

Answer: The extraordinarily high growth rates in electricity production are not modelled, but are part of INVENTORY. The extreme increases in South Sudan and DRC are due to the fact that current electricity production is very low and new power plants thereby have such a large incremental effect. This is visible in figures 2 and 3 and justified by the new power plants being constructed in these countries as covered in INVENTORY. For DRC eg, we included in the original paper already text on this (Lines 185-188):

> *For the DRC, primarily hydropower plants are constructed (total 195,823 GWh), with the start of operation between 2024-2030, i.e. the Grand Inga Power Station (152,321 GWh) and the Inga III Power Station (40,075 GWh) on the downstream part of the Congo river, the largest dam project in the world (Figure 3A and 3B).*

References

The quality and timeliness of the references are insufficient. Many cited works are outdated, which undermines the manuscript's ability to position itself within the current state of research.

Suggestion: Update the references to include recent and high-quality studies. This will also help identify potential methodological improvements and provide context for the manuscript’s contributions.

Answer: In the original paper, we had a total of 43 references, of which 35 journal papers. Of those, 18 were published in 2020 or later, with a considerable amount in 2024 and 2023. In the revised manuscript, we added a list of new recent references. In total we now have 53 references, of which 41 journal papers, with 24 papers published in 2020 or later.

1. Main Section:

This section lacks a clear and logical structure, making it difficult for readers to identify key findings and their significance.

Suggestion: Reorganize the main text to ensure clear delineation between methods, results, and discussions

Answer: We have adapted the structure as per rules of Nature Communications

2. Discussion Section:

The discussion is underdeveloped. It fails to adequately address the implications of the findings, their alignment (or misalignment) with previous studies, and their potential applications. For instance, the manuscript does not discuss how its results could inform Africa's energy policies or international climate frameworks.

Suggestion: Expand the discussion to include comparisons with existing literature, practical implications, and potential policy recommendations. Address critical questions, such as how African countries can balance development goals with sustainability.

Answer: We have added text to the discussion as described in the part "A. General response to reviewers" of this rebuttal letter

l Figure 1: Incomplete legend explanations; ensure all abbreviations are defined.

Answer: We added text to the legend

l Figure 2: Misleading title; revise "Energy production" to "Electricity production."

Answer: Thank you for spotting this. We adapted in legend and the figure

l Figure 3: Confusing legend with shapes and colors; simplify by using colors alone to represent energy types.

Answer: Removing shapes would remove information from the figure. We prefer to keep it as is

l Figure 8: Inconsistent labeling of bar charts; ensure all bars are clearly annotated.

Answer: We added text to the legend for clarification

Suggestion: Improve figure clarity by adhering to best practices for scientific visualization. Include a table summarizing scenario differences to improve readability.

Answer: We added a new table 1 with description of scenario's (Line 433). In addition, we added a new table 2 with general overview of data sources (Line 435)

C.1 Specific comments Reviewer 3

The paper titled "Forecast on Africa's power production up to 2030 with related water use and CO_2 emissions" provides a comprehensive and detailed analysis of the future landscape of electricity production in Africa. It effectively highlights the expected increases in electricity generation, water use, and CO_2 emissions from 2020 to 2030, using a robust methodological

framework. The integration of data from various sources, including forecasts based on historical trends, current constructions, and national commitments (NDCs), adds depth and credibility to the study.

Merits:

• Comprehensive Database: The creation of a detailed spatial inventory of 3,139 individual power plants is a significant contribution, providing valuable insights into the future of Africa's electricity production.

• Robust Methodology: The use of multiple forecasting methods (INVENTORY, REGR_LIN, REGR_POL) and the consideration of NDC commitments ensure a thorough and well-rounded analysis.

• Environmental Impact Analysis: The focus on water use and CO2 emissions highlights the environmental trade-offs associated with different energy sources, making the study relevant to policymakers and stakeholders.

• Regional Insights: The detailed breakdown of electricity production and environmental impacts by country and region offers valuable context and highlights disparities that need to be addressed.

Answer: We thank the reviewer very much for this overall very positive assessment

Comments for Improvement:

1. Policy Recommendations: Expanding on specific policy recommendations for achieving electricity production goals while managing environmental impacts would add practical value to the study. Highlighting strategies for integrating renewable energy sources and improving energy efficiency could provide actionable insights for policymakers.

Answer: Please see our answer in the part “A. General response to reviewers” of this rebuttal letter

2. Technical Details: Providing more technical details about the power plants, such as the types of technologies used and their efficiencies, could offer deeper insights into the environmental trade-offs and technological advancements.

Answer: We have listed all information on data sources in the new Supplementary Tables 1 to 6

3. Stakeholder Engagement: Emphasizing the role of stakeholder engagement in achieving forecasted energy production goals and addressing environmental challenges can provide a more holistic view of the pathways forward.

Answer: We have added a brief section on stakeholder engagement in the newly added text on policy implications and modelling approaches in the Discussion section. Please see specifically Lines 391-397

4. Discussion on Uncertainties: Explicitly discussing potential uncertainties and limitations of the forecasts, such as the impact of policy changes, geopolitical factors, and climate variability, would strengthen the credibility and transparency of the study.

Answer: Please see our answer in the part “A. General response to reviewers” of this rebuttal letter

NCOMMS-24-72853

Forecast on Africa’s power production up to 2030 with related water use and CO2 emissions

S.D. Vaca-Jiménez, P.W. Gerbens-Leenes, Bunyod Holmatov, Raphael Vanham, Davy Vanham

First of all the authors would like to thank the editor(s) and the three referees for their time and energy invested.

A. General response to reviewers

Reviewer 3 and 4 are overall positive about our paper.

Reviewer 3 provided a very positive review in the first review round, but did not review the revised version. For the first review round he/she wrote:

The paper titled "Forecast on Africa’s power production up to 2030 with related water use and CO2 emissions" provides a comprehensive and detailed analysis of the future landscape of electricity production in Africa. It effectively highlights the expected increases in electricity generation, water use, and CO2 emissions from 2020 to 2030, using a robust methodological framework. The integration of data from various sources, including forecasts based on historical trends, current constructions, and national commitments (NDCs), adds depth and credibility to the study.

Merits:
• Comprehensive Database: The creation of a detailed spatial inventory of 3,139 individual power plants is a significant contribution, providing valuable insights into the future of Africa's electricity production.
• Robust Methodology: The use of multiple forecasting methods (INVENTORY, REGR_LIN, REGR_POL) and the consideration of NDC commitments ensure a thorough and well-rounded analysis.
• Environmental Impact Analysis: The focus on water use and CO2 emissions highlights the environmental trade-offs associated with different energy sources, making the study relevant to policymakers and stakeholders.
• Regional Insights: The detailed breakdown of electricity production and environmental impacts by country and region offers valuable context and highlights disparities that need to be addressed.

Reviewer 4 is a new reviewer who did not provide feedback in the first review round. Reviewer 4 provides an overall positive evaluation of our paper, while suggesting revisions on certain aspects.

He/she writes:

This study provides spatially detailed inventory of African power plants covering the period from 2020 to 2030. The study fills this gap by creating a database of 3,139 individual plants, including their location, commissioning year, electricity production, and fuel type. The detailed spatial inventory and forecasts provided by this study can inform policymakers and investors about the potential environmental impacts and trade-offs associated with different electricity production strategies in Africa. The study compare the actual power plant construction plans (INVENTORY) with the commitments made in

Nationally Determined Contributions (NDCs) highlighting potential discrepancies between plans and commitments.

The methodology is generally robust and suitable for underpinning the study's assumptions and conclusions. By employing a variety of forecasting techniques, spatial analysis, and considering environmental effects, the study offers a thorough perspective on Africa's prospective energy scenario.

We thank reviewer 4 for this overall evaluation.

Recommendation

Please provide a limitation section of the manuscript and detail all the limitation of the study for transparency

As a very valid and valuable suggestion, Reviewer 4 suggests to add a "Limitation" section to the paper. **We have now added a new section "*4. Limitations*" to the paper**, where we discuss different limitations inherent to any/our modelling study or future forecasting study (Lines 425-481). In this section, specific **comments/concerns of Reviewer 4** as well as **Reviewer 2** are addressed.

Reviewer 2 has reviewed during the first and second review round. He/she had especially doubt on using regression analysis in the first review round, and now again in the second review round.

Although he/she acknowledges that the authors have addressed the comment of the reviewer, even after the authors emphasizing that the main novel part of the paper is INVENTORY, he/she is persistent in the doubt about using regression analysis.

In the new review comments e.g., he/she writes

The author has revised the manuscript in response to the reviewers' comments, supplemented additional content, and refined specific logical structures. This paper predicts electricity production, water consumption, and carbon emissions in Africa up to 2030 based on detailed power plant data. It demonstrates a solid foundation in data collection and preliminary scenario analysis. However, several notable shortcomings remain, including the need to enhance the scientific rigor and precision of the predictions, insufficient articulation of the research significance and practical value, and point-to-point responses that lack specificity and clarity. Particularly, despite extensive efforts in collecting power plant data, the predictive outcomes for the future appear much less than satisfactory.

Authors: Reviewer 2 thus acknowledges "It demonstrates a solid foundation in data collection and preliminary scenario analysis."

Reviewer 2 continues:

This paper employs statistical methods such as linear extrapolation, polynomial regression, and multiple regression to predict electricity production, water consumption, and carbon emissions in Africa before 2030. While these methods are operationally straightforward and offer strong explanatory power, they exhibit several potential limitations when applied to long-term trend prediction. Previous reviewers have raised similar concerns, and although the authors have addressed these comments, my

reservations regarding the results of this paper remain unaddressed. These methodological deficiencies constitute one of the primary factors affecting the overall quality of the paper.

Authors:

- Reviewer 2 writes "While these methods are operationally straightforward and offer strong explanatory power, they exhibit several potential limitations when applied to long-term trend prediction." Reviewer 2 thus acknowledges that our three regression methods are operationally straightforward and offer strong explanatory power. In the same sentence however, Reviewer 2 states that these methods have potential limitations when applied to long-term trend prediction. However, in our paper **we do not do long-term forecasting, we do short time forecasting. We do forecasting from 2024-2030, which is short term.** Indeed, for long time forecasting this could be an issue, but as we do short time forecasting, this is not an issue.
- Reviewer 2 writes that "**Previous reviewers have raised similar concerns**". This is **INCORRECT**. In the 1st review round only Reviewer 2 and 3 provided comments, and **Reviewer 3 did NOT provide any doubt about our use of regression analysis** for our purpose. Reviewer 3 wrote "Robust Methodology: The use of multiple forecasting methods (INVENTORY, REGR_LIN, REGR_POL) and the consideration of NDC commitments ensure a thorough and well-rounded analysis " So it is fully unclear why Reviewer 2 would write this statement. This is NOT a neutral statement, which is a prerequisite for scientific reviewing.

Reviewer 2 continues:

(1) Model assumptions and extrapolation risks. Although the prediction horizon extends only to 2030, a relatively short timeframe, the likelihood of fundamental structural changes in technology or policy remains low. Nevertheless, linear and polynomial extrapolation methods inherently assume that historical trends will persist shortly, potentially obscuring nonlinear fluctuations caused by regional conflicts, extreme climatic events, or delays in commissioning major projects. It is recommended that the authors explicitly articulate the underlying model assumptions and critically evaluate their applicability and limitations for short-term predictions.

Authors:

Reviewer 2 here **contradicts him/herself**. Here reviewer 2 writes that "the prediction horizon extends only to 2030, **a relatively short timeframe**", while in the previous comment Reviewer 2 wrote that our regression analyses "exhibit several potential limitations when applied to **long-term trend prediction**.". As said before, we provide a short-term forecast (2024-2030), NOT a long term forecast

Reviewer 2 continues:

(2) Overfitting and extrapolation risks. Polynomial regression may overfit historical data, leading to considerable uncertainty in out-of-sample predictions. The authors are encouraged to provide a detailed justification for selecting polynomial order and the rationality of the extrapolation interval.

(3) Variable selection and omitted variable problem. The multiple regression analysis relies solely on population and GDP as independent variables, limiting its ability to capture the multifaceted driving mechanisms of electricity development comprehensively. In reality, electricity production is influenced by a wide range of factors, including but not limited to energy prices, investment and financing in power infrastructure, policy incentives or subsidies for renewable energy, trade dynamics in electricity and energy imports/exports, electricity demand structure, urbanization processes, technology penetration rates, and the impacts of climate change and frequent extreme weather events. It is recommended that the authors further justify the rationale behind their variable selection, assess the potential impact of omitted variables on the prediction outcomes, and attempt to incorporate additional key driving factors within the scope of available data to enhance the model's explanatory power and the robustness of its conclusions.
(4) Data dependence and scenario assumptions. The aforementioned methods are highly reliant on the quality of input data and historical trends, exhibiting limited sensitivity to uncertain factors such as future extreme events or policy shifts. To improve the robustness of the results, it is suggested that the authors integrate scenario analysis or sensitivity testing into their methodology. In conclusion, the authors are encouraged to provide a more thorough discussion of the model's applicable premises and potential limitations in the methodology section, supplemented by sensitivity analyses under different scenarios to enhance the scientific rigor of the research conclusions and their policy relevance. In its current form, the predictive framework lacks sufficient credibility

Authors:

To address these comments, we have added information and clarifications in three parts of the paper:

- First, we have added the equations of the three regressions in the section "*5.3 Step 3, estimation electricity generation in 2030 by regression analysis*", with related information and clarification (Equations 3,4 and 5) – Lines 562 to 587. This was also requested by Reviewer 4
- Second, as per the recommendation of Reviewer 4, we have added a new Section "*4. Limitations*" to the paper". In this section, we have dedicated a large part to the use of the three regression analyses REGR_LIN, REGR_POL, REGR_MVAR, including **three new figures Figure 9, figure 10 and Figure 11.** These figures, together with the accompanying text in Lines 436-473, provide justification why the three regression models are justified, in this case with selected examples from electricity production with fuel types in specific countries.
- Third, we add a dedicated paragraph, the last paragraph at the end of "section 3 Discussion", Lines 413-423
- To summarize, these additions provide clear, objective, data-based justification for the use of these three regression methods for the short-time forecast we provide

B. Specific comments reviewers

B.1 Specific comments Reviewer 2

1. The abstract requires further refinement. As the core summary of the entire paper, it currently exhibits the following main issues: First, its structure is somewhat loose, and key content lacks a clear logical hierarchy; second, it does not adequately elucidate the relationships among the research background, methodology, primary findings, and policy implications. It is recommended that the author rewrite the abstract by organizing the content in the sequence of "research background - research design - main findings - policy implications," emphasizing the study's innovation points and the practical significance of its conclusions. It will enable readers to rapidly grasp the essential elements of the paper.

Answer: We have adapted the abstract

2. This paper highlights that developing hydropower in Africa before 2030 will exacerbate regional water resource pressure. However, in predicting the future scale of hydropower, the model does not incorporate water resource sustainability as a prerequisite constraint, relying primarily on historical data and socio-economic variables for trend extrapolation. This approach risks reversing cause and effect logically. Water resources should serve as a fundamental constraint for hydropower planning rather than merely being an outcome of post-assessment. It is recommended that the author further clarify whether and how the availability of water resources is integrated into the hydropower development prediction model. If it is not incorporated, the author is advised to supplement the discussion regarding the impact of this assumption on the conclusions or explore the use of coupled modeling and other methods to more scientifically reflect the interrelationship between water and electricity, thereby enhancing the rationality of the conclusions and the feasibility of policy recommendations.

Answer: First of all, INVENTORY are the power plants that are under construction or are planned for construction up to 2030, irrelevant of whether they are in water scarce areas or not. Second, we evaluate the relationship between the water use of these power plants to water availability, which is the standard way to provide information on water scarcity/water constraints. This is clearly shown in figure 6 of the paper, with accompanying text in section 2.2. We clearly describe this methodology in section "*5.6 Calculation water demand as percentage of renewable water availability for major African basins for INVENTORY*". The data and methodology we use for water availability and environmental flows are state of the art and in highest spatial resolution. We use a state of the art indicator on water scarcity for this assessment, i.e. SDG indicator 642 on water stress, which relates water use to environmentally available water resources (water resources minus environmental flows). All UN member states need to report on this indicator, it is THE globally accepted indicator to use. We use a state of the art global hydrological model. Our assessment is state of the art. Reviewer 2 already questioned our approach in the first review round. Why the reviewer keeps on questioning this approach is unclear.

4. This paper presents predictions for data beyond 2023. Has the actual data for 2024 been officially released? If the 2024 data is publicly available, it would be necessary to compare the model's predicted results with the latest empirical data to validate its accuracy and applicability. Such comparative analysis not only aids in evaluating the model's predictive capability but also strengthens the credibility of the research conclusions and their policy relevance. It is recommended that the author incorporate relevant analysis or discussion in the revised manuscript.

Answer: The strength and novelty of our paper has been built on the development of INVENTORY until 2030. The data for 2024 in INVENTORY have not been changed, we submitted our paper at the end of the year 2024. INVENTORY is based on data mining, not on modelling

5. The discussion on the significance of the research in this paper remains somewhat inadequate. Two key issues warrant further consideration and elaboration: (1) The practical necessity of short-term prediction. Africa has consistently faced structural challenges, including insufficient investment, prolonged project implementation cycles, low electricity access rates, and significant regional disparities in power infrastructure development. Given that this paper focuses its predictions solely on 2030—only five years away—it is essential to clarify the rationale and urgency of such short-term forecasting. (2) Practical policy implications. While this paper provides a spatial inventory of African power plants and conducts relevant predictions, it is crucial to address the question of "so what." Does the research content offer tangible value? It is recommended that the author delve deeper into the specific policy implications of the prediction results. For instance, which countries require accelerated planning efforts? How can the future power structure be optimized under the current constraints of water resources and carbon emissions? Can the findings serve as risk warnings or decision-support tools for governments, regional organizations, and international institutions?

Answer: We do not understand the comment of the reviewer on "While this paper provides a spatial inventory of African power plants and conducts relevant predictions, it is crucial to address the question of "so what." Does the research content offer tangible value?" Again, our INVENTORY is the main novelty of the paper. We provide a database of 3,139 power plants, all individually checked on satellite images for correct coordinates and all with multiple information such as fuel type, capacity, water use , CO_2 emissions etc. No such database in this level of spatial detail is available. We provide this database open access, for any stakeholder inside or working on Africa to use. Currently FAO's Aquastat not even provides data on water use for electricity for most African countries on the national level up to the year 2022. The level of spatial detail and future forecast of actual power plants under construction and planned, is unprecedented. We think thereby the answer to the questions "so what" and ." Does the research content offer tangible value?" is quite straightforward. We describe in detail our results for the overall African level and to a certain extent we cover individual fuel types and countries (Figures 2 to 11). However, it is impossible to go into the discussion in detail of every individual country and power plant, that would imply that we add many more pages of text and figures to the paper. We are restricted in Nature communications on amount of text and figures.

B.2 Specific comments Reviewer 4

This study provides spatially detailed inventory of African power plants covering the period from 2020 to 2030. The study fills this gap by creating a database of 3,139 individual plants, including their location, commissioning year, electricity production, and fuel type. The detailed spatial inventory and forecasts provided by this study can inform policymakers and investors about the potential environmental impacts and trade-offs associated with different electricity production strategies in Africa. The study compare the actual power plant construction plans (INVENTORY) with the commitments made in Nationally Determined Contributions (NDCs) highlighting potential discrepancies between plans and commitments

Answer: We thank the reviewer very much for this overall very positive assessment

General Comments

1.Method used in the paper is absent in the abstract.

Answer: Thank you for this comment. We adapted the abstract and included the method.

2.There are discrepancies between the numbers in the Excel file and those reported. For instance, line 85 mentions 3,246 individual power plants, but the Excel file indicates a different figure. Same as line 152. Please verify these and other reported numbers.

Answer: Thank you for this comment. Indeed, there were inconsistencies, due to the fact that in INVENTORY the power plants for NDC were added. To resolve this issue, we split the power plants of INVENTORY (provided as Supplementary Data 1) and the power plants for NDC (provided as New Supplementary data 6). The values are now correct.

3.Line 248; Kenya, not Kenia.

Answer: Kenia changed into Kenya

4.Kindly note that the statements found in lines 285 to 287 contradict the information presented in Figure 7. Please review and revise lines 277 to 289 to enhance clarity.

Answer: Our description of the information on Figure 7 was too cryptic. We have made changes to the text (now Lines 281-295) for clarity

5.Line 307; please review the conceptual use of energy and electricity.

Answer: Thank you for this comment. Indeed, this paper is about electricity, not energy in general. However, policy plans include not only electricity but energy in general. Where needed, we changed energy into electricity.

6.Line 381; The abbreviation WEF has just appeared. Kindly review and clarify its meaning.

Answer: We added Water-Energy-Food (WEF) nexus

Data reliability and completeness:

The research extensively utilizes publicly accessible and secondary data sources to gather information on power plants. However, this data may contain inconsistencies or omissions, particularly concerning smaller nations or recent projects. Such issues could result in inaccurate estimations of electricity generation and its associated effects.

Take Ghana as an example; the authors utilized data from the IEA and other secondary sources. Nonetheless, the power generation figures reported by Ghana's Energy Commission for 2020 and other years show a notable discrepancy when compared to the IEA's 2020 data for Ghana.

Answer: For INVENTORY, we applied publicly available data sources to come up with plants per country, fuel type and location. However, most of the time only installed capacities are given, not generated electricity. We therefore downscaled national production data per fuel type from the IEA and calculated electricity per unit of installed capacity per country per fuel. These factors used are observed in the column F of the Supplementary Data 1 (INVENTORY) as well as the newly added Supplementary Data 6 (NDC)

Recommendation

I suggest using primary data at the national or country level for power generation across all African nations, except when such data is unavailable, in which case the IEA and IRENA database can be used

Answer: We took national data from the IEA because the IEA bases its data on national governmental data. Sometimes these data are adjusted, and are not exactly the same as the national ones. For Ghana for example, the IEA reports 19,714 GWh, whereas Ghana's Energy Commission reports 20,170 GWh, a difference of 2.3%. We argue that using only one source for all countries gives a reliable insight into electricity production for Africa

When gathering data on electricity generation by country, did you consider the electricity trade between African nations? This aspect could significantly impact the study's findings, particularly regarding CO_2 emissions and water usage and consumption.

Recommendation

I recommend that actual electricity generation are accounted for by considering power trade among neighboring African countries.

Answer: Our study aimed to assess water use and CO_2 emissions related to electricity production per country. Indeed, if countries import electricity, the water use related to consumption is located in another country. However, water is used at the place where electricity is generated, not consumed. We only made the assessment for production so that we could estimate the effect of water use on water scarcity.

This was already expressed in the methodology Lines 605-607:

> *For the calculation of freshwater withdrawal and consumption, we only include the operational stage, excluding water in the supply chain.*

Methods

The methodology is generally robust and suitable for underpinning the study's assumptions and conclusions. By employing a variety of forecasting techniques, spatial analysis, and considering environmental effects, the study offers a thorough perspective on Africa's prospective energy scenario. Although the approach has been indirectly assessed by comparing it with historical patterns and policy commitments, it could benefit from further validation measures.

Answer: Thank you for the overall appreciation of the methodology we use

Method weakness

The water withdrawal and consumption calculations use average values for different power plant types. This may not capture the variability in water use due to specific local conditions, cooling technologies, or plant designs.

Recommendations

Please use specific values for water withdrawal and consumption for different power plants.

Answer: One of the strong points of our paper is that we do use a very specific method that accounts for localized and specific water intensities for technology. For this, first we studied the satellite images for all thermal power plants to assess the cooling type, i.e., cooling tower, once-through or dry cooling, and type of water, i.e. freshwater or salt water. Then, we used differentiated water withdrawal and consumption factors for each power plant depending on the climate, so we are considering differences based on different weather requirements of water. For hydropower we calculated water evaporation per plant depending on the reservoir surface areas, climate or other infrastructure. We consider that this is the most specific calculation of water usage found in the literature. This is observed in the Supplementary information, Tables 4-6.

We admit that we did not describe this enough in the paper, so have highlighted this more in the method section – e.g. Lines 510-512:

> *We studied satellite images to confirm the coordinates of all power plants. We also used these satellite images for all thermal power plants to assess the cooling type, i.e., cooling tower, once-through or dry cooling, and type of water, i.e. freshwater or saline water.*

And Lines 612-625:

> *Freshwater withdrawal and CO2 emissions were calculated in the same way using the specific freshwater withdrawal data of energy source s in climate c using the same methodology as the one used in Gerbens-Leenes et al. (2024) and CO2 emissions from the National Renewable Energy Laboratory (NREL), 2024. For this calculation differentiated factors for each power plant, depending on the climate and type, were used.*
>
> *The water intensities are based on following climate zones, as obtained from Kottek et al44: Tropical Rainforest (Af), Tropical Monsoon (Am), Tropical Savannah (Aw), Arid Steppe Hot (BSh), Desert Hot (BWh), Humid subtropical (Cfa), Temperate Without dry Season (Cfb), hot-summer Mediterranean (Csa), warm-summer Mediterranean (Csb), Dry-winter humid subtropical (Cwa), Dry-winter subtropical highland (Cwb).*
>
> *For hydropower we calculated water evaporation per plant depending on the reservoir surface areas, climate or other infrastructure.*
>
> *All relevant information and data are included in Supplementary Tables 4 to 6 in the SI.*

The study doesn't appear to account for potential improvements in power plant efficiency or new technologies that could emerge by 2030. This could affect both electricity production estimates and associated water use and CO2 emissions.

The INVENTORY forecast assumes that all planned and under-construction power plants will be completed by 2030. This may not account for potential delays, cancellations, or changes in energy policies. The other forecasting methods (REGR_LIN, REGR_POL, REGR_MVAR) are based on historical trends, which may not accurately reflect future developments, especially in rapidly changing energy landscapes

Recommendation

Please provide a limitation section of the manuscript and detail all the limitation of the study for transparency.

Answer: This is a very valid and useful suggestion, to include a “Limitation section”.

We have now added a new section “*4. Limitations*” to the paper, where we discuss different limitations inherent to any/our modelling study or future forecasting study (Lines 425-481).

With regard to the comment on efficiency: In this study, we downscaled national electricity production data to installed capacity, generating information on electricity production per unit of installed capacity (MWh/MW). When new thermal power plants are built, probably the efficiency increases. However, based on the limited timeframe of the projection, we assume there is no significant change in efficiency. Moreover, power plants have a long lifetime, and the fraction of new thermal installations will be small, so differences on a national scale will also be small. Moreover, electricity production expansion is mostly done using renewables like hydropower, solar and wind.

We have added relevant information in the section “*4.Limitations*” Lines 474-481

Recommendation for method presentation

The method should include all equations related to the regression analysis. Additionally, please supply a list of the variables used in the regression analysis along with their definitions.

Answer: This is a very valid recommendation. We have included the regression equations and associated information in the section "*5.3 Step 3, estimation electricity generation in 2030 by regression analysis*" Lines 562-586

Recommendation for method improvement

The method doesn't delve into the economic feasibility or cost-effectiveness of different power generation options, which could significantly influence actual development paths. Please consider economic parameters as well.

Answer: The strong part of our study is the forecast electricity production based on the inventory of new power plants that are defined according to the expansion plans of every country. In this case, there is no need to include cost-effectiveness of different power generation options as they are already considered in the government's plans. The other forecasting methods are simply used to provide a range of possible increases of water and carbon footprints, and not to identify possible paths of electricity production. The more-detailed integrated or econometric methods are often used to calculate the electricity demand of countries so other models can identify the best cost-effective technologies to be included in the mix. We are prioritizing simplicity over complexity of the model as our focus is to understand how the actual plans will affect water and carbon footprints, and not to indicate possible mixes that will solve a problem (either water or carbon intensities). We have included a relevant discussion in the last paragraph of section "*3. Discussion*" Lines 413-423

NCOMMS-24-72853C-Z

Forecast on Africa's power production up to 2030 with related water use and CO2 emissions

S.D. Vaca-Jiménez, P.W. Gerbens-Leenes, Bunyod Holmatov, Raphael Vanham, Davy Vanham

First of all the authors would like to thank the editor(s) and the two referees for their time and energy invested.

A. General response to reviewers

Our paper, first submitted on the 10th of November 2024, has now gone through different review rounds with several different reviewers.

In this review round, two reviewers have provided comments, reviewer 2 and 4. Both are overall positive about our paper.

Reviewer 2 writes:
This study presents a highly detailed spatial inventory of operational, under-construction, and planned African power plants between 2020 and 2030, based on an intensive data mining effort that identifies 3,139 individual facilities. We appreciate the authors' substantial efforts in revising the manuscript and their thoughtful responses to the reviewer comments.

Reviewer 4 writes:
The authors have modified the article significantly and responded very well to the comments. Hence may be accepted in my opinion if other reviewers/ editorial review agreed

B. Specific comments reviewer 2

1. Enhance the Introduction Section

While the introduction provides some background and motivation, it remains underdeveloped in several crucial aspects. The authors are encouraged to revise and expand the introduction along the following lines:

(1) The current manuscript lacks engagement with related academic studies. Have other scholars conducted similar work forecasting energy production, water use, or CO_2 emissions in Africa or other regions? What are the major findings and methodological approaches in those studies? How does this work differ from or improve upon them? This context is critical to help position the contribution of the present study and underscore its originality.

Authors: We have now added a paragraph on existing literature Lines 137-153

(2) The introduction would benefit from a paragraph summarizing the most important and surprising findings of the study. For example, differences between the INVENTORY and NDC projections on renewable energy sources could be highlighted here.

Authors: This information can be found in the abstract, results and discussion sections, as per Nature journal instructions.

(3) Compared with existing studies, what contributions does this research make? The authors need to add a paragraph explaining, from multiple perspectives, how this study extends the existing literature and clarify why these extensions are important.

Authors: This is a very relevant comment. We have now added a paragraph on existing literature and the position of our study within the existing literature, Lines 137-153

(4) To improve readability and guide the reader through the paper, the authors should add a paragraph at the end of the introduction that outlines the structure of the remaining sections.

2. Reorganize the Overall Structure of the Manuscript

The structure of the paper is not well-organized. Several major revisions are suggested:

(1) The manuscript lacks a dedicated literature review section. Moreover, there is minimal discussion of existing studies throughout the paper. This weakens the justification for the study and makes it difficult to assess its novelty. A stand-alone literature review or a dedicated subsection within the introduction would be highly beneficial.
(2) Currently, the methods section appears later in the manuscript (Section 5), while results are presented earlier (Section 2). This ordering is counterintuitive and should be reversed. A more standard and reader-friendly structure would follow the format: Introduction – Literature Review – Methods – Results – Discussion – Conclusion.
(3) There is no distinct Conclusion and Policy Implications section. Although the authors summarize the findings in Section 3 "Discussion," discussion and conclusion are not the same. In the discussion section, the authors should connect the key findings of this study to existing literature or real-world contexts, explaining their significance and validity. In the conclusion and implications section, the authors should clearly present the main findings and provide targeted implications or recommendations based on these findings.
Authors: As a reply to this list of comments, the authors would like to stress that they follow the standard structure of Nature Communications papers, which is provided in the guide to authors. We therefore do not have the possibility to follow the recommendations from the reviewer, which are recommendations not in line with the requirements of Nature Communications.

From the author instructions webpage of Nature Communications
https://www.nature.com/ncomms/submit/article

> The maximum title length should be 15 words. The abstract — which should be no more than 200 words long and contain no references — should serve both as a general introduction to the topic and as a brief, non-technical summary of the main results and their implications.
> The main text of an Article should begin with a section headed Introduction of referenced text that expands on the background of the work (some overlap with the abstract is acceptable), followed by sections headed Results, Discussion (if appropriate) and Methods (if appropriate). The Results and

Methods sections should be divided by topical subheadings; the Discussion should be succinct and may not contain subheadings.

We did add a concise literature review paragraph in the introduction section, Lines 137-153. But as per Nature Communications instructions, this should fit within the introduction section.

It also needs to be stressed that there is a limitation on words as per journal instructions, where we about reached the maximum amount of words, also due to the recommendation of different reviewers to add more information on different topics to the paper.

3. Deepen the Analysis of Key Results

The results section is currently structured around three forecasts: electricity production, water use, and CO_2 emissions. While this is logical, the treatment of these results is too descriptive and lacks analytical depth. As it stands, the manuscript reads more like a technical report than a scholarly article. For example:
"For the renewables, considerably more power is produced from hydropower in INVENTORY (517,501 GWh) compared to NDC (378,598 GWh). For solar it is the opposite (24,570 GWh and 179,645 GWh, respectively), as well as for wind (30,685 GWh and 109,627 GWh, respectively)."
This is a very interesting finding, but it is not sufficiently explained or contextualized. Why is hydropower overrepresented in INVENTORY compared to NDCs, while solar and wind are underrepresented? Are these patterns a result of historical investment decisions, differences in construction timelines, policy priorities, or financing constraints? The authors should unpack these patterns and discuss the implications for energy policy and climate planning in Africa.
Authors: We thank the reviewer for this valuable comment. We have added information in the relevant section "Discussion", i.e. within Lines 378-401

4. As the authors mention in the study's limitations section: "The INVENTORY forecast assumes that all planned and under-construction power plants will be completed by 2030. This assumption is made based upon the relatively short time window until 2030 and the average planning and construction time for power plants." The assumption may bias the forecasts in favor of slower-to-build technologies like hydropower and thermal plants, while underestimating the role of fast-deploying renewables such as solar and wind. Construction times for utility-scale solar PV and onshore wind are typically less than 12 months, making them highly scalable over the short term. This could partially explain why solar and wind outputs in INVENTORY fall below those projected in NDCs, and why hydropower is overrepresented. Given Africa's increasing commitment to low-carbon development, these fast-to-deploy technologies may witness significant growth in the near term—an aspect not fully captured in the INVENTORY-based forecast.

Authors: This is indeed a valuable comment. We have incorporated this information in the relevant section "Discussion", i.e. within Lines 378-401

NCOMMS-24-72853D

Forecast on Africa's power production up to 2030 with related water use and CO2 emissions

S.D. Vaca-Jiménez, P.W. Gerbens-Leenes, Bunyod Holmatov, Raphael Vanham, Davy Vanham

First of all the authors would like to thank the editor(s) and all past referees for their time and energy invested. All comments and suggestions have helped us to substantially improve our paper compared to the first submitted version.

A. General response

Our paper has been reviewed by several reviewers, who have generally given very positive feedback that has substantially improved our paper through different review rounds.

Reviewer 2 has provided some critical comments on the last submitted version of the paper. We think that by addressing these comments reviewer 2 helped us to improve our analysis.

Before this review, Reviewer 2 has at several previous review rounds provided critical comments, many of which we have already addressed in previous revisions of our paper.

More specifically, **on the request of reviewer 2; in past revision rounds, we**:

- added clarification text in different parts of the paper
- added clarity on data sources, by a very detailed listings of all data sources in the form of supplementary tables in the Supplementary information (combined covering 279 references). Additionally, we added a new general overview Table 2 in section 4 "Methods" of the main paper, in its introductory paragraphs. As such, we provide full transparency on all data sources used in this paper.
- added an additional regression analysis, i.e. a Multivariate regression (REGR_MVAR) using population and GDP as variables. We also added the equations of the three regressions and included a detailed discussion on the use of these three regression (including three new figures Figure 9, Figure 10 and Figure 11)
- accounted for uncertainty, by adding an additional analysis for a different level of environmental flow protection
- added text on existing literature in the introduction section, to position our work in the current literature

In this review round, reviewer 2 provides several additional comments. We address all comments of reviewer 2, by:

- doing additional analyses (an ARIMA analysis and uncertainty analysis) and
- providing additional information, clarifications and discussion.

These are discussed in detail in the following.

B. Comments reviewer 2 this review round

1. I previously noted that the manuscript lacks engagement with related academic studies. It remains unclear how this work positions itself relative to prior research on forecasting energy production, water use, or CO_2 emissions in Africa or other regions. Although the authors added some discussion of alternative databases, they still fail to review the major findings and methodological approaches of comparable studies or explain how their work differs or improves upon them. This omission leaves the contribution poorly contextualized and weakens the scholarly significance of the paper.

2. I asked the authors to clarify the study's contribution compared with existing literature, emphasizing methodological, content-based, and theoretical innovations. Instead, the revision merely highlights differences from other databases and provides no substantive explanation of how this research extends existing knowledge or why any extensions would be important. Without a clear statement of novelty, the paper struggles to justify publication.

4. Despite I earlier requested for a thorough literature review, the authors only inserted a short discussion (Lines 137–153) about other databases, which is insufficient. The manuscript still lacks a substantive review of relevant empirical and theoretical studies, leaving readers without the necessary background to understand how this research fits into or advances the field.

Answer: We have provided the following focused text in the Introduction section (Lines 156-172), which describes, by using targeted references, why our analysis and especially INVENTORY has a very high degree of novelty:

> *We thereby provide our INVENTORY assessment for the years 2020 up to 2030 in unprecedented spatial resolution, with associated water use and CO_2 emissions for each power plant. In general, sectoral water use data, including for electricity, are very scarce for African countries[28]. For many countries, data are even lacking on the national level. A persistent lack of reliable water data and information is a widespread constraint on water management in the global South[29]. For detailed water management and water scarcity assessments, accounting for different water demand stakeholders such as electricity, spatially detailed data are a necessity[18,22,30,31]. The spatial detail of INVENTORY is unprecedented. Such an assessment has not been made available in the literature up to this date.*
>
> *In addition, we provide all data open access, for any stakeholder to use freely. This is of high importance for researchers and stakeholders working in Africa, as they often lack the funds to purchase high quality data and assessments. Some companies manage databases on existing power plants[32], however their data usage and replication are restricted and not available under a creative common license[24]. There is therefore a lack of open-access, reliable, georeferenced information on existing and proposed power plants covering Africa, which limits ongoing research efforts on the sustainable development of electricity production and constraints a science-based discussion among stakeholders in the decision process.*

In addition, we added a new Supplementary Table 1 in the SI (to which we refer in Line 145), which shows how INVENTORY is superior to existing databases in its level of detail. This table is also shown here in the rebuttal letter:

Supplementary Table 1: Comparison of data entries (number of power plants and capacity in MW) between our database, WRI's Global Power Plant Database[1] and the Renewable Power Plant database for Africa (RePP Africa)[2]

	This study				WRI's Global Power Plant Database[1]		Renewable Power Plant database for Africa (RePP Africa)[2], study published in 2023			
	year 2022		year 2020		Year 2020 (most recent year)		year 2022 (most recent year)		Year 2020	
	Number	Capacity (MW)	Number	Capacity (MW)	Number	Capacity (MW)	Number	Capacity (MW)	Number	Capacity (MW)
Oil	1,061	23,272	1,054	22,265	102	8,425				
Coal	51	52,202	49	49,807	31	45,097				
Natural gas	358	125,512	343	119,263	134	64,293				
Hydropower (reservoir+ROR)	581 (195 reservoir +386 ROR)	41,864	561 (183 reservoir +378 ROR)	36,892	163	30,399	336 (178 reservoir+121 ROR+37 undefined)	37,849	331 (178 reservoir+118 ROR+35 undefined)	37,070
Wind	100	7,859	83	5,646	42	4,499	117	9,024	102	7,631
Sun	398	7,072	299	6,150	129	4,893	348	8,313	282	7,163
Biomass	127	2,066	121	1,958	15	320				
Geo	9	896	8	813	7	761				
Nuclear	1	1,860	1	1,860	1	1,800				
Waste heat	15	951	15	951	7	46				
**total**	**2,701**	**263,554**	**2,534**	**245,604**	**631**	**160,533**				

The other two databases have much fewer entries for Africa than the ones we include. They also do not include water use nor CO_2 emissions. The Global Power Plant Database only includes existing power plants whereas RePP Africa includes some future renewable power plants. The level of (spatial) detail of our INVENTORY is unprecedented, while being made available 100% open access.

3. To improve readability, we recommended adding a paragraph at the end of the introduction outlining the structure of the remaining sections. The authors ignored this suggestion.

Answer: We added following paragraph (Lines 174-182):

Our manuscript is organised in the following way:

- *A section "Results" provides results on the electricity production forecasts up to 2030, the water use forecasts up to 2030 and the CO2 emissions up to 2030*
- *The section "Discussion" first generally discusses the results, followed by a discussion on policy implications and recommendations, followed by a brief discussion on limitations and uncertainties*
- *The section "Methods" provides an overview on the methodology, presented in different steps and subsections*

5. I previously urged the authors to offer targeted policy recommendations based on their findings. They have not incorporated any such guidance. Without actionable policy implications, the study's practical relevance is greatly diminished, limiting its value to policymakers and stakeholders.

Answer: We added the following targeted policy recommendations in the Discussion section, while referring to selected literature (Lines 443-467):

Targeted policy recommendations can be summarized as follows:

- *Policies on the electricity system require nexus thinking. They need to account for different carbon emissions and water intensities of energy sources (Figure 8) as well as local water availability and the water demand of other sectors. Hydropower is vulnerable to increased*

and intensified drought events due to climate change, requiring a mix of energy source investments. Additional expansion in the low-cost energy sources solar and wind is a win-win in many African settings. Additional stepping up of these sources until 2030 and beyond may reach NDC's

- *Coordination and collaboration between different ministries and stakeholders is required, thereby aligning policies on energy, water, agriculture, industry and the environment*[49]
- *It is essential that African countries secure investment/financing for their planned and under construction power plants, so that they are not delayed or cancelled. Financing can occur through a mix of public and private funding, from the private sector*[52]*, multilateral institutions such as the World Bank or African Development Bank*[53]*, bilateral development agencies or others.*
- *Integrated solutions are required, including the construction of a mix of different (renewable) energy sources*[54]*, battery storage*[54]*, the establishment of a reliable national grid including transnational electricity sharing (international power pools)*[46] *as well as small scale localized solutions*[46]
- *Policies need to address cooperation and river management across borders*[55]
- *Policies should be data and science based. Our INVENTORY, a first openly accessible and location specific dataset for the years 2020 to 2030 is fundamental for the development of an integrated sustainable renewable energy mix. Countries need to share high-quality data, including data about their power plants and sectoral water uses*

7. The forecasting approach relies only on three basic regression models—a linear regression (REGR_LIN), a polynomial regression (REGR_POL), and a multivariate regression with population and GDP as predictors (REGR_MVAR). Such simple extrapolations are insufficient for a study of this scale and importance. The authors should benchmark these results against more advanced forecasting techniques (e.g., time-series models like ARIMA or machine-learning methods) to demonstrate the robustness and accuracy of their projections.

Answer: In a previous revision round, we added a lengthy section at the end of the discussion section indicating why the use of these three regression methods is justified. We had added three new figures to additionally justify this – Figures 9, 10 and 11. Reviewer 4 gave following feedback on this addition: *The authors have modified the article significantly and responded very well to the comments*. The use of three different regression methods accounts for uncertainty in regression analysis.

However, **we here take the advice of Reviewer 2** and **add an analysis with the time-series model ARIMA** (Autoregressive Integrated Moving Average).

By doing so, we use more detailed – annual – time series on electricity production from 1990 until 2019 for each country and fuel type, compared to the original 5-year time step from 1990-2015 we used in the original analysis. This means that apart from the new ARIMA analysis, **all three regression analyses were updated by using annual data**, thereby increasing their level of detail and accuracy.

We therefore updated Figure 1, but also the Figures 9, 10 and 11 (including Lines 533-560). We find however that the new regression analyses do not differ much from the original regression analyses. We updated the values, but the differences are so small that they do not change anything on the results or conclusions of our paper.

We make adaptations throughout the manuscript to accommodate the additional ARIMA analysis.

We show in a revised Figure 1, that the ARIMA forecast for 2030 adds up to 1,280,169 GWh, which is about in the middle of the range between the REGR_LIN value (1,157,485 GWh) and REGR_MVAR value

(1,155,210 GWh) on the one hand and the REGR_POLY value 1,414,231 GWh on the other hand. These values are adapted in Lines 191-192. Important is that also the ARIMA forecast is well below INVENTORY, stressing that our statement “Africa is stepping up” is also valid with respect to historical data analysed with ARIMA.

We add a description on the methodology for the ARIMA analysis in the Methodology section (Lines 678-715)

To estimate the ARIMA for the technologies, an automated batch processing script was used to ensure that each dataset—regardless of country, technology, or data format—was processed identically and reproducibly. This open access Python script is added to the paper as presented in the section “Code availability”.

6. I raised concerns about a key modeling assumption: the INVENTORY forecast assumes all planned and under-construction plants will be completed by 2030. This biases the results toward slower-to-build technologies like hydropower and thermal plants and underestimates fast-deploying renewables such as solar and wind, which typically require less than 12 months for construction. While the authors acknowledge this issue in passing, they have not adjusted their model to account for it. This uncorrected assumption likely skews the forecast and weakens the credibility of the results.

8. The projections also lack a sensitivity analysis to test how results respond to key assumptions, such as variations in plant construction timelines. Without examining how uncertainties in these factors affect the outcomes, the reliability of the forecasts is significantly reduced, and readers cannot assess the stability of the conclusions under alternative scenarios.

Answer: INVENTORY is not the result of modelling, it is the result of an intensive data mining effort. To emphasize this even more, we have added a Supplementary Figure 1 showing the different steps of the methodology workflow in the paper.

The comment of Reviewer 2 is valid for INVENTORY though, and we have partly discussed this fact in our past revision – which Reviewer 2 acknowledges as “While the authors acknowledge this issue in passing, they have not adjusted their model to account for it.”

To properly address this, we have accounted for this fact in an uncertainty analysis. We now assembled all uncertainty analyses in our paper in a new Methodology Subsection “4.7 Uncertainty analysis”. Please see Lines 794-810:

> *To account for uncertainty for future power plants up to 2030 in INVENTORY, we account for delays in power plants becoming operational. We assume that for solar power plants and wind farms, with their relatively short planning and construction time, a delay of 1 year occurs. For all other power plants we assume a delay of 2 years. In addition, we select planned nuclear power plants and large hydropower plants due to their long planning and construction times, and exclude them in case of a substantial possibility for a delay, based on recent information. As such, we quantify electricity production by 2030 for an INVENTORY analysis with substantial delays.*
>
> *For our assessment on the water demand as percentage of renewable water availability for major African basins for INVENTORY, we also account for uncertainty: we conducted the same analysis for an EF measure representative for minimum flow recommendations, i.e the monthly Q_{95}, the flow exceeded for 95 per cent of each month[30, 59]. As such, we provide a range between a high (EFs by Richter et al[33]) and low (Q_{95}) EF protection level. The results are shown in Supplementary Figure 2.*

In addition, we account for uncertainty in future electricity production forecasting up to 2030 based upon past time series data (from 1990-2015 as well as on the database of 2020 and its update for 2023), by using three different regression analyses as well as ARIMA.

The results of this particular uncertainty analysis are added in the Discussion section, Lines 509-522:

Assuming in INVENTORY delays of 1 year for solar power plants and wind farms to become operational, for all other power plants 2 years, and accounting for potential delays in nuclear and large hydropower plants, decreases the electricity production forecast from 1,787,858 GWh in 2030 to 1,504,848 GWh. Much of this decrease is due to the assumption that the large Congo river hydropower plants are delayed, i.e. the Grand Inga Power Station (152,321 GWh) and Inga III (40,075 GWh). Other large hydropower plants include the Mambilla power plant in Nigeria (11,777 GWh) and Mphanda Nkuwa in Mozambique (10,710 GWh). We also assume the two new Nigerian nuclear power plants to be delayed (17,636 GWh). This much delayed version of INVENTORY thus only adds up to 1,504,848 GWh, an amount still higher than the highest time series regression analysis (REGR_POL 2030, 1,414,231 GWh, Fig 1). Even for the delayed version of INVENTORY, it can be confirmed that Africa is stepping up in electricity production. As we most probably underestimate solar power plant and wind farm projects in INVENTORY for the later years up to 2030, our statement that Africa is stepping up remains valid.

NCOMMS-24-72853E-Z

Forecast on Africa's power production up to 2030 with related water use and CO2 emissions

S.D. Vaca-Jiménez, P.W. Gerbens-Leenes, Bunyod Holmatov, Raphael Vanham, Davy Vanham

First of all the authors would like to thank the editor(s) and the referee for their time and energy invested. All comments and suggestions have helped us to substantially improve our paper.

A. Editorial requirements

In line with specific requirements listed by the editor, in this revised version we have:

- Removed the numbers before the subheadings
- removed bullet points in the text
- adapted the figures so that the figure panels are labelled with a, b, c instead of capitalized A, B, C
- redesigned supplementary figure 1 by ensuring a white background and removing unnecessary colour, as listed under https://www.nature.com/ncomms/submit/how-to-submit
- put all Supplementary data in one Zip file

B. Comments reviewer 2 this review round

The revision is substantially improved and is responsive to most of our previous concerns. At this stage, I believe the manuscript would benefit from further refinement in several areas. Addressing the points below would further improve the overall quality, clarity, and scholarly positioning of the paper.

Answer: We thank the reviewer for the overall assessment "*The revision is substantially improved and is responsive to most of our previous concerns*". We are happy to see the positive evaluation of our manuscript

Introduction: The first two paragraphs mainly emphasize why Africa's power production is an important topic, after which the Introduction quickly moves to describing the present study. I recommend inserting a dedicated paragraph (or two) before introducing the study design to summarize the current state of research in this area and to clarify what remains insufficient or unresolved in the existing literature. This would help readers understand more clearly why the present study is necessary and what specific gap it fills.

Introduction: Please include a short paragraph that explicitly summarizes the main contributions relative to prior studies. A clear contribution statement will help readers quickly grasp the manuscript's novelty and its specific value to the field.

Answer: We have added following Lines as new third paragraph (Lines 48-58), while referring to existing studies:

> *African countries need to invest in future power plant expansion in a water resilient way, accounting for CO2 emissions and the wider energy-water-climate nexus[3–5]. Yet for informed decision making, there is a need for reliable, findable, high-quality, open access and spatially*

distributed power plant data with associated water use and CO_2 emissions amounts. Currently there is a persistent lack of such data throughout Africa for the current situation as well as for future projections[6]*. Some data are available*[7–10]*, yet they fall short on providing a continent-wide assessment of existing, under construction and planned power plants, while accounting for their water use and CO2 emissions. In addition, it is of importance for researchers and stakeholders working in Africa to obtain open-access data, as they often lack the funds to purchase high quality data. This limits research efforts and resulting science-based decisions among stakeholders. Here we fill this data gap.*

While the Discussion is rich and wide-ranging, it currently reads largely as an interpretation of the authors' own results. I encourage the authors to more systematically relate key findings to prior empirical evidence and established conclusions in the literature. This would improve the scholarly integration of the paper and sharpen the interpretation of the results.

__Answer:__ We thank the reviewer for evaluating our discussion section as "rich and wide-ranging " We have added concise additional discussion by putting our results in the existing literature.

In total, in the discussion section, we include 29 unique literature sources to frame our discussion.

The manuscript should provide a clearer description of how the sample of 2,534 individual power plants was assembled and filtered. In addition, the authors should briefly discuss their representativeness.

__Answer:__ INVENTORY of 2,534 individual power plants is not a sample, it is the best available power plant database representing the most power plants assembled for Africa in any study. How we generated this database is explained in the Lines 69-80 and 624-646. Off course, it is clear that not all existing power plants are grasped in our study, as it is impossible to include all very small (e.g. small local hydropower) power plants. We would like to refer to Supplementary Table 1 to show how our study improves on existing studies in terms of representativeness:

- As an example, for the year 2020, the most recent year of WRI's Global Power Plant Database, we account for 2,534 power plants with a cumulative capacity of 245,604 MW, whereas WRI's Global Power Plant Database accounts for 631 power plants with a cumulative capacity of 160,533 MW. Consistently for all different fuel types we include many more power plants, except for nuclear, where both databases include 1 plant – as there is only 1 existing nuclear power plant in the RSA.

Because the projections extend only to 2030 (a relatively near-term horizon), the manuscript should more explicitly justify why 2030 is the central milestone. Moreover, the forecasting dataset appears to end in 2023. If 2024 (and possibly 2025) observed data are now available for at least a subset of countries/technologies, the authors could compare these realized values with the model's short-horizon predictions as a simple out-of-sample validation.

The delay assumptions (e.g., 1-year vs 2-year delays by technology classes, plus specific treatment for large hydro/nuclear) should be supported by either citations or empirical project-level evidence. In addition, please consider decomposing the sensitivity analysis into two or three simpler scenarios (e.g., "large hydro only," "fossil only," "wind/solar only") to help readers identify which technologies drive the uncertainty.

__Answer:__ We have added the statement "2030, *which is the target year of the SDGs*" (Line 60) in the Introduction. This relates also to the first paragraph of our paper, where we discuss the SDGs (Lines 36-42).

We have now added a comparison of INVENTORY with observed data for 2024 and 2025 in the paper, visible in the new Supplementary Table 8.

In the methodology we added Lines 829-834:

> *Years 2024 and 2025*
>
> *Our paper was first submitted in November 2024, while final publication occurred in 2026. We therefore present our analysis up to the year 2023 as existing data, and all successive years (2024 to 2030) as forecasted data. In the beginning of 2026, we are however able to identify the additional power plants becoming operational in the years 2024 and 2025 based on real world data. We compare the results in Supplementary Table 8.*

In the discussion section, we added Lines 536-546:

> *As our paper was first submitted in November 2024, we present our analysis up to the year 2023 as existing data, and all successive years (2024 to 2030) as forecasted data. In 2026 we were able to identify the additional power plants becoming operational in the years 2024 and 2025 based on real world data. We compare the results in Supplementary Table 8. In terms of total electricity produced, we compute in INVENTORY 1,222 GWh for 2024 and 1,251 GWh for 2025, whereas real world data are 1,161 GWh for 2024 and 1,220 GWh for 2025 (difference of 5% and 2%, respectively). These are relatively low differences, generally due to delays in power plants becoming operational or new power plants becoming operational not captured in INVENTORY. The latter is especially the case for power plants with short construction times such as solar and wind, as discussed before. The same can be observed for the amount of power plants, where the differences are 0 and -1% (Supplementary Table 8).*

On the comments regarding delays, we added in the methodology, Lines 808-815:

> *The ACP e.g. reports solar and wind projects to be delayed by an average of 13 months[73]. As an African example, the 120 MW Kairouan Solar Photovoltaic Project in Tunisia was originally planned to become operational in 2024[74], but this was delayed to 2025[75]. For all other power plants, which have on average longer planning and construction times[14], we assume a delay of two years. Fossil-fueled thermal plants show average construction times of almost 5 years[14], so adding two years is an increase of 40%. An example is the Egbema gas-fired power station in Nigeria, which in INVENTORY is identified to become operational in 2024[76], but was delayed to 2026[77].*

in the Discussion section, we revised the Lines 518-533 in that they capture this:

> *Assuming in INVENTORY delays of 1 year for solar power plants and wind farms to become operational, for all other power plants 2 years, and accounting for potential delays in nuclear and large hydropower plants, decreases the electricity production forecast from 1,787,858 GWh in 2030 to 1,504,848 GWh. Much of this decrease is due to the assumption that the large Congo river hydropower plants are delayed, i.e. the Grand Inga Power Station (152,321 GWh) and Inga III (40,075 GWh). Other large hydropower plants include the Mambilla power plant in Nigeria (11,777 GWh) and Mphanda Nkuwa in Mozambique (10,710 GWh). We also assume the two new Nigerian nuclear power plants to be delayed (17,636 GWh). This much delayed version of INVENTORY thus only adds up to 1,504,848 GWh (-16% compared to 1,787,858 GWh), an amount still higher than the highest time series regression analysis (REGR_POL 2030, 1,414,231 GWh, Fig 1). The delay of only these hydropower plants results in a reduction of 14%. The delay of only these nuclear power plants results in a reduction of 4%. Even for all these delayed versions of INVENTORY, it can be confirmed that Africa is stepping up in electricity*

production. As we most probably underestimate solar power plant and wind farm projects in INVENTORY for the later years up to 2030, our statement that Africa is stepping up remains valid.

The description of the workflow is internally inconsistent (e.g., the text refers to four steps but later mentions a “fifth forecast” and “step 5”). Please revise the Methods narrative so that the number of steps and the number of forecast scenarios are consistent throughout.

Answer: Thank you for this good observations. Indeed there are 5 steps and in total 6 forecasts. We have revised the text that this is now consistently described as such.
